# School’s out forever? Heavy metal preferences and higher education

**DOI:** 10.1371/journal.pone.0213716

**Published:** 2019-03-19

**Authors:** Martin Hällsten, Christofer Edling, Jens Rydgren

**Affiliations:** 1 Department of Sociology, Stockholm University, Stockholm, Sweden; 2 Department of Sociology, Lund University, Lund, Sweden; Tampereen Yliopisto, FINLAND

## Abstract

**Objectives:**

Cultural behaviors are theoretically linked to future life chances but empirical literature is scant. We use heavy metal as an example of cultural identities due to its high salience. We first assess the social morphology of metal preferences in terms of socio-economic and socio-structural positions, and then asses the short term outcomes of being a heavy metal fan on education and health behaviors.

**Methods:**

The analysis was based on a representative random stratified sample of 23-year-olds of native Swedish, Iranian, and Yugoslavian background in contemporary Sweden (n = 2,232). Linear probability models with multiple imputation were used to calculate preferences for metal music and the association of metal preferences with subsequent outcomes.

**Results:**

In contrast to many prior studies, we find that the preference for heavy metal is not structured by social background or neighborhood context in Swedish adolescents. Poor school grades tend to make them more prone to like metal, but net of previous grades, social background, personality, personal network, and neighborhood characteristics, metal fans have substantially lower transition rates into higher education.

**Discussion:**

The study suggest that metal preferences appears rather unsystematically with few important predictors, and is linked to lower education attainments in the short run. While these findings are specific to heavy metal as a certain type of culture and to Swedish adolescents, we suggest that they are indicative of how cultural consumption may play a role for life-chances.

## Introduction

Cultural preferences and behaviors are integral to adolescents’ lives. Cultural consumption is key to their social identities, hierarchically structuring their lifeworld. Adolescents’ cultural consumption is not only a reflection of their parents’ social standing, but also an effect of their own agency and is influence by their school and peer environment. Cultural consumption during the formative years of adolescence may have long-term consequences through the choice of identity and of friends and acquaintances, and may affect for example educational attainment and behavioral outcomes. Music is a pervasive and strong form of cultural expression that tends to be central to the development of identity, and also to the formation of subcultures.

Specifically, we focus on metal music. Heavy metal is an excellent example because of its high salience in terms of clothing, aggressive sound, and its often oppositional messages. Together with hip-hop, heavy metal is among the most disliked genres music by outsiders in some studies [1], and it has been linked to low educational ambitions [2–4]. We focus on adolescents (born in 1990) in contemporary Sweden, and analyze whether metal preferences are socially structured, and in turn whether they are associated with educational transitions and indicators of deviant behavior.

### The adolescent lifeworld

The social hierarchy among adolescents is arranged around group identities, which in turn circle around cultural elements. Indeed, consumption and taste are symbolic communications that structure relationships, networks, and status groups [5], and are subject to cultural distinction [6] and involve boundary making, i.e., exclusionary processes [7]. Akerlof and Kranton [8] have outlined a theoretical model in which adolescents’ social identities under some conditions leads to inequality in school performance. They argued that students adapt to the behaviors prescribed by their social category: adolescents derive utility from group membership and thus maximize coherence with group identity. Adolescents also accept costs in other outcomes in order to maintain a self-image coherent with that of the group. Such identity involves a set of norms, and these norms then structure outcomes. Given that the school provides the context, group norms often involve norms related to participation in teaching activities or for the expression of school ambition, either pro or anti.

In forming social identities, music is especially potent because it is pervasive and accessible at low cost. Much of the literature suggests that musical taste reflects individuals’ life experiences. For example, Arnett [9] argued that media consumption is a socializing agent separate from that, e.g., of family and school. Media, however, leave much greater room for voluntary choice based on individual predisposition, and media, in turn, may provide competing norms and values to that of family and school. The result is that media largely allow adolescents to self-socialize. Roe [10] similarly argued that adolescents’ use of socially disvalued media reflects their already subordinate positions within schools. Adolescents are thus likely to self-select into music subcultures that help them cope with their life experiences.

### The metal identity

Metal music is often described as a male white working class phenomenon that originated in industrial cities, where the hopelessness from socially excluded youth in industrial cities was given voice [11]. Some scholars emphasize the destructive side in metal discourse. Arnett (chapter 3 in [12]) analyzed metal songs from 1988–1992 and found that they primarily expressed anger or sadness, with violence as the most common theme. Heavy metal music is often aggressive music with dark lyrics and metal songs are about “despair, evil, and personal failure”[13]. Other scholars emphasize individualism and opposition as main components of metal music. For example, in the literature review of Wallach, Berger [14], metal is presented as an underdog music that centers on disenfranchised (youth) in either industrialized or de-industrialized contexts: it provides an escape from the dull everyday life of the lower ranks of society. Metal also functions as a catalyst for the rejection of parental and/or traditional values, and empowers its listeners. Even in globalized versions of metal outside the western worlds, one common denominator is metal’s culture of opposition that channel the need for young people to find an outlet to express anger and discontent [15, 16].

Metal is also often found to be a disliked genre in the US and the UK [1, 17], adding to its social exclusion, even though this may have become less salient over time for the most popular of metal genres, and there are some national contexts where this is not the case (e.g., Finland [18]). Heavy metal, whose fans, according to Bryson [1], tend to be less educated, are among the genres that are also most likely to be rejected by the musically tolerant. The negative image of metal, according to Bryson, thus evidences class-based exclusion. In a similar vein, a recent study by Brown and Griffin [19] have argued that middle-class cultural authority drives the negative image of metal in mainstream media, and because metal is generally excluded in the mainstream, it is more likely to feed opposition to a mainstream culture.

The message of metal music is however not necessarily an integral part of metal subculture. Even if a common perspective in metal discourse is that of white working class males, the social morphology of metal fans does not necessarily meet these criteria. A number of studies suggest that metal fans may come from privileged social classes [13, 17, 20]. The proliferation of the genre over time suggests that it will become more mainstream by sheer numbers. Metal music also often have positive effect on its listeners. Metal music has the effect of providing metal fans calm (chapter 4 in [12] and [21]) and a positive mood or affect [22, 23], and provides a resource that turns negative emotions into a creative outlet [24]. It is then less clear whether the negative messages of metal discourse will be endorsed and internalized by metal fans. However, In a Swedish study, Larsson [25] found that Swedish metal fans gave expression for social exclusion and rebelliousness as part of their metal identity. Another Swedish study [26] found that youths affiliating with radical adolescent peer crowds, such as Punks and Goths (which lie close to metal), were more inhibited than other youths, and adopted this identity as a coping strategy by limiting social contact.

### Metal and education

A long array of studies link heavy metal to low school achievements [3, 4, 27, 28]. On a discursive level, since metal carries a complex story of failure and revenge and opposition to mainstream society, metal culture may thus include antimobility norms, and catalyze and channel fatalism. Theories of group identity processes, e.g., Akerlof and Kranton [8], suggest that group identity often causes group member to adhere to group norms whatever messages they involve. To the extent that messages such as fatalism and opposition to the mainstream in metal becomes the nexus that attracts the non-mainstream and rejects the mainstream, educational values may decline. This suggests a situation similar to that of burnouts in Coleman’s classical work [29]. This idea is further developed by Ter Bogt, Keijsers [30], who argues that music identity has causal effects on behavior based on group closure and social contagion effects. In sum, this strand of theory suggests a causal effect of metal preferences on educational ambitions.

Another strand of studies acknowledges the link between metal and low educational ambitions, but suggest instead that selection is the main explanation. Roe [4] argues that the mechanism is anticipatory socialization, i.e., that it is individuals with lower expectations of the future that tend to select in to metal preferences. Metal thus channels low educational ambitions, rather than causally influencing them. Previous research from western societies suggest that metal attracts many adolescents with bad life experiences [9, 10, 26]. For example, in a US based qualitative study, Arnett [31] argues that while many people may casually like heavy metal, some degree of alienation is a precondition to be drawn into metal subculture. Metal music may thus be a way of expressing difficulties in life and coping with them. For example, Schwartz and Fouts [32] found that heavy metal listeners in Canada often had difficulties in negotiating personal and developmental issues, whereas more eclectic listeners had fewer such difficulties. In a Swedish study, Bešić and Kerr [26] found that youths affiliating with radical adolescent peer crowds, such as Punks and Goths, were more inhibited than other youths. A recent study in Canada however found mixed support for anticipatory socialization: metal fans were on the one hand advanced stream students that did not engage in oppositional practices of skipping and suspension, but on the other hand were less likely to feel that education is an important part of their lives [2].

### The social morphology of metal

An important question following on the selection perspective is the more general issue of who becomes a metal fan in terms of social positions. Since the discourse of metal lyrics is centered on white working-class males, the expectation is that listeners mirror this image. Research from mainly the US up to some 20 years ago indeed suggest that they come from the lower strata of society. Metal fans were more often working-class youths [33] who had lower levels of education [1, 34] and were more often white [35]. These factors are important competing explanations to consider, because they suggest that individuals may self-select into metal preferences. However, more recent studies from mainly Europe suggest that metal fans are found in all social strata of society. A representative survey in England suggest that metal fans are of from middle tiers of society [17]. Studies of festival going metal fans in e.g., Germany and France [20] suggest that they do not come from under-privileged backgrounds (yet, due to ticket prices, festival goes are positively selected on economic resources and therefore less representative). Also a recent study from the US showed that they were rather middle class [13], although the sample was also not representative.

There is also important national variation in the popularity of the genre, which may explain both how representative it is across social strata, and the degree to which it is disliked. A related issue is how stable metal’s cultural position is over time. Shaap and Berkers [36] document substantial variation over time in the legitimacy of metal subgenre’s in the Netherlands. One may expect that the larger the representation metal has in a country, the looser the links to social class and exclusionary processes. Metal has been observed to be unpopular in the US [1]. In contrast, Metal is rather popular in Finland, and it is also not very disliked in that context [18]. This can be compared to the UK, where metal is less liked than in Finland [37], but also much more disliked [17]. While no contemporary study of Sweden exists, one may expect that Sweden resembles its neighboring country Finland, not least due to Sweden’s thriving metal scene [38].

### Previous studies of the consequences of metal identity

The study of heavy metal fans and music now has a long history, with the moral panics linked to PMRC of the 1980s triggering great interest. The early studies linked metal preferences to various social behavioral problems [39], suicidal thoughts [22, 40], personality problems [32], machiavellianism (i.e., to manipulate, deceive, and exploit others to achieve their goals) and anti-authoritarianism [41], poor school performance [28], and a decreased desire to engage in effortful cognitive endeavors [41]. The earlier studies discussed the causality issue extensively, but this awareness was not reflected in the statistical analyses as they included only rudimentary controls. To give some examples of the previous literature: Arnett [39] controlled for two covariates: indices of sensation-seeking and family relations, and this explained the metal association in 7 out of 12 outcomes. Martin, Clarke [40] study of metal fans and psychological vulnerability provided only bivariate analyses; and this was also true of Hansen and Hansen [41]. Took and Weiss [28] controlled for one factor: gender. Scheel and Westfeld [22] provided a bivariate analysis only. Schwartz and Fouts [32] controlled for gender and grade level (in school). A good example is suicide cases that became public because parents sued metal bands. However, in these cases, the child had experienced troubles long before becoming a metal fan [42], leaving metal as an unlikely explanation of the suicide. However, a few recent studies [30, 43] have addressed selection more adequately than the early literature, but still found that metal was related to negative outcomes.

A limitation of previous studies is also that with few exceptions they are small n-studies on non-representative samples (e.g., high schools students in single schools). The previously cited studies had the following sample sizes: Arnett [39] n = 245, Martin, Clarke and Pearce [40] n = 354, Took and Weiss [28] n = 87, Scheel and Westfeld [22] n = 121, Schwartz and Fouts [32] n = 249, Hansen and Hansen [41] n = 102. Often schools are chosen randomly, and then their pupils are surveyed. But with only two sampling units, random variation will be very large and representativity may be questionable. The small sample size limits statistical power to identify group differences (which may indirectly influence the authors to disregard confounding factors).

In the only large random sample study (n = 4,159), Mulder, Bogt [43] found that heavy metal fans (the ‘exclusive rock’ cluster) among Dutch adolescents had higher levels of both internalizing and externalizing problems. Despite the large sample, due to the small group size of heavy metal fans, statistical power was not always sufficient to identify differences with other genres. The researchers controlled for gender, age, schooling, the family’s consumption of goods, and the quality of relationships with parents and peers.

More recently, Ter Bogt, Keijsers [30] examined the development of music preferences and delinquency over time in a random sample (n = 309) of Dutch youth. A preference for rebellious music was associated with higher delinquency rates. Net of gender, educational level, and personality, they found that initial music preferences rather than growth in preferences explained delinquency, which according to their argument favors a causal interpretation of music preference over self-selection. They relied on SEM estimation and time order of measures to identify causal effects, yet a potential weakness of the study was the lack of control for socio-economic factors.

A recent 30-year follow-up study (n = 377) of metal fans also showed that metal enthusiasts have experienced more traumatic and risky lives, but that metal identity also served as a protective factor against negative outcomes [13]. However, since the sample in this study was self-recruited, it is likely that it missed the more marginalized metal fans, and the analysis was, in line with the original literature, only bivariate and mainly descriptive, and did not take any confounding variables into account.

### Hypotheses

This study sets out to test the argument that the heavy metal subculture, like any subculture, is likely to exert an influence on its members. Since much of previous research suggest that the ethos of metal is oppositional to mainstream society and institutions, it will attract individuals with oppositional predispositions. We thus expect metal fans to be negatively selected based on family background, school performance, and personality. To the extent that metal identity maintains and reinforces these oppositional values, as would be predicted by group identity models [8] and closure/contagion specific to music consumption models [30], it will also have negative consequences on future socioeconomic careers, net of controls for selection.

## Material and methods

### Data

The data stem from the 2^nd^ wave of the Swedish survey *Social Capital and Labor Market Integration*: *A Cohort Study*, in which a gross sample of 5,659 adolescents born in 1990 was selected by Statistics Sweden from population registers for interview by telephone between January and March 2013. All respondents were above age 18 the time of interview (in fact, 22 or 23 years old). The Ethical Review Board of Stockholm approved the study (2008/580-31). The study aimed at analyzing contextual factors to explain inequality in young adults’ life chances, i.e., choices and behavior with consequences for education, labor market situation, health, and criminality. Apart from the survey the data include matched register data on residency during childhood, school records, and parents’ earnings, occupation and education and country of birth. Informed consent was obtained from all individual participants included in the study, including the right to access register information. The net sample included 2,244 interviews, hence a response rate of 39.7 percent.

The largest share of non-response was not-at-home non-response, 37.9 percent of the gross sample. 21.2 percent refused to participate. Response rates in Sweden have dropped in recent years mainly due to cash-card cell phones, which make the initial contact more difficult since the number is not in most cases listed in a telephone directory. The sample has the expected non-response bias, i.e., lower response rates among individuals from lower socio-economic strata and with lower school grades. We include these factors as controls in our regression models, which adjust for this observed non-response bias [44]. (One way to adjust for non-response biases is through sampling weights. However, Winship and Radbill [44] shows that it is not necessary to include weights in the regression estimator once the factors used to produce the weight are included as control variables. All those dimensions that has used to calculate post-stratification sampling weights in our study have been included as a control.).

One advantage of this study is that it contrasts the majority to two large immigrant groups. The sample is based on three different groups of Swedes born in 1990: (a) all individuals with at least one parent born in Iran; (b) 50 percent of all individuals with at least one parent born in former Yugoslavia; and (c) a simple random sample of 2,500 individuals with two Swedish-born parents. The Yugoslavian and Iranian groups are two quite different immigrant groups, representing the second (Yugoslavia) and fifth (Iran) largest immigrant groups in Sweden in 2009. Yugoslavs started coming to Sweden as semiskilled labor immigrants in the 1950s and 1960s and became permanent residents; however, the largest wave included refugees from the Balkan wars of the 1990s. The Iranians started coming to Sweden after the Iranian revolution in 1979, and during the Iran-Iraq war, and the majority arrived as political refugees. Many of them were highly educated; some belonged to the Kurdish minority. Both national Iranians and Kurds are embedded in highly developed organizational structures, and Sweden is one of the cultural centers of the Kurdish population in the world. In our sample, 33 percent of the respondents with parents born in Yugoslavia were born in Sweden, while that was the case for 73 percent of the respondents with Iranian-born parents.

The Yugoslavian group has a comparatively low level of education, whereas Iranians are on a par with the Swedish majority [45]. However, more Iranians and Yugoslavs tend to be employed in lower-class jobs in Sweden compared to the Swedish majority, with some advantage for the Iranians over Yugoslavs. In terms of class in the country of origin, the Iranians left rather privileged occupations behind, but this was not the case for Yugoslavs. In terms of social network relations, both Iranians and Yugoslavian youth tend on average to have ethnically mixed friendship networks and a better occupational network than the Swedish majority.

### Music preferences and identity

Previous literature has tended to analyze effects of music preferences, i.e., liking a band within a genre or naming a favorite genre. This may not be the same as having a music *identity*, i.e., whether one also identifies with the band or subculture. We test whether metal preferences alone or in conjunction with having a stated music identity have the same predictors and outcomes. We use two independent sets of questions to achieve this.

First, the respondents are queried for their favorite band or artist using an open ended question. The resulting text strings have then been coded into a measure of heavy metal preference. The principle has been to identify artists and bands with clear a categorical label of heavy metal, including their subgenres. Related genres like rock, etc., have been assigned to the reference category of more mainstream music, as have all ambiguous or borderline cases. For example, we view Iron Maiden and Metallica as a good examples of heavy metal in our data, and exclude, e.g., Jimi Hendrix, the Rolling Stones, but also Nickelback, Linkin Park, and Nirvana. We have also included some punk acts in the metal category (i.e., classical punk, but not newer American skate punk), not least because they would share similar oppositional ideals as metal. Needless to say, coding artists to genres involves boundary making. Our coding thus attempts to balance categorical homogeneity and specificity with achieving adequate statistical power in a small sample. We also coded categories for rap or hip-hop, not least because this genre may carry an alternative but similar oppositional message that should not be confounded with the reference category of more mainstream music, and since our sample has two immigrant stratums, we expect this to be their preferred music. Unfortunately, when we combine the information sources to get at hip-hop identity (see the next paragraph), only 1.5 percent of our data holds that identity. For this reason, we do not focus on hip-hop other than as a control variable. For hip-hop/rap, good examples in our data are Eminem, Tupac and Kanye West. This coding generates two dummies for metal and hip-hop/rap preferences, respectively.

Second, we query the respondents for their social sources of identity. The question we pose is: “If you described yourself, what would be the most important thing to mention?”followed by response categories that capture country of birth, gender, sexual orientation, political views, social class, interest in sports, and certain style of music. We repeat this question for 2^nd^ and 3^rd^ most important identity. We code music identity as a dummy for music being mentioned as either the 1^st^, 2^nd^ or 3^rd^ most important source. By combining these two sources of information, we can test whether metal preferences alone or in conjunction with an identity in music best explains outcomes. One could regard a conjunction as a stronger devotion to the subculture, and a disjunction as a more casual interest in the genre. In practice, we test for an interaction between metal preference and a music identity. Descriptives for music preferences and identity are shown in Table 1.

**Table 1:** Descriptives.

	Mean	(SD)	Min	Max	Number of imputed obs.
*Music preferences and identity*					
Heavy metal preference	0.078	(0.269)	0	1	0
Heavy metal preference × Identity in music	0.041	(0.199)	0	1	0
Rap/Hip-hop preference	0.048	(0.214)	0	1	0
Rap/Hip-hop preference × Identity in music	0.015	(0.121)	0	1	0
Identity in religion	0.125	(0.331)	0	1	0
Identity in nation	0.271	(0.445)	0	1	0
Identity in gender	0.250	(0.433)	0	1	0
Identity in politics	0.232	(0.422)	0	1	0
Identity in sexual orient.	0.072	(0.259)	0	1	0
Identity in social class	0.120	(0.325)	0	1	0
Identity in music	0.344	(0.475)	0	1	0
Identity in locality	0.261	(0.439)	0	1	0
Identity in ethnicity	0.096	(0.295)	0	1	0
Identity in sports team	0.245	(0.430)	0	1	0
Identity, none stated	0.223	(0.416)	0	1	0
*Control variables*					
Female	0.482	(0.500)	0	1	0
Iranian	0.221	(0.415)	0	1	0
Yugoslavian	0.301	(0.459)	0	1	0
Parents’ years of education	11.903	(2.274)	6	19	0
ln permanent income, 1990–2010	7.999	(0.375)	6.126	11.102	0
Parents’ Treiman score	39.209	(13.022)	13	78	0
High brow capital	1.121	(0.594)	0	3.337	92
Network, proportion unemployed friends	0.079	(0.185)	0	1	119
Network, proportion immigrant friends	0.356	(0.375)	0	1	121
Network, # victim friends	0.346	(0.679)	0	5	0
Network, distrust in friends (z)	-0.001	(0.997)	-0.749	2.936	121
Network, # close friends	3.194	(1.478)	0	5	0
ln # acquaintances	2.660	(0.843)	0	6.858	84
Social capital (z)	-0.001	(0.994)	-4.186	2.303	70
Neighborhood, ln pop. density	7.216	(2.100)	-1.064	10.367	0
Neighborhood, P in relative deprivation	0.126	(0.106)	0	0.594	0
Neighborhood, P foreign born	0.203	(0.145)	0	0.768	0
Fatalism	2.133	(0.602)	1	4.25	96
Self-confidence—Rosenberg	4.037	(0.832)	1	5	97
Self-control	3.472	(0.786)	1	5	96
GPA, 9th grade (z)	0.002	(1.000)	-3.254	1.678	73
Observations	2,232				
*Outcomes*					
Any TE enrolment (0/1) ^a^	0.514	(0.500)	0	1	
Aspirations scale ^b^	0.627	(0.249)	0	1	
Mental ill-being scale ^c^	0.258	(0.271)	0	1	
Smoking # cigs/day scale ^d^	0.041	(0.118)	0	1	
Binge drinking scale ^e^	0.378	(0.264)	0	1	
Smoked cannabis last year (0/1) ^f^	0.110	(0.314)	0	1	

Note: No imputations for outcome variables. The number of valid observations for them is as follows

2232^a^,

2137^b^,

2150^c^,

2231^d^,

2212^e^, and

2146^f^.

### Outcomes

Since our sample involves young adults where the majority is far from having completed their education or having entered the labor market, we analyze educational transitions as well as aspirations. Transition into higher education is based on registers matched to the survey data, where we code a binary measure based on records of any tertiary education enrolment. Aspirations are self-reported, and based on the items “It is important to get a well-paid job”and “I think it is important to go to university,” both with Likert response scales 1–5. The aspiration measure correlates .35 with the measure of any tertiary educational enrolments. We also analyze indicators of mental health problems, counting the prevalence of sleeping problems, anxiety, and depression. The response scale includes no, weak or severe problems, where the latter is weighted 1/6 and 1/3, respectively, and then summed so that the maximum is one. The scale can thus be interpreted as the proportion of the maximum number of problems. As indicators of risky health behaviors we used self-reports on smoking (cigarettes per day), binge drinking (a scale of its incidence), and whether one smokes marijuana (binary coded). These scales were also recoded to proportion of maximum value. Descriptives for the outcomes are shown at the bottom of Table 1.

### Independent variables

Our aim was to analyze the selection into heavy metal identity, and its association with the transition into university. Previous literature suggests that heavy metal identity is structured by social class and education, and therefore we include social background as defined by parents’ education (in years), occupation (measures as prestige scores [46]), and income in all models. These three SES measures are collected from registers. We also supplement this with a measure of the subjects own highbrow cultural capital since this is linked to a direct indicator of metal preference, namely school performance [47], and because metal typically holds a low position in a cultural value space [1]. Highbrow cultural capital measures the incidence of theater, museum, and library visits (but not of concert visits), reading habits and the number of books at home. As we just stated, poor school performance appears to be a central factor in the development a heavy metal identity [10], as is personality [30]. We thus include GPA from end of elementary school and measures self-confidence [48, 49], fatalism/external locus of control [50] and self-control [51] as predictors of heavy metal preferences and as controls in the outcome regressions.

We also control for social network and neighborhood characteristics, since to a large extent these will frame the conditions of the subjects’ lifeworld. Based on a name generator that extracts the names of up to five friends, defined as the “people that you spend most time with”and then asks about those peers’ characteristics, we measure the proportion of unemployed friends, immigrant friends, the number of crime victim friends, the average of ego’s distrust in friends, and the number of close friends. We remove peer relations based on kin, as we are not interested in traditional family of origin effects. We complement this with a measure of the number of (log) acquaintances one has. We measure neighborhood characteristics using matched data from population registers of neighborhoods (defined using the Swedish SAMS classification). We measure (the natural log, ln, of) population density, the proportion living in relative deprivation (proportion below half of median income), and the proportion of foreign born.

Finally, we also measure another facet of social networks, namely occupational social capital. Here, we use the *position generator* [52], where respondents were asked whether they knew anyone in 40 strategically chosen occupations. This approach identifies the latent information and resources embedded in a network. From the position generator, we have derived a number of dimensions (extensity, range of occupational prestige, highest ranked occupation, average and total accessed prestige, see [52, 53] that we include in factor analysis, where we use the first factor as our *composite* measure of social capital [54]. Descriptives for controls are shown in the mid panel of Table 1.

### Methods

We analyze the incidence of metal preferences and identity, which are discrete 0/1, as well as the outcomes, including those that are discrete 0/1 using linear regressions. In the discrete case, the model becomes a linear probability model. We avoid using non-linear models such as logit regressions as their coefficients are not comparable across samples and models due to scaling sensitivity [55], and thus make it very difficult to analyze interaction effects [56]. Linear probability models, on the other hand, produce consistent estimates of the expected value of the outcome conditional on covariates, i.e., E(Y|X) (chapter 3 in [57]). LPM coefficients are often similar to estimates of average marginal effects (AME, which are also scaling insensitive) from logistic regressions.

### Imputation of missing values

We have imputed missing values due to item non-response for a number of independent variables in order to keep the sample size intact and decrease the risk of biased results. We follow the multiple imputation strategy outlined by Rubin [58, 59]. We dropped cases entirely where register links where missing (n = 12), and then used the remaining independent covariates without missing information to predict and simulate values for those with missing information. We used M = 100 imputations (independently imputed datasets). Item non-response ranged up to a hundred cases out of the sample size of 2,232 for some variables, as shown in Table 1. We do not impute values for the dependent variables (and here, Table 1 instead shows their number of valid cases).

## Results

### The social structure of metal preferences

The top panel of Table 1 shows descriptive statistics for our indicators of music preferences and identities. We see that the incidence of metal music preferences is .078, i.e., that almost 8 percent of the sample have such musical tastes. The corresponding number for hip-hop tastes is 5 percent (.048). When we combine the music preferences with information regarding whether the individuals derive their identity from music (which applies to 34 percent of the sample) to get closer to heavy identities, we find that 4.1 percent of our sample have both metal preferences and an identity in music. This also means that 50 percent among metal listeners have a strong metal identity, and that the other 50 are more casual listeners.

In Table 2, we analyze how metal preferences are socially structured in a linear probability model. In Table 3 we show a combination of metal preference and music identity. Both analyses yield very similar results, although most associations become weaker for the more exclusive measure of metal identity. We focus on the former and more encompassing measure. In Model 1, we find that the metal preference is rather exclusive to males and native born, with strong negative coefficients for gender and Iranian/Yugoslavian background (of the 144 in our sample with metal preferences, only 31 are female; and 131 belong to the native sample). Models 1 through 3 contain the three components of social background separately, but surprisingly, we find no significant associations between parents’ education, income, or occupational prestige and metal preferences. In Model 4, they enter simultaneously and still have insignificant coefficients. Model 4 also contains the measure for highbrow capital, but this too is not related to metal preferences. Model 5 contains social network and neighborhood characteristics. Here, too, we find few factors that structure metal preferences in any significant way. The only factors with significant coefficients are social capital and the proportion of immigrant friends, which, unsurprisingly in light of the strong ethnic sorting of music preferences, tends to decrease the preference for heavy metal. The coefficient for occupational social capital is negative, meaning that more connected individuals are less likely to have metal preferences. The social capital measure is expressed as z-scores, so that one standard deviation is associated with a 1.5 percentage point lower incidence of metal preference. Given the overall incidence of 8 percent, this is a moderate-sized association.

**Table 2:** The social selectivity of metal preferences. Linear Probability Models.

	Heavy metal preference						
	(1)	(2)	(3)	(4)	(5)	(6)	(7)
Female	-0.096***	-0.095***	-0.095***	-0.096***	-0.092***	-0.091***	-0.087***
	(-8.821)	(-8.760)	(-8.812)	(-8.830)	(-8.304)	(-8.140)	(-7.739)
Iranian	-0.091***	-0.099***	-0.095***	-0.102***	-0.064***	-0.065***	-0.065***
	(-7.248)	(-7.264)	(-7.192)	(-7.333)	(-4.095)	(-4.145)	(-4.162)
Yugoslavian	-0.083***	-0.086***	-0.089***	-0.090***	-0.052**	-0.054**	-0.054**
	(-6.288)	(-6.494)	(-6.099)	(-6.125)	(-3.043)	(-3.142)	(-3.153)
Parents’ years of education	-0.001			0.002	0.003	0.003	0.004
	(-0.568)			(0.717)	(0.908)	(0.922)	(1.418)
Parents ln permanent income		-0.027		-0.023	-0.024	-0.025	-0.019
		(-1.871)		(-1.437)	(-1.489)	(-1.537)	(-1.153)
Parents’ Treiman score			-0.001	-0.001	-0.001	-0.001	-0.001
			(-1.494)	(-1.125)	(-1.255)	(-1.204)	(-1.070)
Highbrow capital				0.005	0.01	0.011	0.014
				(0.550)	(1.024)	(1.183)	(1.456)
Network, P unemployed friends					0.019	0.018	0.006
					(0.501)	(0.477)	(0.152)
Network, P immigrant friends					-0.068***	-0.069***	-0.071***
					(-4.126)	(-4.149)	(-4.234)
Network, # victim friends					0.003	0.003	0.002
					(0.301)	(0.279)	(0.242)
Network, distrust in friends (z)					0.009	0.009	0.009
					(1.516)	(1.586)	(1.523)
Network, # close friends					-0.001	-0.001	0
					(-0.182)	(-0.205)	(0.053)
ln # acquaintances					0.012	0.01	0.012
					(1.524)	(1.363)	(1.561)
Social capital (z)					-0.015*	-0.016*	-0.015*
					(-2.339)	(-2.444)	(-2.241)
Neighborhood, ln pop. density					-0.001	-0.001	-0.001
					(-0.345)	(-0.342)	(-0.213)
Neighborhood, P in rel. deprivation					-0.003	0.002	0.022
					(-0.045)	(0.035)	(0.349)
Neighborhood, P foreign born					-0.029	-0.031	-0.042
					(-0.551)	(-0.594)	(-0.806)
Fatalism						0.004	0.004
						(0.410)	(0.383)
Self-confidence						0.013	0.012
						(1.859)	(1.701)
Self-control						-0.006	-0.003
						(-0.768)	(-0.366)
GPA, 9th grade (z)							-0.017**
							(-2.660)
N	2,232	2,232	2,232	2,232	2,232	2,232	2,232
R^2^	0.055	0.057	0.056	0.056	0.065	0.065	0.067
Mean of outcome	0.078	0.078	0.078	0.078	0.078	0.078	0.078

Note: t-values in parenthesis,

* p<0.05,

** p<0.01,

*** p<0.001

**Table 3:** The social selectivity of metal preferences. Alternative specification. Linear Probability Models.

	Heavy metal preference × Identity in music ^a^						
	(8)	(9)	(10)	(11)	(12)	(13)	(14)
Female	-0.045***	-0.045***	-0.045***	-0.046***	-0.044***	-0.044***	-0.041***
	(-5.574)	(-5.526)	(-5.561)	(-5.573)	(-5.283)	(-5.215)	(-4.833)
Iranian	-0.054***	-0.059***	-0.057***	-0.062***	-0.034**	-0.034**	-0.034**
	(-5.954)	(-5.899)	(-6.017)	(-5.963)	(-3.049)	(-3.044)	(-3.059)
Yugoslavian	-0.046***	-0.048***	-0.052***	-0.053***	-0.026*	-0.027*	-0.027*
	(-4.660)	(-4.850)	(-4.795)	(-4.847)	(-2.185)	(-2.172)	(-2.181)
Parents’ years of education	-0.001			0.002	0.002	0.002	0.003
	(-0.689)			(0.916)	(1.068)	(1.049)	(1.499)
Parents ln permanent income		-0.018		-0.012	-0.013	-0.012	-0.008
		(-1.868)		(-1.146)	(-1.222)	(-1.149)	(-0.769)
Parents’ Treiman score			-0.001	-0.001	-0.001	-0.001	-0.001
			(-1.946)	(-1.654)	(-1.616)	(-1.594)	(-1.472)
Highbrow capital				0.004	0.007	0.007	0.009
				(0.562)	(0.946)	(1.045)	(1.288)
Network, P unemployed friends					-0.001	-0.003	-0.011
					(-0.033)	(-0.092)	(-0.393)
Network, P immigrant friends					-0.039**	-0.039**	-0.040**
					(-3.215)	(-3.202)	(-3.291)
Network, # victim friends					0.007	0.006	0.006
					(1.007)	(0.900)	(0.867)
Network, distrust in friends (z)					0.001	0.001	0.001
					(0.284)	(0.270)	(0.210)
Network, # close friends					-0.001	-0.001	0
					(-0.266)	(-0.184)	(0.040)
ln # acquaintances					0.005	0.005	0.006
					(0.876)	(0.801)	(0.970)
Social capital (VDG)					-0.009	-0.009	-0.008
					(-1.947)	(-1.883)	(-1.708)
Neighborhood, ln pop. density					-0.004	-0.004	-0.004
					(-1.343)	(-1.337)	(-1.233)
Neighborhood, P in rel. deprivation					-0.005	-0.003	0.01
					(-0.117)	(-0.067)	(0.213)
Neighborhood, P foreign born					-0.019	-0.021	-0.028
					(-0.490)	(-0.529)	(-0.709)
Fatalism						0.01	0.01
						(1.479)	(1.458)
Self-confidence						0.006	0.006
						(1.322)	(1.163)
Self-control						-0.004	-0.002
						(-0.718)	(-0.344)
GPA, 9th grade (z)							-0.011**
							(-2.580)
N	2,232	2,232	2,232	2,232	2,232	2,232	2,232
R^2^	0.027	0.028	0.028	0.028	0.033	0.033	0.035
Mean of outcome	0.041	0.041	0.041	0.041	0.041	0.041	0.041

^a^ the joint occurrence of the factors.

* p<0.05,

** p<0.01,

*** p<0.001

Model 6 contains the indicators of personality, i.e., fatalism, self-confidence, and self-control. A bit surprisingly in light of the theoretical background, none of these are significantly associated with metal preferences. Finally, Model 7 contains school grades from elementary school and we find that those with higher grades are less likely to become heavy metal fans. Indeed, this suggest a scenario in line with Roe [4] on anticipated socialization. The size of the coefficient is on a par with that of social capital, i.e., a standard deviation in the GPA distribution is associated with 1.7 percentage point lower incidence of metal preference.

Overall, the explained variation is between 5 and 7 percent in our models, which suggest that metal preferences is mainly explained by factors outside our model, including unobservable factors and randomness. This strongly suggest that metal preferences are not just a function of socially structured positions.

### The socioeconomic and health outcomes of metal preferences

Table 4 shows the association between heavy metal preference and the transition to university and career aspirations. The models also control for an alternative but socially similar music preference, namely hip-hop. Hence, the association of both these preferences should be compared to a mainstream music preference. Model 1 (5) is a baseline model without controls, Models 2 and 3 (6 and 7) sequentially add controls, while Model 4 (8) tests for an interaction between metal preferences and having an identity in music.

**Table 4:** Socioeconomic outcomes of metal preferences.

	Any Tertiary enrolments (0/1)				Aspiration scale			
	(1)	(2)	(3)	(4)	(5)	(6)	(7)	(8)
Heavy metal preference	-0.145***	-0.101**	-0.068*	-0.073	-0.042*	-0.028	-0.021	0
	(-3.695)	(-2.875)	(-2.047)	(-1.558)	(-2.014)	(-1.391)	(-1.083)	(0.003)
Rap/Hip-hop preference	-0.146**	-0.096*	-0.068	-0.06	-0.01	0.001	0.006	0.004
	(-3.078)	(-2.454)	(-1.756)	(-1.329)	(-0.382)	(0.031)	(0.260)	(0.147)
Heavy metal preference × Identity in music				0.011				-0.042
				(0.168)				(-1.094)
Rap/Hip-hop preference × Identity in music				-0.027				0.007
				(-0.321)				(0.136)
Gender, immigration	Yes	Yes	Yes	Yes	Yes	Yes	Yes	Yes
Identities	Yes	Yes	Yes	Yes	Yes	Yes	Yes	Yes
Parents’ SES		Yes	Yes	Yes		Yes	Yes	Yes
Networks		Yes	Yes	Yes		Yes	Yes	Yes
Segregation		Yes	Yes	Yes		Yes	Yes	Yes
Non-cognitive		Yes	Yes	Yes		Yes	Yes	Yes
GPA, 9th grade			Yes	Yes			Yes	Yes
N	2,232	2,232	2,232	2,232	2,137	2,137	2,137	2,137
R^2^	0.071	0.282	0.376	0.376	0.100	0.179	0.192	0.191
Mean of outcome	0.514	0.514	0.514	0.514	0.627	0.627	0.627	0.627

Note: t-values in parenthesis,

* p<0.05,

** p<0.01,

*** p<0.001

In Model 1, we see that metal preferences, but also hip-hop preferences, have a significant and rather strong negative association with transitions into university. The coefficients are very similar and show metal and hip-hop fans to have a 14.5 percentage point lower transition rate. The sample average transition rate is 51 percent, and so the effects are substantial, i.e., close to a 30 percent lower rate. Model 2 adds all controls except school grades, and this explain approximately ⅓ of the association, leaving a 10 percentage point difference between metal and hip-hop fans and the mainstream. Model 3 further adds school grades, which explain a small but substantial part of the association. Roughly half of the original association (net of immigration and gender) is thus explained by confounding factors. Metal fans thus have a 7 percentage points, or a 13 percent, lower transition rate compared to the sample mean (-.068/.514). This is still quite a substantial effect. For hip-hop, the coefficient is no longer significant in Model 3. This is perhaps not surprising given the substantially lower incidence of hip-hop fans in the sample (5 percent or 107 hip-hop fans, compared to 8 percent metal fans). It is likely that the statistical power is too low to adequately analyze hip-hop fans; future research on this may oversample immigrant groups to achieve sufficient power. However, within the focus of this paper, the hip-hop group was included as a control in order not to confound the mainstream category with other alternative music preferences. Model 4 tests for an interaction between metal preferences and having an identity in music. Our expectation was that this should delineate more hardcore fans from less committed fans, but the results (also those for selection into these groups in Tables 1 and 3) do not show any difference between the groups: the interaction terms are not statistically significant. Note that the non-significance of the main effect in Model 4 is indicative of too low power to assess the interaction, not an indication of lack of power in general; in the preferred Model 3, the coefficient is significant. We thus conclude that the metal preference more broadly is behind the lower transition rate.

Models 5 to 8 then analyze career aspirations. Here we see a similar pattern as in the transition to university for metal but not for hip-hop, where the effect is zero. In the baseline model, the aspirations are somewhat lower for metal fans (the mean of the scale is .627; see Table 1), but this difference vanishes when we add controls. Hence, metal fans and non-fans and hip-hop fans all have the same aspirations, but their realization of these aspirations in adolescence differs. There appears to be no interaction between music preferences and music identity.

In Table 5, we analyze health outcomes. To save space we show only the full model and the interaction model. In model 1, we analyze the number of states of mental ill-being, and there is no significant association with metal preferences. In Model 2, we analyze interactions between bases of identity and music preferences, but again none is statistically significant. In Models 3 and 5, we analyze smoking and binge drinking, respectively. We find that metal fans do not smoke or drink more than others, while hip-hop fans have slightly higher value on these scales, net of all the controls. The interaction Models 4 and 6 do not suggest that a music identity alters this conclusion much. In Model 7, we analyze cannabis smoking and find this incidence to be lower among metal fans, by almost 5 percentage points. The relative effect is rather substantial since the baseline proportion is 11 percent, thus close to halving the incidence. Again, the music identity interaction is non-significant. In sum, the strongest conclusion for metal fans is that they do not enroll in university and tend to stay away from marijuana; their other health behaviors are on a par with those having mainstream musical tastes.

**Table 5:** Health outcomes of metal preferences.

	Mental ill-being scale		Smoking # cigs/day scale		Binge drinking scale		Smoked cannabis last year (0/1)	
	(1)	(2)	(3)	(4)	(5)	(6)	(7)	(8)
Heavy metal preference	0.008	0.039	-0.005	-0.016	-0.005	-0.002	-0.048*	-0.035
	(0.430)	(1.381)	(-0.531)	(-1.514)	(-0.271)	(-0.069)	(-2.144)	(-1.192)
Rap/Hip-hop preference	-0.006	-0.006	0.036*	0.028	0.054*	0.066*	0.076	0.05
	(-0.242)	(-0.198)	(2.044)	(1.491)	(1.975)	(2.089)	(1.940)	(1.138)
Heavy metal preference × Identity in music		-0.06		0.022		-0.007		-0.024
		(-1.632)		(1.293)		(-0.206)		(-0.560)
Rap/Hip-hop preference × Identity in music		0		0.025		-0.038		0.085
		(-0.002)		(0.583)		(-0.643)		(0.954)
Gender, immigration	Yes	Yes	Yes	Yes	Yes	Yes	Yes	Yes
Identities	Yes	Yes	Yes	Yes	Yes	Yes	Yes	Yes
Parents’ SES	Yes	Yes	Yes	Yes	Yes	Yes	Yes	Yes
Networks	Yes	Yes	Yes	Yes	Yes	Yes	Yes	Yes
Segregation	Yes	Yes	Yes	Yes	Yes	Yes	Yes	Yes
Non-cognitive	Yes	Yes	Yes	Yes	Yes	Yes	Yes	Yes
GPA, 9th grade	Yes	Yes	Yes	Yes	Yes	Yes	Yes	Yes
N	2,150	2,150	2,231	2,231	2,212	2,212	2,146	2,146
R^2^	0.221	0.221	0.104	0.104	0.252	0.252	0.099	0.099
Mean of outcome	0.258	0.258	0.041	0.041	0.378	0.378	0.110	0.110

Note: t-values in parenthesis,

* p<0.05,

** p<0.01,

*** p<0.001

## Discussion

We find that metal preferences among young Swedes are not structured by socioeconomics and social structure. The only key predictors of being a metal fan that we find are gender (being male), immigration status (having native-born parents), having few immigrants in one’s personal networks, the extent and quality of one’s occupational social capital, and previous school grades. Parents’ socioeconomic resources, subjects’ cultural capital, network characteristics other than immigrant density, neighborhood characteristics, and personality do not predict metal preferences. The outcome of metal preferences is a lower transition rate into university by age 23. Net of all confounding factors, the metal fans had a 7 percentage points, or 13 percent, lower transition rate than those having mainstream preferences. We did not find any effects on career aspirations, or health outcomes (except a lower, not higher, prevalence of cannabis smoking). We also investigated whether a combination of metal preferences and indicators of having a stronger sense of identity coming from music moderates these findings, but this appears to not be the case. Hence, it is the preference for metal, regardless of any other identifications and regardless of strength of the devotion to metal, that drives the results. Our definition of metal is broad and should be seen as an average across many subgenres. Since many subgenres have more extreme ideologies (but with low representation in our sample), it is highly likely that metal has heterogeneous effects, even though our design does not allow us to decipher this further.

In contrast to previous studies, with few exceptions such as Mulder, Bogt [43], our study is from a representative sample of a size on a par with most survey research. The representativeness issue is central since we found metal to be less socially structured than previous research has suggested. The previous findings could thus be an artifact on non-random sampling. The sample size issue is important because even if our power was fairly large (n = 2,232), we are on the verge of power requirements. The t-statistic for our most central association, between metal preference and higher education, is close to 2. It is thus not surprising that previous literature has by and large failed to address confounding factors as it would have been a futile enterprise. Still, future research should try to avoid using small samples since this severely limits the ability to identify associations with any precision; 2,000 observations is likely a lower limit, but more serious power calculations should be made. In order to address heterogeneity within subgenres of metal, more designated sampling designs needs to be employed.

There are three ways of understanding the negative association between music preferences and university entry. First, the selection perspective suggest that these reflect (yet unobserved) dispositions that influence choice of music [9, 10, 32]. Second, others, like the Music Market Theory [30], instead suggest that music has independent, causal, effects on individuals’ future life chances. Here one might think of heavy metal identity as a form of anti-(highbrow) cultural capital (compare [47]. Both perspectives can be correct to varying degrees, i.e., when a music subculture reinforces behaviors and dispositions that were related to selection into that culture [9]. Our study advances the literature by using extensive sets of controls, i.e., of socio-economic status, personality, educational achievement, but also peer and neighborhood characteristics. The association remains. However, we cannot rule out that there is an important confounder that is not in our design, such as a predisposition of some kind that increases the chances of both liking heavy metal *and* not entering university, and the question of which of these interpretations is correct is also reflected in this paper. Nonetheless, the conditionally negative association between metal identity and the transition into higher education strongly suggests that metal identity is intertwined with processes of marginalization, either by channeling anti-educational attitudes or by just imparting fatalist perspectives.

A third alternative is that a heavy metal identity involves engagement in activities that simply outcompete education (or job seeking), i.e., playing in a band, going to concerts and festivals, and hanging out. For example, Arnett [21] showed that metal fans were more likely to play an instrument (the guitar) compared to a reference population. The longer-term effects of heavy metal identity may thus weaken as this type of youth life style typically comes to an end, and individuals engage in other pursuits. This should be especially the case in Sweden, where there are few if any dead ends in education. Our results did not show any differences in aspirations, and it may well be that the metal fans will catch up later in life. Only a longitudinal investigation (with a non-selective sample and non-retrospective design) can answer this question.

While our study focuses on heavy metal preferences and identity, our interest is more general. We see heavy metal identity as an easy way to operationalize social subgroup identities and the consequences of cultural stratification in youth, not least because metal culture is rather salient. We view our main contribution as showing that social inequality is not only generated or mediated by “hard” structural factors such as parental socio-economic status and social segregation, but also by “soft” social interactional factors, as consequences of cultural stratification. It is reasonable that the lifeworld of adolescents and the positions they take within this structure have long-term consequences and influence the trajectories of their future lives. This is an example of how inequality research and cultural studies may be combined to enrich each other and provide a more complete analysis in their respective domain.

## Supporting information

S1 File Survey SWE.docx. The survey in Swedish.
(DOCX)

S2 File Survey ENG.docx. The survey machine translated to English.
(DOCX)
