# Supplementary material for: School’s out forever? Heavy metal preferences and higher education
Source: PLoS One. 2019 Mar 19;14(3):e0213716. doi: 10.1371/journal.pone.0213716 (PMC6424403; doi:10.1371/journal.pone.0213716)
Supplement: S1 File — The survey in Swedish. (DOCX) [file pone.0213716.s001.docx]

**MEDLYSS2**

**VISAS EJ PÅ SKÄRMEN!**

**OBS! Denna fråga måste ligga som FÖRSTA fråga i alla formulär.**

**För dokumentation av formuläret, bläddra fram till fråga ETT.**

**2 = Stänger av inspelningen**

**______________________________________________________________________________**

**TILL BLANKETTKONSTRUKTÖR:**

**Om den får värdet 2 någon gång under intervjun så stängs inspelningen av.**

**(Beräkna MEDLYSS2 = 2 om MEDLYSS1 = 1; *** Stäng av inspelningen )**

**______________________________________________________________________________**

**_______________________________________________________________________________**

**MEDLYSS3**

**VISAS EJ PÅ SKÄRMEN!**

**OBS! Denna fråga måste ligga som ANDRA fråga i alla formulär.**

**1 Ja, det går bra (INSPELNING)**

**2 Nej, samtycker inte (EJ INSPELNING)**

**3 Avböjer intervju helt efter fråga om inspelning**

**4 Indirekt intervju (EJ INSPELNING)**

**5 IVE har ej TRIO**

**_________________________________________________________________________________**

**TILL BLANKETTKONSTRUKTÖR:**

**Lagrar svar från MEDLYSS1. Värden 1 - 3, 5 tilldelas från MEDLYSS1 och värde 4 från IDKOLL.**

**_________________________________________________________________________________**

**_______________________________________________________________________________**

**MEDLY**

**VISAS EJ PÅ SKÄRMEN!**

**OBS! Denna fråga måste ligga som TREDJE fråga i alla formulär.**

**1 MEDLYSS1 visas men ingen inspelning sker**

**2 MEDLYSS1 visas och inspelning sker vid samtycke**

**______________________________________________________________________________**

**TILL BLANKETTKONSTRUKTÖR:**

**Lagrar värde från bakgrundsvariabeln FORS. Värdet tilldelas från IDKOLL.**

**______________________________________________________________________________**

**_______________________________________________________________________________**

**MEDLYINFO**

**VISAS EJ PÅ SKÄRMEN!**

**För att medlyssningen ska fungera måste formuläret innehålla följande 5 frågor:**

**MEDLYSS1 Frågan ska komma upp om bakgrundsvariabeln FORS = '01', '02'.**

**Frågan kan ha något av följande värden**

**1 = Samtycke till inspelning**

**2 = Ej samtycke till inspelning**

**3 = Avböjer intervju**

**4 = INDIREKT INTERVJU. Frågan om samtycke ställs då aldrig och värdet 4 tilldelas.**

**5 = IVE har ej TRIO**

**MEDLYSS2 Dummy-fråga. Den får värdet 2 när inspelningen ska stängas av.**

**Om den inte har något värde, pågår inspelningen tills telefonsamtalet bryts.**

**MEDLYSS3 Dummy-fråga. Här lagras svaret från MEDLYSS1. Värde**

**MEDLYSS4 Dummy-fråga. Frågan visas om MEDLYSS1 = 3 .**

**Intervjuaren uppmanas att anbryta intervjun och koda bortfall.**

**(Resultatkod 60 med kommentar INSPELNING.)**

**MEDLY Dummy-fråga. Lagrar värdet från bakgrundsvariabeln FORS.**

**1 om FORS = '01'**

**2 om FORS = '02'**

**samt fråga VEM Här anges med vem intervjun sker, dvs om det är direkt eller indirekt intervju.**

**Inspelning ska ske endast vid direkt intervju**

**_______________________________________________________________________________**

**ETT**

**VISAS EJ PÅ SKÄRMEN!**

**Undersökning: Socialt kapital, våg 2**

**Undersökningsledare: Malin Forsberg**

**Produktionsansvarig: Emma Agerberg**

**Formulärkonstruktör: Slavica Dimic Kozarac**

**Tidsåtgång: ca xx tim**

**Fältarbete: 2013, vecka 2 --**

**-------------------------------------------------------------------------------------------------------------------------------**

**2012-10-30 SDK Formuläret påbörjat.**

**2013-01-14 SDK Produktionsversion skapad: Soc_Kap_vag2.**

**2013-01-16 SDK Ny prod.version skapad.**

**Nya svarsalt tillagda i fråga SLUT.**

**2013-01-22 SDK Ny prod.version skapad. Nytt svarslängd i fråga F236B.**

**-------------------------------------------------------------------------------------------------------------------------------**

**_______________________________________________________________________________**

**STARTDATUM**

**VISAS EJ PÅ SKÄRMEN!**

**Här lagras datumet (ÅÅÅÅMMDD) då intervjun startade.**

**Datumet sättsi fråga VEM.**

**_______________________________________________________________________________**

**SLUTDATUM**

**VISAS EJ PÅ SKÄRMEN!**

**Här lagras datumet (ÅÅÅÅMMDD) då intervjun startade.**

**Datumet sätts i fråga SLUT.**

**_______________________________________________________________________________**

**STARTTID**

**VISAS EJ PÅ SKÄRMEN!**

**Här lagras intervjuns starttid (TTMMSS).**

**Tiden sätts i fråga VEM.**

**_______________________________________________________________________________**

**SLUTTID**

**VISAS EJ PÅ SKÄRMEN!**

**Här lagras intervjuns sluttid (TTMMSS).**

**Tiden sätts i fråga SLUT.**

**_______________________________________________________________________________**

**KON**

**VISAS EJ PÅ SKÄRMEN!**

**Här lagras urvalspersonens kön.**

**1 = KVINNA**

**2 = MAN**

**_______________________________________________________________________________**

**T1**

**FRÅGAN ANVÄNDS ENDAST I ÖVNINGSFORMULÄRET!**

**Ange UP:s fullständiga namn (MAX 35 TECKEN):**

**_______________________________________________________________________________**

**T2**

**FRÅGAN ANVÄNDS ENDAST I ÖVNINGSFORMULÄRET!**

**Ange UP:s födelseår (ÅÅÅÅ):**

**_______________________________________________________________________________**

**T3**

**FRÅGAN ANVÄNDS ENDAST I ÖVNINGSFORMULÄRET!**

**Ange UP:s födelsemånad med två siffror (MM):**

**_______________________________________________________________________________**

**T4**

**FRÅGAN ANVÄNDS ENDAST I ÖVNINGSFORMULÄRET!**

**Ange UP:s födelsedag med två siffror (DD):**

**_______________________________________________________________________________**

**T5**

**FRÅGAN ANVÄNDS ENDAST I ÖVNINGSFORMULÄRET!**

**Ange UP:s kön:**

**1 Man**

**2 Kvinna**

**_______________________________________________________________________________**

**T6**

**FRÅGAN ANVÄNDS ENDAST I ÖVNINGSFORMULÄRET!**

**Gjordes det någon intervju med UP under våg 1 (för tre år sedan)?**

**1 Ja**

**2 Nej**

**_______________________________________________________________________________**

**T7**

**FRÅGAN ANVÄNDS ENDAST I ÖVNINGSFORMULÄRET!**

**Ange UP:s etniska bakgrund:**

**1 Jugoslavisk**

**2 Iransk**

**3 Svensk**

**_______________________________________________________________________________**

**VEM**

**VEM INTERVJUAS?**

**1 UP - PÅ SVENSKA**

**2 UP - PÅ ANNAT SPRÅK (UTAN TOLK)**

**3 ANNAN PERSON**

**_______________________________________________________________________________**

**IDKOLL**

**Först vill jag försäkra mig om att intervjun sker med rätt person.**

**Stämmer det du är född den <<TEXT FodDagTxt >> <<TEXT FodManadTxt >> <<TEXT FodAr >>?**

**1 JA**

**NEJ ---> AVBRYT INTERVJU**

**_________________________________________________________________________**

**TILL IVE: OBS! Svarsalternativen ska läsas upp endast där det uttryckligen står så.**

**WinDATI:s standardsvar kan anges i de flesta frågor: F8 = Vet ej**

**F9 = Vill ej svara**

**OBS! Tangent F3 kan oftast inte användas i detta formulär, använd i stället F7.**

**Symbolen :: visar att det finns instruktion till intervjuaren under F4 (Visa hjälp).**

**_________________________________________________________________________**

**_______________________________________________________________________________**

**MEDLYSS1**

**________________________________________________________________________**

**| |**

**| TILL INTERVJUAREN: |**

**| |**

**| Om du inte har TRIO installerat i Din dator, ska frågan INTE ställas till UP. |**

**| |**

**| Tryck 5 och ENTER för att fortsätta. |**

**|_______________________________________________________________________ |**

**Vi arbetar ständigt med att förbättra frågor och intervjuteknik i våra undersökningar**

**och därför vill vi, om du samtycker, spela in den här intervjun.**

**Inspelningen skyddas av sekretesslagen och personuppgiftslagen. Vi som jobbar**

**med undersökningen har tystnadsplikt och inspelningen förstörs inom 3 månader.**

**Går det bra för dig att vi spelar in den här intervjun?**

**_________________________________________________________________________**

**TILL IVE: I SCB:s statistikverksamhet skyddas uppgifter om enskilda personer, företag**

**och andra organisationer av sekretess. Statistiksekretessen gäller också hos**

**andra myndigheter med särskild statistikverksamhet. (För mer info, tryck F4.)**

**_________________________________________________________________________**

**1 JA, DET GÅR BRA**

**2 NEJ, SAMTYCKER INTE**

**3 AVBÖJER INTERVJU HELT EFTER FRÅGA OM INSPELNING**

**_______________________________________________________________________________**

**MEDLYSS4**

**AVBRYT INTERVJUN!**

**_________________________________________________________________**

**TILL IVE: Klarkoda med resultatkod 60 och**

**skriv "Inspelning" som kommentar till resultatkoden.**

**_________________________________________________________________**

**_______________________________________________________________________________**

**F10A**

**Jag tänkte börja med att fråga om du är född i Sverige eller i ett annat land?**

**1 Sverige**

**2 Annat land**

**_______________________________________________________________________________**

**F10B**

**Är någon av dina föräldrar född utomlands?**

**1 Ja, pappa**

**2 Ja, mamma**

**3 Ja, båda**

**4 Nej, ingen av dem**

**_______________________________________________________________________________**

**F10C**

**Är dina föräldrar födda i samma land?**

**1 Ja, samma land**

**2 Nej, olika länder**

**_______________________________________________________________________________**

**F11A**

**Under de senaste tolv månaderna, har du huvudsakligen bott ensam eller med någon annan?**

**1 Ensam**

**2 Tillsammans med någon**

**_______________________________________________________________________________**

**F11BS1**

**VISAS EJ PÅ SKÄRMEN!**

**Här lagras svar från F11B:**

**1 Föräldrar**

**_______________________________________________________________________________**

**F11BS2**

**VISAS EJ PÅ SKÄRMEN!**

**Här lagras svar från F11B:**

**2 Syskon**

**_______________________________________________________________________________**

**F11BS3**

**VISAS EJ PÅ SKÄRMEN!**

**Här lagras svar från F11B:**

**3 Släktingar som inte är föräldrar eller syskon**

**_______________________________________________________________________________**

**F11BS4**

**VISAS EJ PÅ SKÄRMEN!**

**Här lagras svar från F11B:**

**4 Partner**

**_______________________________________________________________________________**

**F11BS5**

**VISAS EJ PÅ SKÄRMEN!**

**Här lagras svar från F11B:**

**5 Kompis**

**_______________________________________________________________________________**

**F11B**

**Under de senaste tolv månaderna, har du huvudsakligen bott tillsammans med**

**kompis, syskon, en partner eller med dina föräldrar?**

**FLERA SVAR KAN ANGES. TRYCK MELLANSLAG MELLAN SVAREN.**

**1 Föräldrar**

**2 Syskon**

**3 Släktingar som inte är föräldrar eller syskon**

**4 Partner (inkl pojkvän / flickvän, make / maka, sambo)**

**5 Kompis**

**_______________________________________________________________________________**

**F12A**

**Vilken typ av boende bor du i?**

**1 Förstahandskontrakt för lägenhet / villa / radhus**

**2 Andrahandskontrakt för lägenhet / villa / radhus**

**3 Bostadsrättslägenhet**

**4 Villa**

**5 Radhus**

**6 Studentrum / -lägenhet**

**7 Annat**

**_______________________________________________________________________________**

**F12B**

**Hur länge får <<TEXT 'du' om F11A = 1 >><<TEXT 'ni' om F11A^= 1 >> bo där?**

**____________________________________________________**

**TILL IVE: Ange svaret i antal månader från inflyttning.**

**Om mindre än 1 månad, ange 0.**

**____________________________________________________**

**ANTAL MÅNADER:**

**_______________________________________________________________________________**

**F12C**

**Hur har du fått tag i ditt boende?**

**1 Köpt av mäklare**

**2 Köpt svart**

**3 Tips via kontakter**

**4 Bostadskön**

**5 Via förmedlingstjänst, t ex på internet**

**6 Genom annonser**

**7 Via medlemskap i kooperativ förening**

**8 Via socialkontoret**

**9 Via studentkåren/nation**

**10 Annat**

**_______________________________________________________________________________**

**F21**

**Hur många syskon har du?**

**ANGE ANTAL:**

**_______________________________________________________________________________**

**F22**

**Har du fast sällskap eller är du gift?**

**1 Nej, har varken partner / fast sällskap eller är gift**

**2 Ja, har partner / fast sällskap (inkl pojk- / flickvän eller sambo)**

**3 Ja, gift**

**_______________________________________________________________________________**

**F23**

**Hur många egna barn har du?**

**OM INGA EGNA BARN, ANGE 0.**

**ANTAL BARN:**

**_______________________________________________________________________________**

**F24A**

**Är det ditt biologiska barn?**

**1 Ja**

**2 Nej**

**_______________________________________________________________________________**

**F24B**

**Hur många är dina biologiska barn?**

**OM INGA BIOLOGISKA BARN, ANGE 0.**

**ANTAL BIOLOGISKA BARN:**

**_______________________________________________________________________________**

**F31**

**Har din mamma eller pappa studerat vid universitet eller högskola i Sverige?**

**1 Ja, pappa**

**2 Ja, mamma**

**3 Ja, båda**

**4 Nej, ingen av dem**

**_______________________________________________________________________________**

**F32**

**Har din mamma eller pappa studerat vid universitet eller högskola i något annat land?**

**1 Ja, pappa**

**2 Ja, mamma**

**3 Ja, båda**

**4 Nej, ingen av dem**

**_______________________________________________________________________________**

**F33OPA1**

**Vad har din far (styvfar) haft för huvudsakligt yrke eller sysselsättning**

**under sin tid i Sverige fram till idag?**

**~~~~~~~**

**________________________________________________________________________**

**TILL IVE: Om UP:s far (styvfar) har haft en sysselsättning som inte finns med i listan,**

**t.ex. arbetslös, studerande, sjukskriven, ekonomiskt oberoende m.m.**

**ange 1 och skriv UP:s svar i klartext, hellre än att "tvinga in" svaret i listan.**

**________________________________________________________________________**

**1 = YRKET / SYSSELSÄTTNINGEN FINNS EJ I LISTAN**

**YRKE / SYSSELSÄTTNING:**

**_______________________________________________________________________________**

**F33OPA1TXT**

**( Vad har din far (styvfar) haft för huvudsakligt yrke eller sysselsättning**

**under sin tid i Sverige fram till idag? )**

**~~~~~~~**

**___________________________________________________________________________**

**TILL IVE: Försök ta reda på följande**

**- Huvudsakliga arbetsuppgifter**

**- Bransch**

**- Verksamhet (ex hemtjänst äldreomsorg, bygg inom mur och puts)**

**- Kvalifikationsnivå (ex ledningsarbete, militärt arbete, arbetare och tjänstemän)**

**___________________________________________________________________________**

**ANGE YRKE / SYSSELSÄTTNING I KLARTEXT:**

**_______________________________________________________________________________**

**F33OPA2**

**Vad hade din far (styvfar) för huvudsakligt yrke eller sysselsättning**

**innan han flyttade till Sverige?**

**~~~~**

**________________________________________________________________________**

**TILL IVE: Om UP:s far (styvfar) har haft en sysselsättning som inte finns med i listan,**

**t.ex. arbetslös, studerande, sjukskriven, ekonomiskt oberoende m.m.**

**ange 1 och skriv UP:s svar i klartext, hellre än att "tvinga in" svaret i listan.**

**________________________________________________________________________**

**1 = YRKET / SYSSELSÄTTNINGEN FINNS EJ I LISTAN**

**YRKE / SYSSELSÄTTNING:**

**_______________________________________________________________________________**

**F33OPA2TXT**

**( Vad hade din far (styvfar) för huvudsakligt yrke eller sysselsättning**

**innan han flyttade till Sverige? )**

**~~~~**

**___________________________________________________________________________**

**TILL IVE: Försök ta reda på följande**

**- Huvudsakliga arbetsuppgifter**

**- Bransch**

**- Verksamhet (ex hemtjänst äldreomsorg, bygg inom mur och puts)**

**- Kvalifikationsnivå (ex ledningsarbete, militärt arbete, arbetare och tjänstemän)**

**___________________________________________________________________________**

**ANGE YRKE / SYSSELSÄTTNING I KLARTEXT:**

**_______________________________________________________________________________**

**F33OMA1**

**Vad har din mor (styvmor) haft för huvudsakligt yrke eller sysselsättning**

**under sin tid i Sverige fram till idag?**

**~~~~~~~**

**__________________________________________________________________________**

**TILL IVE: Om UP:s mor (styvmor) har haft en sysselsättning som inte finns med i listan,**

**t.ex. hemmafru, arbetslös, studerande, sjukskriven, ekonomiskt oberoende m.m.**

**ange 1 och skriv UP:s svar i klartext, hellre än att "tvinga in" svaret i listan.**

**__________________________________________________________________________**

**1 = YRKET / SYSSELSÄTTNINGEN FINNS EJ I LISTAN**

**YRKE / SYSSELSÄTTNING:**

**_______________________________________________________________________________**

**F33OMA1TXT**

**( Vad har din mor (styvmor) haft för huvudsakligt yrke eller sysselsättning**

**under sin tid i Sverige fram till idag? )**

**~~~~~~~**

**___________________________________________________________________________**

**TILL IVE: Försök ta reda på följande**

**- Huvudsakliga arbetsuppgifter**

**- Bransch**

**- Verksamhet (ex hemtjänst äldreomsorg, bygg inom mur och puts)**

**- Kvalifikationsnivå (ex ledningsarbete, militärt arbete, arbetare och tjänstemän)**

**___________________________________________________________________________**

**ANGE YRKE / SYSSELSÄTTNING I KLARTEXT:**

**_______________________________________________________________________________**

**F33OMA2**

**Vad hade din mor (styvmor) för huvudsakligt yrke eller sysselsättning**

**innan hon flyttade till Sverige?**

**~~~~**

**__________________________________________________________________________**

**TILL IVE: Om UP:s mor (styvmor) har haft en sysselsättning som inte finns med i listan,**

**t.ex. hemmafru, arbetslös, studerande, sjukskriven, ekonomiskt oberoende m.m.**

**ange 1 och skriv UP:s svar i klartext, hellre än att "tvinga in" svaret i listan.**

**__________________________________________________________________________**

**1 = YRKET / SYSSELSÄTTNINGEN FINNS EJ I LISTAN**

**YRKE / SYSSELSÄTTNING:**

**_______________________________________________________________________________**

**F33OMA2TXT**

**( Vad hade din mor (styvmor) för huvudsakligt yrke eller sysselsättning**

**innan hon flyttade till Sverige? )**

**~~~~**

**___________________________________________________________________________**

**TILL IVE: Försök ta reda på följande**

**- Huvudsakliga arbetsuppgifter**

**- Bransch**

**- Verksamhet (ex hemtjänst äldreomsorg, bygg inom mur och puts)**

**- Kvalifikationsnivå (ex ledningsarbete, militärt arbete, arbetare och tjänstemän)**

**___________________________________________________________________________**

**ANGE YRKE / SYSSELSÄTTNING I KLARTEXT:**

**_______________________________________________________________________________**

**F33**

**Om du ser tillbaka på din uppväxttid, dvs fram till idag, vad har din far (styvfar)**

**haft för huvudsakligt yrke eller sysselsättning?**

**________________________________________________________________________**

**TILL IVE: Om UP:s far (styvfar) har haft en sysselsättning som inte finns med i listan,**

**t.ex. arbetslös, studerande, sjukskriven, ekonomiskt oberoende m.m.**

**ange 1 och skriv UP:s svar i klartext, hellre än att "tvinga in" svaret i listan.**

**________________________________________________________________________**

**1 = YRKET / SYSSELSÄTTNINGEN FINNS EJ I LISTAN**

**YRKE / SYSSELSÄTTNING:**

**_______________________________________________________________________________**

**F33TXT**

**( Om du ser tillbaka på din uppväxttid, dvs fram till idag, vad har din far (styvfar)**

**haft för huvudsakligt yrke eller sysselsättning? )**

**___________________________________________________________________________**

**TILL IVE: Försök ta reda på följande**

**- Huvudsakliga arbetsuppgifter**

**- Bransch**

**- Verksamhet (ex hemtjänst äldreomsorg, bygg inom mur och puts)**

**- Kvalifikationsnivå (ex ledningsarbete, militärt arbete, arbetare och tjänstemän)**

**___________________________________________________________________________**

**ANGE YRKE / SYSSELSÄTTNING I KLARTEXT:**

**_______________________________________________________________________________**

**F34**

**Om du ser tillbaka på din uppväxttid, dvs fram till idag, vad har din mor (styvmor)**

**haft för huvudsakligt yrke eller sysselsättning?**

**__________________________________________________________________________**

**TILL IVE: Om UP:s mor (styvmor) har haft en sysselsättning som inte finns med i listan,**

**t.ex. hemmafru, arbetslös, studerande, sjukskriven, ekonomiskt oberoende m.m.**

**ange 1 och skriv UP:s svar i klartext, hellre än att "tvinga in" svaret i listan.**

**__________________________________________________________________________**

**1 = YRKET / SYSSELSÄTTNINGEN FINNS EJ I LISTAN**

**YRKE / SYSSELSÄTTNING:**

**_______________________________________________________________________________**

**F34TXT**

**( Om du ser tillbaka på din uppväxttid, dvs fram till idag, vad har din mor (styvmor)**

**haft för huvudsakligt yrke eller sysselsättning? )**

**___________________________________________________________________________**

**TILL IVE: Försök ta reda på följande**

**- Huvudsakliga arbetsuppgifter**

**- Bransch**

**- Verksamhet (ex hemtjänst äldreomsorg, bygg inom mur och puts)**

**- Kvalifikationsnivå (ex ledningsarbete, militärt arbete, arbetare och tjänstemän)**

**___________________________________________________________________________**

**ANGE YRKE / SYSSELSÄTTNING I KLARTEXT:**

**_______________________________________________________________________________**

**F41A**

**Hur skulle du vilja beskriva din fars inställning till religion?**

**Skulle beskriva honom som ...**

**1 ... mycket religiös,**

**2 ... ganska religiös,**

**3 ... inte speciellt religiös, eller**

**4 ... inte religiös alls?**

**_______________________________________________________________________________**

**F41B**

**Vilken religion tillhör / tillhörde han?**

**1 Protestantisk kristendom**

**2 Katolsk kristendom**

**3 Ortodox kristendom**

**4 Frikyrklig (inkl mormon, jehovas vittne etc)**

**5 Kristendom, ospecifierat**

**6 Islam, shia (muslim = islam)**

**7 Islam, sunni (muslim = islam)**

**8 Islam - ospecifierat (muslim = islam)**

**9 Judendom**

**10 Annan ...**

**_______________________________________________________________________________**

**F41BTXT**

**( Vilken religion tillhör / tillhörde han? )**

**ANGE ANNAN RELIGION:**

**_______________________________________________________________________________**

**F42A**

**Hur skulle du vilja beskriva din mors inställning till religion?**

**Skulle beskriva henne som ...**

**LÄS UPP SVARSALTERNATIVEN!**

**1 ... mycket religiös,**

**2 ... ganska religiös,**

**3 ... inte speciellt religiös, eller**

**4 ... inte religiös alls?**

**_______________________________________________________________________________**

**F42B**

**Vilken religion tillhör / tillhörde hon?**

**1 Protestantisk kristendom**

**2 Katolsk kristendom**

**3 Ortodox kristendom**

**4 Frikyrklig (inkl mormon, jehovas vittne etc)**

**5 Kristendom, ospecifierat**

**6 Islam, shia (muslim=islam)**

**7 Islam, sunni (muslim=islam)**

**8 Islam - ospecifierat (muslim=islam)**

**9 Judendom**

**10 Annan ...**

**_______________________________________________________________________________**

**F42BTXT**

**( Vilken religion tillhör / tillhörde hon? )**

**ANGE ANNAN RELIGION:**

**_______________________________________________________________________________**

**F51A**

**<<TEXT 'Har du bott i ett annat land än Sverige under minst två år?' om UPUtlandsFodd = 'nej' >><<TEXT 'Sedan du kom till Sverige första gången, har du bott i ett annat land under minst två år?' om UPUtlandsFodd = 'ja' >>**

**1 Ja**

**2 Nej**

**_______________________________________________________________________________**

**TAB1START**

**VISAS EJ PÅ SKÄRMEN!**

**Här börjar tablån: Utlandsvistelser**

**_______________________________________________________________________________**

**F52A**

**<<TEXT 'Om du har bott utomlands i flera länder eller vid flera olika tillfällen, så tar vi en utlandsvistelse ' om TAB1varv = 1 >><<TEXT 'I vilket land bodde du då? ' om TAB1varv > 1 >>**

**<<TEXT 'i taget. Vi börjar med den första utlandsvistelsen ' om TAB1varv = 1 >><<TEXT '... ' om TAB1varv = 1 >><<TEXT '(sedan du kom till Sverige första gången).' om TAB1varv = 1 & UPUtlandsFodd = 'ja' >>**

**<<TEXT 'I vilket land bodde du då? ' om TAB1varv = 1 >>**

**__________________________________________________________________**

**TILL IVE: Ange UP:s spontana svar. Gör inte om svaret för att få träff i listan,**

**utan skriv hellre landet i klartext i nästa fråga.**

**__________________________________________________________________**

**1 = LANDET FINNS INTE I LISTAN**

**LAND:**

**_______________________________________________________________________________**

**F52ATXT**

**( I vilket land bodde du då? )**

**ANGE LAND I KLARTEXT:**

**_______________________________________________________________________________**

**F52B1**

**UTLANDSVISTELSE: <<TEXT TAB1varv >>**

**Vilket år flyttade du dit?**

**__________________________________________________________**

**TILL IVE: Land: <<TEXT LandsNamn >>**

**__________________________________________________________**

**FRÅN OCH MED ÅR:**

**_______________________________________________________________________________**

**F52B2**

**UTLANDSVISTELSE: <<TEXT TAB1varv >>**

**Vilket år flyttade du därifrån?**

**__________________________________________________________**

**TILL IVE: Land: <<TEXT LandsNamn >>**

**__________________________________________________________**

**TILL OCH MED ÅR:**

**_______________________________________________________________________________**

**F53**

**UTLANDSVISTELSE: <<TEXT TAB1varv >>**

**Gick du i skolan där under den perioden?**

**______________________________________________________________________**

**TILL IVE: Period: <<TEXT FromAr >> - <<TEXT TomAr >> i <<TEXT LandsNamn >>**

**______________________________________________________________________**

**1 Ja**

**2 Nej**

**_______________________________________________________________________________**

**F54**

**UTLANDSVISTELSE: <<TEXT TAB1varv >>**

**Arbetade du där under den perioden?**

**______________________________________________________________________**

**TILL IVE: Period: <<TEXT FromAr >> - <<TEXT TomAr >> i <<TEXT LandsNamn >>**

**______________________________________________________________________**

**1 Ja**

**2 Nej**

**_______________________________________________________________________________**

**TAB1EXT**

**Har du sedan dess bott i något annat land än Sverige under minst två år?**

**1 Ja**

**2 Nej**

**_______________________________________________________________________________**

**TAB1SLUT**

**VISAS EJ PÅ SKÄRMEN!**

**Här slutar tablån: Utlandsvistelser**

**_______________________________________________________________________________**

**F55A**

**Har du besökt <<TEXT 'dina föräldrars ' om MorUtlandsFodd = 'ja' & FarUtlandsFodd = 'ja' & ForaldrarSammaFodelseLand = 'ja' >><<TEXT 'din mammas ' om MorUtlandsFodd = 'ja' & (FarUtlandsFodd = 'nej' | ForaldrarSammaFodelseLand = 'nej') >><<TEXT 'och/eller ' om MorUtlandsFodd = 'ja' & FarUtlandsFodd = 'ja' & ForaldrarSammaFodelseLand = 'nej' >><<TEXT 'din pappas ' om FarUtlandsFodd = 'ja' & (MorUtlandsFodd = 'nej' | ForaldrarSammaFodelseLand = 'nej') >>födelseland under de senaste 5 åren?**

**1 Ja**

**2 Nej**

**_______________________________________________________________________________**

**F55B**

**Hur många gånger har du besökt <<TEXT 'dina föräldrars ' om MorUtlandsFodd = 'ja' & FarUtlandsFodd = 'ja' & ForaldrarSammaFodelseLand = 'ja' >><<TEXT 'din mammas ' om MorUtlandsFodd = 'ja' & (FarUtlandsFodd = 'nej' | ForaldrarSammaFodelseLand = 'nej') >><<TEXT 'och/eller ' om MorUtlandsFodd = 'ja' & FarUtlandsFodd = 'ja' & ForaldrarSammaFodelseLand = 'nej' >><<TEXT 'din pappas ' om FarUtlandsFodd = 'ja' & (MorUtlandsFodd = 'nej' | ForaldrarSammaFodelseLand = 'nej') >>födelseland under de senaste 5 åren?**

**ANGE ANTAL GÅNGER:**

**_______________________________________________________________________________**

**F56**

**Hur ofta är du i kontakt med familj eller vänner i <<TEXT 'dina föräldrars ' om MorUtlandsFodd = 'ja' & FarUtlandsFodd = 'ja' & ForaldrarSammaFodelseLand = 'ja' >><<TEXT 'din mammas ' om MorUtlandsFodd = 'ja' & (FarUtlandsFodd = 'nej' | ForaldrarSammaFodelseLand = 'nej') >><<TEXT 'och/eller ' om MorUtlandsFodd = 'ja' & FarUtlandsFodd = 'ja' & ForaldrarSammaFodelseLand = 'nej' >><<TEXT 'din pappas ' om FarUtlandsFodd = 'ja' & (MorUtlandsFodd = 'nej' | ForaldrarSammaFodelseLand = 'nej') >>födelseland?**

**(Det kan vara via post, telefon, email, osv.)**

**LÄS UPP SVARSALTERNATIVEN VID BEHOV!**

**1 Minst varje vecka**

**2 Minst någon gång i månaden**

**3 Minst två gånger om året**

**4 Sällan**

**5 Aldrig**

**_______________________________________________________________________________**

**F57**

**Har du under de senaste 2 åren skickat pengar till någon i <<TEXT 'dina föräldrars ' om MorUtlandsFodd = 'ja' & FarUtlandsFodd = 'ja' & ForaldrarSammaFodelseLand = 'ja' >><<TEXT 'din mammas ' om MorUtlandsFodd = 'ja' & (FarUtlandsFodd = 'nej' | ForaldrarSammaFodelseLand = 'nej') >><<TEXT 'och/eller ' om MorUtlandsFodd = 'ja' & FarUtlandsFodd = 'ja' & ForaldrarSammaFodelseLand = 'nej' >><<TEXT 'din pappas ' om FarUtlandsFodd = 'ja' & (MorUtlandsFodd = 'nej' | ForaldrarSammaFodelseLand = 'nej') >>födelseland?**

**1 Ja**

**2 Nej**

**_______________________________________________________________________________**

**F61**

**Vilket språk brukar du främst tala med dina föräldrar?**

**1 Svenska**

**2 Persiska**

**3 Kurdiska**

**4 Arabiska**

**5 Engelska**

**6 Albanska**

**7 Serbo-kroatiska (inkl. serbiska och kroatiska)**

**8 Bosniska**

**9 Annat språk ...**

**_______________________________________________________________________________**

**F61TXT**

**( Vilket språk brukar du främst tala med dina föräldrar? )**

**ANGE ANNAT SPRÅK:**

**_______________________________________________________________________________**

**F62**

**Brukar du även tala något annat språk med dina föräldrar?**

**1 NEJ**

**<<TEXT ' 2 Svenska ' om F61^=1>>**

**<<TEXT ' 3 Persiska ' om F61^=2>>**

**<<TEXT ' 4 Kurdiska ' om F61^=3>>**

**<<TEXT ' 5 Arabiska ' om F61^=4>>**

**<<TEXT ' 6 Engelska ' om F61^=5>>**

**<<TEXT ' 7 Albanska ' om F61^=6>>**

**<<TEXT ' 8 Serbo-kroatiska (inkl. serbiska och kroatiska) ' om F61^=7>>**

**<<TEXT ' 9 Bosniska ' om F61^=8>>**

**10 Annat språk ...**

**_______________________________________________________________________________**

**F62TXT**

**( Brukar du även tala något annat språk med dina föräldrar? )**

**ANGE ANNAT SPRÅK:**

**_______________________________________________________________________________**

**F63**

**Vilket språk brukar du främst tala med dina vänner?**

**1 Svenska**

**2 Persiska**

**3 Kurdiska**

**4 Arabiska**

**5 Engelska**

**6 Albanska**

**7 Serbo-kroatiska (inkl. serbiska och kroatiska)**

**8 Bosniska**

**9 Annat språk ...**

**_______________________________________________________________________________**

**F63TXT**

**( Vilket språk brukar du främst tala med dina vänner? )**

**ANGE ANNAT SPRÅK:**

**_______________________________________________________________________________**

**F64**

**Brukar du även tala något annat språk med dina vänner?**

**1 NEJ**

**<<TEXT ' 2 Svenska ' om F63^=1>>**

**<<TEXT ' 3 Persiska ' om F63^=2>>**

**<<TEXT ' 4 Kurdiska ' om F63^=3>>**

**<<TEXT ' 5 Arabiska ' om F63^=4>>**

**<<TEXT ' 6 Engelska ' om F63^=5>>**

**<<TEXT ' 7 Albanska ' om F63^=6>>**

**<<TEXT ' 8 Serbo-kroatiska (inkl. serbiska och kroatiska) ' om F63^=7>>**

**<<TEXT ' 9 Bosniska ' om F63^=8>>**

**10 Annat språk ...**

**_______________________________________________________________________________**

**F64TXT**

**( Brukar du även tala något annat språk med dina vänner? )**

**ANGE ANNAT SPRÅK:**

**_______________________________________________________________________________**

**F7INTRO**

**Nu kommer ett avsnitt som handlar om dina vänner och bekanta.**

**Tänk på de fem personer som du träffar och umgås med oftast på din fritid.**

**Innan jag börjar med själva frågorna, behöver jag anteckna deras namn.**

**Nämn bara personer som är äldre än 10 år.**

**Det räcker med förnamn och första bokstaven i efternamnet. Jag behöver**

**namnen bara för att vi båda senare i intervjun ska veta vilken person det är**

**som jag frågar om.**

**__________________________________________________________________**

**TILL IVE: Det handlar om vänner helt enkelt; men här kan också föräldrar,**

**syskon och andra släktingar vara med.**

**Försök få UP att uppge samtliga 5 personer!**

**OBS! Om UP inte kan eller vill uppge någon person alls, tryck F9.**

**__________________________________________________________________**

**TRYCK 1 OCH ENTER FÖR ATT BÖRJA ANTECKNA NAMNEN.**

**_______________________________________________________________________________**

**TAB2START**

**VISAS EJ PÅ SKÄRMEN!**

**Här börjar tablån: Vännernas namn**

**_______________________________________________________________________________**

**F7NAMN**

**PERSON <<TEXT TAB2varv >>**

**<<TEXT 'Ska vi börja med den vän eller person som du träffar mest. Vad heter han eller hon? ' om TAB2varv = 1 >><<TEXT 'Vad heter den andra personen? ' om TAB2varv = 2 >><<TEXT '... och nästa person? ' om TAB2varv > 2 >>**

**___________________________________________________________________**

**TILL IVE: Om UP tycker att det är obehagligt att uppge personens riktiga namn,**

**be om ett "alias" i stället.**

**___________________________________________________________________**

**<<TEXT 'OM INGA FLER PERSONER, ANGE 0. ' om TAB2varv > 1 >>**

**ANGE NAMN (PERSON <<TEXT TAB2varv >>):**

**_______________________________________________________________________________**

**TAB2SLUT**

**VISAS EJ PÅ SKÄRMEN!**

**Här slutar tablån: Vännernas namn**

**_______________________________________________________________________________**

**TAB3START**

**VISAS EJ PÅ SKÄRMEN!**

**Här börjar tablån: Vänner**

**_______________________________________________________________________________**

**F71**

**<<TEXT 'Då börjar jag med några frågor om dina vänner och jag börjar med ' om TAB3varv = 1 >><<TEXT 'Då går vi över till ' om TAB3varv > 1 >><<TEXT VanNamn >>.**

**Är det en man eller en kvinna?**

**___________________________________________________________________**

**TILL IVE: Frågan behöver inte ställas om kön uppenbart framgår av namnet,**

**men bekräfta svaret, så att UP vet vad du registrerar.**

**___________________________________________________________________**

**1 Man**

**2 Kvinna**

**_______________________________________________________________________________**

**F7RELATION**

**PERSON <<TEXT TAB3varv >>: <<TEXT VanNamn >>**

**Är <<TEXT HanHon >> ett syskon, <<TEXT 'pojkvän' om VanKon = '1' >><<TEXT 'flickvän' om VanKon = '2' >><<TEXT 'pojkvän / flickvän' om VanKon = '' >> eller släkt med dig på något annat sätt?**

**1 Förälder**

**2 Syskon**

**3 <<TEXT 'Pojkvän' om VanKon = '1' >><<TEXT 'Flickvän' om VanKon = '2' >><<TEXT 'Pojkvän / Flickvän' om VanKon = '' >>**

**4 <<TEXT 'Make' om VanKon = '1' >><<TEXT 'Maka' om VanKon = '2' >><<TEXT 'Make / Maka' om VanKon = '' >>**

**5 Kusin**

**6 Annan släkt**

**7 Nej, inte släkting eller partner**

**_______________________________________________________________________________**

**F72**

**PERSON <<TEXT TAB3varv >>: <<TEXT VanNamn >>**

**Ungefär hur gammal är <<TEXT HanHon >>?**

**_________________________________________________________________________**

**TILL IVE: Ange svaret i hela år. Be UP att svara så exakt som möjligt.**

**Om UP svarar med intervall, ange medelvärde, t.ex. "30 - 40 år", ange 35.**

**_________________________________________________________________________**

**ÅLDER (ANTAL ÅR):**

**_______________________________________________________________________________**

**F73**

**PERSON <<TEXT TAB3varv >>: <<TEXT VanNamn >>**

**Bor <<TEXT HanHon >> i samma bostadsområde som du?**

**FÖRTYDLIGA VID BEHOV: Med bostadsområde menar vi det område, som du själv**

**betraktar som ditt bostadsområde.**

**1 Ja**

**2 Nej**

**_______________________________________________________________________________**

**F74**

**PERSON <<TEXT TAB3varv >>: <<TEXT VanNamn >>**

**Är <<TEXT HanHon >> född i samma land som du?**

**1 Ja**

**2 Nej**

**_______________________________________________________________________________**

**F75**

**PERSON <<TEXT TAB3varv >>: <<TEXT VanNamn >>**

**I vilket land är <<TEXT HanHon >> född?**

**__________________________________________________________________**

**TILL IVE: Ange UP:s spontana svar. Gör inte om svaret för att få träff i listan,**

**utan skriv hellre landet i klartext i nästa fråga.**

**__________________________________________________________________**

**1 = LANDET FINNS INTE I LISTAN**

**LAND:**

**_______________________________________________________________________________**

**F75TXT**

**PERSON <<TEXT TAB3varv >>: <<TEXT VanNamn >>**

**( I vilket land är <<TEXT HanHon >> född? )**

**ANGE LAND I KLARTEXT:**

**_______________________________________________________________________________**

**F75REGION**

**PERSON <<TEXT TAB3varv >>: <<TEXT VanNamn >>**

**Vet du i vilken region i världen <<TEXT HanHon >> är född?**

**LÄS UPP SVARSALTERNATIVEN VID BEHOV!**

**1 Nej**

**2 Ja, i Norden / Skandinavien**

**3 Ja, i Västeuropa (utom Norden)**

**4 Ja, i Östeuropa (utom Turkiet)**

**5 Ja, i Mellanöstern (inkl Turkiet)**

**6 Ja, i Asien (utom Mellanöstern)**

**7 Ja, i Afrika**

**8 Ja, i Nordamerika**

**9 Ja, i Sydamerika**

**10 Ja, men inget av ovanstående regioner**

**_______________________________________________________________________________**

**F76A**

**PERSON <<TEXT TAB3varv >>: <<TEXT VanNamn >>**

**Är <<TEXT HansHennes >> föräldrar födda i Sverige?**

**1 Ja, båda foräldrar**

**2 Nej, bara mamman (är född i Sverige)**

**3 Nej, bara pappan (är född i Sverige)**

**4 Nej, ingen av dem**

**_______________________________________________________________________________**

**F76B**

**PERSON <<TEXT TAB3varv >>: <<TEXT VanNamn >>**

**I vilket land är <<TEXT HansHennes>> mamma född?**

**__________________________________________________________________**

**TILL IVE: Ange UP:s spontana svar. Gör inte om svaret för att få träff i listan,**

**utan skriv hellre landet i klartext i nästa fråga.**

**__________________________________________________________________**

**1 = LANDET FINNS INTE I LISTAN**

**LAND:**

**_______________________________________________________________________________**

**F76BTXT**

**PERSON <<TEXT TAB3varv >>: <<TEXT VanNamn >>**

**( I vilket land är <<TEXT HansHennes>> mamma född? )**

**ANGE LAND I KLARTEXT:**

**_______________________________________________________________________________**

**F76BREGION**

**PERSON <<TEXT TAB3varv >>: <<TEXT VanNamn >>**

**Vet du i vilken region i världen <<TEXT HansHennes>> mamma är född?**

**LÄS UPP SVARSALTERNATIVEN VID BEHOV!**

**1 Nej**

**2 Ja, i Norden / Skandinavien**

**3 Ja, i Västeuropa (utom Norden)**

**4 Ja, i Östeuropa (utom Turkiet)**

**5 Ja, i Mellanöstern (inkl Turkiet)**

**6 Ja, i Asien (utom Mellanöstern)**

**7 Ja, i Afrika**

**8 Ja, i Nordamerika**

**9 Ja, i Sydamerika**

**10 Ja, men inget av ovanstående regioner**

**_______________________________________________________________________________**

**F76C**

**PERSON <<TEXT TAB3varv >>: <<TEXT VanNamn >>**

**I vilket land är <<TEXT HansHennes>> pappa född?**

**__________________________________________________________________**

**TILL IVE: Ange UP:s spontana svar. Gör inte om svaret för att få träff i listan,**

**utan skriv hellre landet i klartext i nästa fråga.**

**__________________________________________________________________**

**1 = LANDET FINNS INTE I LISTAN**

**LAND:**

**_______________________________________________________________________________**

**F76CTXT**

**PERSON <<TEXT TAB3varv >>: <<TEXT VanNamn >>**

**( I vilket land är <<TEXT HansHennes>> pappa född? )**

**ANGE LAND I KLARTEXT:**

**_______________________________________________________________________________**

**F76CREGION**

**PERSON <<TEXT TAB3varv >>: <<TEXT VanNamn >>**

**Vet du i vilken region i världen <<TEXT HansHennes>> pappa är född?**

**LÄS UPP SVARSALTERNATIVEN VID BEHOV!**

**1 Nej**

**2 Ja, i Norden / Skandinavien**

**3 Ja, i Västeuropa (utom Norden)**

**4 Ja, i Östeuropa (utom Turkiet)**

**5 Ja, i Mellanöstern (inkl Turkiet)**

**6 Ja, i Asien (utom Mellanöstern)**

**7 Ja, i Afrika**

**8 Ja, i Nordamerika**

**9 Ja, i Sydamerika**

**10 Ja, men inget av ovanstående regioner**

**_______________________________________________________________________________**

**F78D**

**PERSON <<TEXT TAB3varv >>: <<TEXT VanNamn >>**

**Gick <<TEXT VanNamn >> och du i samma grundskola eller gymnasieskola?**

**_______________________________________________________________**

**TILL IVE: Svara JA även om UP och <<TEXT VanNamn >> gått i samma skola**

**bara en kort tid.**

**_______________________________________________________________**

**1 Ja**

**2 Nej**

**_______________________________________________________________________________**

**F78DD**

**PERSON <<TEXT TAB3varv >>: <<TEXT VanNamn >>**

**Gick ni i samma klass?**

**_______________________________________________________________**

**TILL IVE: Svara JA även om UP och <<TEXT VanNamn >> gått i samma klass**

**bara en kort tid.**

**_______________________________________________________________**

**1 Ja**

**2 Nej**

**_______________________________________________________________________________**

**F78F**

**PERSON <<TEXT TAB3varv >>: <<TEXT VanNamn >>**

**Är ni, eller har ni varit, förenings- eller klubbkamrater?**

**1 Ja, vi är förenings-/klubbkamrater**

**2 Ja, vi har varit förenings-/klubbkamrater**

**3 Nej**

**_______________________________________________________________________________**

**F78HS1**

**VISAS EJ PÅ SKÄRMEN!**

**Här lagras svar från F78H:**

**1 Ja, vi är studiekamrater på universitet / högskola**

**_______________________________________________________________________________**

**F78HS2**

**VISAS EJ PÅ SKÄRMEN!**

**Här lagras svar från F78H:**

**2 Ja, vi har varit studiekamrater på universitet / högskola**

**_______________________________________________________________________________**

**F78H**

**PERSON <<TEXT TAB3varv >>: <<TEXT VanNamn >>**

**Är ni, eller har ni varit, studiekamrater på universitet / högskola?**

**FLERA SVAR KAN ANGES. TRYCK MELLANSLAG MELLAN SVAREN.**

**1 Ja, vi är studiekamrater på universitet / högskola**

**2 Ja, vi har varit studiekamrater på universitet / högskola**

**3 Nej**

**_______________________________________________________________________________**

**F78HHS1**

**VISAS EJ PÅ SKÄRMEN!**

**Här lagras svar från F78H:**

**1 Ja, vi är arbetskamrater**

**_______________________________________________________________________________**

**F78HHS2**

**VISAS EJ PÅ SKÄRMEN!**

**Här lagras svar från F78H:**

**2 Ja, vi har varit arbetskamrater**

**_______________________________________________________________________________**

**F78HH**

**PERSON <<TEXT TAB3varv >>: <<TEXT VanNamn >>**

**Är ni, eller har ni varit, arbetskamrater?**

**FLERA SVAR KAN ANGES. TRYCK MELLANSLAG MELLAN SVAREN.**

**1 Ja, vi är arbetskamrater**

**2 Ja, vi har varit arbetskamrater**

**3 Nej**

**_______________________________________________________________________________**

**F79A**

**PERSON <<TEXT TAB3varv >>: <<TEXT VanNamn >>**

**Hur ofta brukar du träffa <<TEXT VanNamn >>?**

**::**

**_______________________________________________________________________________**

**TILL IVE: "Träffa" innebär att träffas FYSISKT, dvs att man ses på samma plats samtidigt**

**(till skillnad från telefonsamtal eller virtuell kontakt via telefon / chat / sms eller e-mail).**

**Med 'brukar' avses 'i normala fall', dvs en normal vecka / månad.**

**_______________________________________________________________________________**

**1 Dagligen**

**2 Flera gånger i veckan**

**3 Någon gång i veckan**

**4 Någon gång i månaden**

**5 Några gånger om året**

**6 Sällan eller aldrig**

**_______________________________________________________________________________**

**F79B**

**PERSON <<TEXT TAB3varv >>: <<TEXT VanNamn >>**

**Hur ofta brukar du vara i kontakt med <<TEXT HonomHenne >> via telefon, internet, e-post eller sms?**

**::**

**_____________________________________________________________________**

**TILL IVE: "Att vara i kontakt med" avser: telefonsamtal eller s.k. virtuell kontakt**

**via chat / sms eller e-mail.**

**Med 'brukar' avses 'i normala fall', dvs en normal vecka / månad.**

**_____________________________________________________________________**

**1 Dagligen**

**2 Flera gånger i veckan**

**3 Någon gång i veckan**

**4 Någon gång i månaden**

**5 Några gånger om året**

**6 Sällan eller aldrig**

**_______________________________________________________________________________**

**F79DS1**

**VISAS EJ PÅ SKÄRMEN!**

**Här lagras svar från F79D:**

**1 Politik och samhällsfrågor**

**_______________________________________________________________________________**

**F79DS2**

**VISAS EJ PÅ SKÄRMEN!**

**Här lagras svar från F79D:**

**2 Kultur**

**_______________________________________________________________________________**

**F79DS3**

**VISAS EJ PÅ SKÄRMEN!**

**Här lagras svar från F79D:**

**3 Litteratur**

**_______________________________________________________________________________**

**F79DS4**

**VISAS EJ PÅ SKÄRMEN!**

**Här lagras svar från F79D:**

**4 Idrott**

**_______________________________________________________________________________**

**F79DS5**

**VISAS EJ PÅ SKÄRMEN!**

**Här lagras svar från F79D:**

**5 Religion**

**_______________________________________________________________________________**

**F79DS6**

**VISAS EJ PÅ SKÄRMEN!**

**Här lagras svar från F79D:**

**6 Ekonomi**

**_______________________________________________________________________________**

**F79DS7**

**VISAS EJ PÅ SKÄRMEN!**

**Här lagras svar från F79D:**

**7 Sex**

**_______________________________________________________________________________**

**F79D**

**PERSON <<TEXT TAB3varv >>: <<TEXT VanNamn >>**

**Brukar du diskutera något av följande med <<TEXT HonomHenne >>?**

**FLERA SVAR KAN ANGES. TRYCK MELLANSLAG MELLAN SVAREN.**

**LÄS UPP SVARSALTERNATIVEN, (ETT I TAGET) !**

**1 Politik och samhällsfrågor**

**2 Kultur**

**3 Litteratur**

**4 Idrott**

**5 Religion**

**6 Ekonomi**

**7 Sex**

**8 NEJ, INGET AV OVANSTÅENDE**

**_______________________________________________________________________________**

**F79E**

**PERSON <<TEXT TAB3varv >>: <<TEXT VanNamn >>**

**Vilket språk brukar du främst tala med <<TEXT VanNamn >>?**

**1 Svenska**

**2 Persiska**

**3 Kurdiska**

**4 Arabiska**

**5 Engelska**

**6 Albanska**

**7 Serbo-kroatiska (inkl. serbiska och kroatiska)**

**8 Bosniska**

**9 Annat språk ...**

**_______________________________________________________________________________**

**F79ETXT**

**PERSON <<TEXT TAB3varv >>: <<TEXT VanNamn >>**

**( Vilket språk brukar du främst tala med <<TEXT VanNamn >>? )**

**ANGE ANNAT SPRÅK:**

**_______________________________________________________________________________**

**F710**

**PERSON <<TEXT TAB3varv >>: <<TEXT VanNamn >>**

**Hur många år har du känt <<TEXT HonomHenne >>?**

**_____________________________________________________________________**

**TILL IVE: Om UP inte vet, be UP uppskatta.**

**Om mindre än 1 år, ange 0.**

**Om UP svarar med ett intervall, beräkna ett medelvärde och avrunda nedåt.**

**Till exempel "10 - 15 år", ange 12.**

**_____________________________________________________________________**

**ANTAL ÅR:**

**_______________________________________________________________________________**

**F711**

**PERSON <<TEXT TAB3varv >>: <<TEXT VanNamn >>**

**Var träffas ni för det mesta?**

**LÄS UPP SVARSALTERNATIVEN VID BEHOV!**

**1 På universitetet / högskolan / skolan**

**2 I en förening**

**3 På arbetet**

**4 Vid familjesammankomster**

**5 Hemma hos någon av er**

**6 Hemma hos andra kompisar**

**7 Ute på stan**

**8 På något internet-community (t.ex. Facebook)**

**9 På semesterresan / sommarstället**

**10 ANNAT ...**

**_______________________________________________________________________________**

**F711TXT**

**PERSON <<TEXT TAB3varv >>: <<TEXT VanNamn >>**

**( Var träffas ni för det mesta? )**

**ANGE ANNAN PLATS:**

**_______________________________________________________________________________**

**F712**

**PERSON <<TEXT TAB3varv >>: <<TEXT VanNamn >>**

**Hur bra upplever du att er relation är?**

**Svara på en skala från 1 till 5, där 1 är inte alls bra och 5 är väldigt bra.**

**1 2 3 4 5**

**|---------------|---------------|---------------|---------------|**

**inte alls bra väldigt bra**

**_______________________________________________________________________________**

**F713A**

**PERSON <<TEXT TAB3varv >>: <<TEXT VanNamn >>**

**Hur mycket litar du på <<TEXT VanNamn >>?**

**Svara på en skala från 1 till 5, där 1 är inte alls och 5 är väldigt mycket.**

**1 2 3 4 5**

**|---------------|---------------|---------------|---------------|**

**inte alls väldigt mycket**

**_______________________________________________________________________________**

**F713B**

**PERSON <<TEXT TAB3varv >>: <<TEXT VanNamn >>**

**Är det här en person som du skulle kunna diskutera ett viktigt personligt problem med?**

**____________________________________________________________________**

**TILL IVE: Personligt problem kan handla om allt från kärlek, ohälsa, studier**

**till kriminalitet och missbruk.**

**____________________________________________________________________**

**1 Ja**

**2 Nej**

**_______________________________________________________________________________**

**F713CS1**

**VISAS EJ PÅ SKÄRMEN!**

**Här lagras svar från F713C;**

**1 Nickar mot varandra**

**_______________________________________________________________________________**

**F713CS2**

**VISAS EJ PÅ SKÄRMEN!**

**Här lagras svar från F713C;**

**2 Säger "Hej!" (eller annan hälsningsfras)**

**_______________________________________________________________________________**

**F713CS3**

**VISAS EJ PÅ SKÄRMEN!**

**Här lagras svar från F713C;**

**3 Skakar hand**

**_______________________________________________________________________________**

**F713CS4**

**VISAS EJ PÅ SKÄRMEN!**

**Här lagras svar från F713C;**

**4 Dunkar varandra i ryggen**

**_______________________________________________________________________________**

**F713CS5**

**VISAS EJ PÅ SKÄRMEN!**

**Här lagras svar från F713C;**

**5 Kramar om varandra**

**_______________________________________________________________________________**

**F713CS6**

**VISAS EJ PÅ SKÄRMEN!**

**Här lagras svar från F713C;**

**6 Puss på kinden**

**_______________________________________________________________________________**

**F713CS7**

**VISAS EJ PÅ SKÄRMEN!**

**Här lagras svar från F713C;**

**7 Puss på munnen**

**_______________________________________________________________________________**

**F713CS8**

**VISAS EJ PÅ SKÄRMEN!**

**Här lagras svar från F713C;**

**8 På annat sätt**

**_______________________________________________________________________________**

**F713C**

**PERSON <<TEXT TAB3varv >>: <<TEXT VanNamn >>**

**Hur brukar du hälsa på <<TEXT VanNamn >> om ni stöter ihop på stan eller på gatan?**

**FLERA SVAR KAN ANGES. TRYCK MELLANSLAG MELLAN SVAREN.**

**<<TEXT 'LÄS UPP SVARSALTERNATIVEN! ' om TAB3varv = 1 >><<TEXT 'LÄS UPP SVARSALTERNATIVEN VID BEHOV! ' om TAB3varv > 1 >>**

**1 Nickar mot varandra**

**2 Säger "Hej!" (eller annan hälsningsfras)**

**3 Skakar hand**

**4 Dunkar varandra i ryggen**

**5 Kramar om varandra**

**6 Puss på kinden**

**7 Puss på munnen**

**8 På annat sätt**

**_______________________________________________________________________________**

**F714**

**PERSON <<TEXT TAB3varv >>: <<TEXT VanNamn >>**

**Vilken är <<TEXT HansHennes >> högsta påbörjade utbildning?**

**1 Grundskola**

**2 Yrkesutbildning**

**3 Gymnasium**

**4 Högskola / Universitet**

**_______________________________________________________________________________**

**F715**

**PERSON <<TEXT TAB3varv >>: <<TEXT VanNamn >>**

**Arbetar eller studerar <<TEXT VanNamn >>, eller är <<TEXT HanHon >> arbetslös?**

**1 Ja, arbetar**

**2 Ja, studerar**

**3 Ja, både arbetar och studerar**

**4 Ja, arbetslös**

**5 Nej**

**_______________________________________________________________________________**

**F716**

**PERSON <<TEXT TAB3varv >>: <<TEXT VanNamn >>**

**Vilket yrke har <<TEXT HanHon >>?**

**1 = YRKET FINNS EJ I LISTAN**

**YRKE:**

**_______________________________________________________________________________**

**F716TXT**

**PERSON <<TEXT TAB3varv >>: <<TEXT VanNamn >>**

**( Vilket yrke har <<TEXT HanHon >>? )**

**___________________________________________________________________________**

**TILL IVE: Försök ta reda på följande**

**- Huvudsakliga arbetsuppgifter**

**- Bransch**

**- Verksamhet (ex hemtjänst äldreomsorg, bygg inom mur och puts)**

**- Kvalifikationsnivå (ex ledningsarbete, militärt arbete, arbetare och tjänstemän)**

**___________________________________________________________________________**

**ANGE YRKET I KLARTEXT:**

**_______________________________________________________________________________**

**F718A**

**PERSON <<TEXT TAB3varv >>: <<TEXT VanNamn >>**

**Röker <<TEXT VanNamn >>?**

**1 Ja**

**2 Nej**

**_______________________________________________________________________________**

**F718AA**

**PERSON <<TEXT TAB3varv >>: <<TEXT VanNamn >>**

**Hur mycket?**

**Feströker <<TEXT HanHon >> eller röker <<TEXT HanHon >> mindre än ett paket om dagen**

**eller mer än ett paket om dagen?**

**1 Feströker**

**2 Röker mindre än ett paket om dagen**

**3 Röker ett paket om dagen eller mer**

**_______________________________________________________________________________**

**F718B**

**PERSON <<TEXT TAB3varv >>: <<TEXT VanNamn >>**

**Dricker <<TEXT VanNamn >> alkohol?**

**1 Ja**

**2 Nej**

**_______________________________________________________________________________**

**F718BB**

**PERSON <<TEXT TAB3varv >>: <<TEXT VanNamn >>**

**Ungefär hur ofta dricker <<TEXT HanHon >> så mycket alkohol att <<TEXT HanHon >> blir berusad?**

**LÄS UPP SVARSALTERNATIVEN VID BEHOV!**

**1 Tre gånger i veckan eller oftare**

**2 En till två gånger per vecka**

**3 Två till tre gånger per månad**

**4 En gång per månad**

**5 Mer sällan**

**6 Aldrig**

**_______________________________________________________________________________**

**F719A**

**PERSON <<TEXT TAB3varv >>: <<TEXT VanNamn >>**

**Motionerar eller sportar <<TEXT VanNamn >>?**

**Det ska vara minst en halvtimmas sammanhängande motion / sport vid varje tillfälle.**

**::**

**1 Ja**

**2 Nej**

**_______________________________________________________________________________**

**F719B**

**PERSON <<TEXT TAB3varv >>: <<TEXT VanNamn >>**

**Skulle du säga att <<TEXT HanHon >> tränar mer än du, ungefär lika mycket som du eller mindre än du?**

**::**

**1 Mer**

**2 Lika mycket**

**3 Mindre**

**_______________________________________________________________________________**

**F719C**

**PERSON <<TEXT TAB3varv >>: <<TEXT VanNamn >>**

**Brukar <<TEXT HanHon >> äta nyttig och hälsosam mat?**

**1 Ja**

**2 Nej**

**_______________________________________________________________________________**

**F719D**

**PERSON <<TEXT TAB3varv >>: <<TEXT VanNamn >>**

**Är <<TEXT VanNamn >> en person som gillar att ta risker eller som försöker undvika risker?**

**Svara på en skala mellan 1 och 10, där 1 står för mycket försiktig och 10 mycket riskvillig.**

**1 2 3 4 5 6 7 8 9 10**

**|----------|----------|----------|----------|----------|----------|----------|----------|----------|**

**mycket mycket**

**försiktig riskvillig**

**_______________________________________________________________________________**

**F719E**

**PERSON <<TEXT TAB3varv >>: <<TEXT VanNamn >>**

**Hur skulle du beskriva <<TEXT HansHennes >> kroppsbyggnad?**

**Som underviktig, normalviktig, överviktig eller mycket överviktig?**

**1 Underviktig**

**2 Normalviktig**

**3 Överviktig**

**4 Mycket överviktig**

**_______________________________________________________________________________**

**F719F**

**PERSON <<TEXT TAB3varv >>: <<TEXT VanNamn >>**

**Har <<TEXT HanHon>> utsatts för något brott under de senaste 12 månaderna?**

**1 Ja**

**2 Nej**

**_______________________________________________________________________________**

**F720A**

**PERSON <<TEXT TAB3varv >>: <<TEXT VanNamn >>**

**Är <<TEXT VanNamn>> mycket religiös , ganska religiös, inte speciellt religiös eller inte alls religiös?**

**1 Mycket religiös**

**2 Ganska religiös**

**3 Inte speciellt religiös**

**4 Inte alls religiös**

**_______________________________________________________________________________**

**F720B**

**PERSON <<TEXT TAB3varv >>: <<TEXT VanNamn >>**

**Vilken religion tillhör <<TEXT HanHon >>?**

**1 Protestantisk kristendom**

**2 Katolsk kristendom**

**3 Ortodox kristendom**

**4 Frikyrklig (inkl mormon, jehovas vittne etc)**

**5 Kristendom, ospecifierat**

**6 Islam, shia (muslim = islam)**

**7 Islam, sunni (muslim = islam)**

**8 Islam - ospecifierat (muslim = islam)**

**9 Judendom**

**10 Annan ...**

**_______________________________________________________________________________**

**F720BTXT**

**PERSON <<TEXT TAB3varv >>: <<TEXT VanNamn >>**

**( Vilken religion tillhör <<TEXT HanHon >>? )**

**ANGE ANNAN RELIGION:**

**_______________________________________________________________________________**

**TAB3SLUT**

**VISAS EJ PÅ SKÄRMEN!**

**Här slutar tablån: Vänner**

**_______________________________________________________________________________**

**F721V12A**

**Jag skulle också vilja veta om de här personerna, som du har nämnt känner varandra.**

**Känner <<TEXT Van1Namn >> och <<TEXT Van2Namn >> varandra?**

**1 Ja**

**2 Nej**

**_______________________________________________________________________________**

**F721V12B**

**FRÅGAN AVSER: <<TEXT Van1Namn >> och <<TEXT Van2Namn >>**

**Hur väl känner de varandra?**

**LÄS UPP SVARSALTERNATIVEN!**

**1 Inte så väl**

**2 Ganska väl**

**3 Mycket väl**

**_______________________________________________________________________________**

**F721V12C**

**FRÅGAN AVSER: <<TEXT Van1Namn >> och <<TEXT Van2Namn >>**

**Hur väl kommer de överens?**

**Svara på en skala 1 till 5 där 1 är inte alls bra och 5 är väldigt bra.**

**1 2 3 4 5**

**|---------------|---------------|---------------|---------------|**

**inte alls bra väldigt bra**

**_______________________________________________________________________________**

**F721V13A**

**Jag skulle också vilja veta om de här personerna, som du har nämnt känner varandra.**

**Känner <<TEXT Van1Namn >> och <<TEXT Van3Namn >> varandra?**

**1 Ja**

**2 Nej**

**_______________________________________________________________________________**

**F721V13B**

**FRÅGAN AVSER: <<TEXT Van1Namn >> och <<TEXT Van3Namn >>**

**Hur väl känner de varandra?**

**LÄS UPP SVARSALTERNATIVEN!**

**1 Inte så väl**

**2 Ganska väl**

**3 Mycket väl**

**_______________________________________________________________________________**

**F721V13C**

**FRÅGAN AVSER: <<TEXT Van1Namn >> och <<TEXT Van3Namn >>**

**Hur väl kommer de överens?**

**Svara på en skala 1 till 5 där 1 är inte alls bra och 5 är väldigt bra.**

**1 2 3 4 5**

**|---------------|---------------|---------------|---------------|**

**inte alls bra väldigt bra**

**_______________________________________________________________________________**

**F721V23A**

**Känner <<TEXT Van2Namn >> och <<TEXT Van3Namn >> varandra?**

**1 Ja**

**2 Nej**

**_______________________________________________________________________________**

**F721V23B**

**FRÅGAN AVSER: <<TEXT Van2Namn >> och <<TEXT Van3Namn >>**

**Hur väl känner de varandra?**

**LÄS UPP SVARSALTERNATIVEN!**

**1 Inte så väl**

**2 Ganska väl**

**3 Mycket väl**

**_______________________________________________________________________________**

**F721V23C**

**FRÅGAN AVSER: <<TEXT Van2Namn >> och <<TEXT Van3Namn >>**

**Hur väl kommer de överens?**

**Svara på en skala 1 till 5 där 1 är inte alls bra och 5 är väldigt bra.**

**1 2 3 4 5**

**|---------------|---------------|---------------|---------------|**

**inte alls bra väldigt bra**

**_______________________________________________________________________________**

**F721V14A**

**Känner <<TEXT Van1Namn >> och <<TEXT Van4Namn >> varandra?**

**1 Ja**

**2 Nej**

**_______________________________________________________________________________**

**F721V14B**

**FRÅGAN AVSER: <<TEXT Van1Namn >> och <<TEXT Van4Namn >>**

**Hur väl känner de varandra?**

**LÄS UPP SVARSALTERNATIVEN!**

**1 Inte så väl**

**2 Ganska väl**

**3 Mycket väl**

**_______________________________________________________________________________**

**F721V14C**

**FRÅGAN AVSER: <<TEXT Van1Namn >> och <<TEXT Van4Namn >>**

**Hur väl kommer de överens?**

**Svara på en skala 1 till 5 där 1 är inte alls bra och 5 är väldigt bra.**

**1 2 3 4 5**

**|---------------|---------------|---------------|---------------|**

**inte alls bra väldigt bra**

**_______________________________________________________________________________**

**F721V24A**

**Känner <<TEXT Van2Namn >> och <<TEXT Van4Namn >> varandra?**

**1 Ja**

**2 Nej**

**_______________________________________________________________________________**

**F721V24B**

**FRÅGAN AVSER: <<TEXT Van2Namn >> och <<TEXT Van4Namn >>**

**Hur väl känner de varandra?**

**LÄS UPP SVARSALTERNATIVEN!**

**1 Inte så väl**

**2 Ganska väl**

**3 Mycket väl**

**_______________________________________________________________________________**

**F721V24C**

**FRÅGAN AVSER: <<TEXT Van2Namn >> och <<TEXT Van4Namn >>**

**Hur väl kommer de överens?**

**Svara på en skala 1 till 5 där 1 är inte alls bra och 5 är väldigt bra.**

**1 2 3 4 5**

**|---------------|---------------|---------------|---------------|**

**inte alls bra väldigt bra**

**_______________________________________________________________________________**

**F721V34A**

**Känner <<TEXT Van3Namn >> och <<TEXT Van4Namn >> varandra?**

**1 Ja**

**2 Nej**

**_______________________________________________________________________________**

**F721V34B**

**FRÅGAN AVSER: <<TEXT Van3Namn >> och <<TEXT Van4Namn >>**

**Hur väl känner de varandra?**

**LÄS UPP SVARSALTERNATIVEN!**

**1 Inte så väl**

**2 Ganska väl**

**3 Mycket väl**

**_______________________________________________________________________________**

**F721V34C**

**FRÅGAN AVSER: <<TEXT Van3Namn >> och <<TEXT Van4Namn >>**

**Hur väl kommer de överens?**

**Svara på en skala 1 till 5 där 1 är inte alls bra och 5 är väldigt bra.**

**1 2 3 4 5**

**|---------------|---------------|---------------|---------------|**

**inte alls bra väldigt bra**

**_______________________________________________________________________________**

**F721V15A**

**Känner <<TEXT Van1Namn >> och <<TEXT Van5Namn >> varandra?**

**1 Ja**

**2 Nej**

**_______________________________________________________________________________**

**F721V15B**

**FRÅGAN AVSER: <<TEXT Van1Namn >> och <<TEXT Van5Namn >>**

**Hur väl känner de varandra?**

**LÄS UPP SVARSALTERNATIVEN!**

**1 Inte så väl**

**2 Ganska väl**

**3 Mycket väl**

**_______________________________________________________________________________**

**F721V15C**

**FRÅGAN AVSER: <<TEXT Van1Namn >> och <<TEXT Van5Namn >>**

**Hur väl kommer de överens?**

**Svara på en skala 1 till 5 där 1 är inte alls bra och 5 är väldigt bra.**

**1 2 3 4 5**

**|---------------|---------------|---------------|---------------|**

**inte alls bra väldigt bra**

**_______________________________________________________________________________**

**F721V25A**

**Känner <<TEXT Van2Namn >> och <<TEXT Van5Namn >> varandra?**

**1 Ja**

**2 Nej**

**_______________________________________________________________________________**

**F721V25B**

**FRÅGAN AVSER: <<TEXT Van2Namn >> och <<TEXT Van5Namn >>**

**Hur väl känner de varandra?**

**LÄS UPP SVARSALTERNATIVEN!**

**1 Inte så väl**

**2 Ganska väl**

**3 Mycket väl**

**_______________________________________________________________________________**

**F721V25C**

**FRÅGAN AVSER: <<TEXT Van2Namn >> och <<TEXT Van5Namn >>**

**Hur väl kommer de överens?**

**Svara på en skala 1 till 5 där 1 är inte alls bra och 5 är väldigt bra.**

**1 2 3 4 5**

**|---------------|---------------|---------------|---------------|**

**inte alls bra väldigt bra**

**_______________________________________________________________________________**

**F721V35A**

**Känner <<TEXT Van3Namn >> och <<TEXT Van5Namn >> varandra?**

**1 Ja**

**2 Nej**

**_______________________________________________________________________________**

**F721V35B**

**FRÅGAN AVSER: <<TEXT Van3Namn >> och <<TEXT Van5Namn >>**

**Hur väl känner de varandra?**

**LÄS UPP SVARSALTERNATIVEN!**

**1 Inte så väl**

**2 Ganska väl**

**3 Mycket väl**

**_______________________________________________________________________________**

**F721V35C**

**FRÅGAN AVSER: <<TEXT Van3Namn >> och <<TEXT Van5Namn >>**

**Hur väl kommer de överens?**

**Svara på en skala 1 till 5 där 1 är inte alls bra och 5 är väldigt bra.**

**1 2 3 4 5**

**|---------------|---------------|---------------|---------------|**

**inte alls bra väldigt bra**

**_______________________________________________________________________________**

**F721V45A**

**Känner <<TEXT Van4Namn >> och <<TEXT Van5Namn >> varandra?**

**1 Ja**

**2 Nej**

**_______________________________________________________________________________**

**F721V45B**

**FRÅGAN AVSER: <<TEXT Van4Namn >> och <<TEXT Van5Namn >>**

**Hur väl känner de varandra?**

**LÄS UPP SVARSALTERNATIVEN!**

**1 Inte så väl**

**2 Ganska väl**

**3 Mycket väl**

**_______________________________________________________________________________**

**F721V45C**

**FRÅGAN AVSER: <<TEXT Van4Namn >> och <<TEXT Van5Namn >>**

**Hur väl kommer de överens?**

**Svara på en skala 1 till 5, där 1 är inte alls bra och 5 är väldigt bra.**

**1 2 3 4 5**

**|---------------|---------------|---------------|---------------|**

**inte alls bra väldigt bra**

**_______________________________________________________________________________**

**F722INTRO**

**Jag kommer nu att ställa några få frågor om de personer som du nämnde**

**när vi talades vid för tre år sedan.**

**Då nämnde du följande personer ...**

**LÄS UPP NAMNEN!**

**NAMN: <<TEXT OldVan1Namn >>**

**<<TEXT OldVan2Namn >>**

**<<TEXT OldVan3Namn >>**

**<<TEXT OldVan4Namn >>**

**<<TEXT OldVan5Namn >>**

**TRYCK 1 OCH ENTER FÖR ATT FORTSÄTTA.**

**_______________________________________________________________________________**

**TAB4START**

**VISAS EJ PÅ SKÄRMEN!**

**Här börjar tablån: Gamla vänner**

**_______________________________________________________________________________**

**F722A**

**<<TEXT 'Vi börjar med ' om TAB4varv = 1 & AntalVanner > 0 >><<TEXT 'Då fortsätter vi med ' om TAB4varv > 1 & AntalVanner > 0 >><<TEXT OldVanNamn om AntalVanner > 0 >>**

**Är <<TEXT OldVanNamn >> identisk med någon av de <<TEXT AntalVanner >> personer som du har nämnt tidigare i intervjun?**

**___________________________________________________________________**

**TILL IVE: Om UP har angett ett alias eller bara en initial, och inte kommer ihåg**

**vilken person det avser, ange F8 = VET EJ.**

**___________________________________________________________________**

**0 NEJ**

**<<TEXT '1 Ja, identisk med ' om AntalVanner >= 1 >><<TEXT Van1Namn >>**

**<<TEXT '2 Ja, identisk med ' om AntalVanner >= 2 >><<TEXT Van2Namn >>**

**<<TEXT '3 Ja, identisk med ' om AntalVanner >= 3 >><<TEXT Van3Namn >>**

**<<TEXT '4 Ja, identisk med ' om AntalVanner >= 4 >><<TEXT Van4Namn >>**

**<<TEXT '5 Ja, identisk med ' om AntalVanner >= 5 >><<TEXT Van5Namn >>**

**_______________________________________________________________________________**

**F722B**

**<<TEXT 'PERSON ' om AntalVanner > 0 >><<TEXT TAB4varv om AntalVanner > 0 >><<TEXT ' (FRÅN VÅG 1): ' om AntalVanner > 0 >><<TEXT OldVanNamn om AntalVanner > 0 >><<TEXT 'Vi börjar med ' om TAB4varv = 1 & AntalVanner = 0 >><<TEXT 'Då fortsätter vi med ' om TAB4varv > 1 & AntalVanner = 0 >><<TEXT OldVanNamn om AntalVanner = 0 >>**

**Har du fortfarande kontakt med honom/henne?**

**<<TEXT '_______________________________________________________________________________ ' om AntalVanner = 0 >><<TEXT '1 Ja, ofta ' om AntalVanner > 0 >>**

**<<TEXT 'TILL IVE: Om UP har angett ett alias eller bara en initial, och inte kommer ihåg ' om AntalVanner = 0 >><<TEXT '2 Ja, ibland ' om AntalVanner > 0 >>**

**vilken person det avser, ange F8 (VET EJ) på denna och de två kommande frågorna. ' om AntalVanner = 0 >>**

**<<TEXT '_______________________________________________________________________________ ' om AntalVanner = 0 >><<TEXT '3 Nej ' om AntalVanner > 0 >>**

**<<TEXT '1 Ja, ofta ' om AntalVanner = 0 >>**

**<<TEXT '2 Ja, ibland ' om AntalVanner = 0 >>**

**<<TEXT '3 Nej ' om AntalVanner = 0 >>**

**_______________________________________________________________________________**

**F722C**

**PERSON <<TEXT TAB4varv >> (FRÅN VÅG 1): <<TEXT OldVanNamn >>**

**Hur bra upplever du att er relation är?**

**Svara på en skala 1 till 5, där 1 är inte alls bra och 5 är väldigt bra.**

**1 2 3 4 5**

**|---------------|---------------|---------------|---------------|**

**inte alls bra väldigt bra**

**_______________________________________________________________________________**

**F722D**

**PERSON <<TEXT TAB4varv >> (FRÅN VÅG 1): <<TEXT OldVanNamn >>**

**Vet du vad han/hon gör idag?**

**Arbetar eller studerar han/hon, eller är han/hon arbetslös?**

**1 Ja, arbetar**

**2 Ja, studerar**

**3 Ja, både arbetar och studerar**

**4 Ja, arbetslös**

**5 Nej, inget av ovanstående**

**_______________________________________________________________________________**

**TAB4SLUT**

**VISAS EJ PÅ SKÄRMEN!**

**Här slutar tablån: Gamla vänner**

**_______________________________________________________________________________**

**F723**

**Hur många vänner har du (allt som allt)?**

**ANTAL VÄNNER:**

**_______________________________________________________________________________**

**F724A**

**Hur viktiga skulle du säga att sociala medier är för din kontakt med vänner och bekanta?**

**~~~~~~~~~~~**

**Svara på en skala 1 till 5, där 1 är inte alls viktigt och 5 är väldigt viktigt.**

**1 2 3 4 5**

**|---------------|---------------|---------------|---------------|**

**inte alls väldigt**

**viktigt viktigt**

**_______________________________________________________________________________**

**F724B**

**Hur viktig skulle du säga att telefonen är för din kontakt med vänner och bekanta?**

**~~~~~~~**

**Svara på en skala 1 till 5, där 1 är inte alls viktigt och 5 är väldigt viktigt.**

**1 2 3 4 5**

**|---------------|---------------|---------------|---------------|**

**inte alls väldigt**

**viktigt viktigt**

**_______________________________________________________________________________**

**F724C**

**Hur viktiga skulle du säga att face-to-face möten är för din kontakt med vänner och bekanta?**

**~~~~~~~~~~~~~~~**

**Svara på en skala 1 till 5, där 1 är inte alls viktigt och 5 är väldigt viktigt.**

**_______________________________________________________________________________**

**TILL IVE: "Face-to-face" innebär att träffas FYSISKT, dvs att man ses på samma plats samtidigt**

**(till skillnad från t.ex. videosamtal och Skype).**

**_______________________________________________________________________________**

**1 2 3 4 5**

**|---------------|---------------|---------------|---------------|**

**inte alls väldigt**

**viktigt viktigt**

**_______________________________________________________________________________**

**NY1A**

**Nu kommer jag att läsa upp en lista med yrken och be dig säga om någon vän,**

**bekant, familjemedlem, flickvän / pojkvän eller släkting har det yrket.**

**YRKE: Läkare**

**Har någon vän, bekant, familjemedlem, flickvän / pojkvän eller släkting det yrket?**

**1 Ja**

**2 Nej**

**_______________________________________________________________________________**

**NY1B**

**( YRKE: Läkare )**

**Bor den eller de personer i Sverige eller i annat land (eller både och)?**

**1 I Sverige**

**2 I annat land**

**3 Både i Sverige och i annat land**

**_______________________________________________________________________________**

**NY2A**

**YRKE: Kock**

**Har någon vän, bekant, familjemedlem, flickvän / pojkvän eller släkting det yrket?**

**1 Ja**

**2 Nej**

**_______________________________________________________________________________**

**NY2B**

**( YRKE: Kock )**

**Bor den eller de personer i Sverige eller i annat land (eller både och)?**

**1 I Sverige**

**2 I annat land**

**3 Både i Sverige och i annat land**

**_______________________________________________________________________________**

**NY3A**

**YRKE: Byggnadsarbetare**

**Har någon vän, bekant, familjemedlem, flickvän / pojkvän eller släkting det yrket?**

**1 Ja**

**2 Nej**

**_______________________________________________________________________________**

**NY3B**

**( YRKE: Byggnadsarbetare )**

**Bor den eller de personer i Sverige eller i annat land (eller både och)?**

**1 I Sverige**

**2 I annat land**

**3 Både i Sverige och i annat land**

**_______________________________________________________________________________**

**NY4A**

**YRKE: Undersköterska**

**Har någon vän, bekant, familjemedlem, flickvän / pojkvän eller släkting det yrket?**

**1 Ja**

**2 Nej**

**_______________________________________________________________________________**

**NY4B**

**( YRKE: Undersköterska )**

**Bor den eller de personer i Sverige eller i annat land (eller både och)?**

**1 I Sverige**

**2 I annat land**

**3 Både i Sverige och i annat land**

**_______________________________________________________________________________**

**NY5A**

**YRKE: Ingenjör**

**Har någon vän, bekant, familjemedlem, flickvän / pojkvän eller släkting det yrket?**

**1 Ja**

**2 Nej**

**_______________________________________________________________________________**

**NY5B**

**( YRKE: Ingenjör )**

**Bor den eller de personer i Sverige eller i annat land (eller både och)?**

**1 I Sverige**

**2 I annat land**

**3 Både i Sverige och i annat land**

**_______________________________________________________________________________**

**NY6A**

**YRKE: Frisör**

**Har någon vän, bekant, familjemedlem, flickvän / pojkvän eller släkting det yrket?**

**1 Ja**

**2 Nej**

**_______________________________________________________________________________**

**NY6B**

**( YRKE: Frisör )**

**Bor den eller de personer i Sverige eller i annat land (eller både och)?**

**1 I Sverige**

**2 I annat land**

**3 Både i Sverige och i annat land**

**_______________________________________________________________________________**

**NY7A**

**YRKE: Brevbärare**

**Har någon vän, bekant, familjemedlem, flickvän / pojkvän eller släkting det yrket?**

**1 Ja**

**2 Nej**

**_______________________________________________________________________________**

**NY7B**

**( YRKE: Brevbärare )**

**Bor den eller de personer i Sverige eller i annat land (eller både och)?**

**1 I Sverige**

**2 I annat land**

**3 Både i Sverige och i annat land**

**_______________________________________________________________________________**

**NY8A**

**YRKE: Advokat**

**Har någon vän, bekant, familjemedlem, flickvän / pojkvän eller släkting det yrket?**

**1 Ja**

**2 Nej**

**_______________________________________________________________________________**

**NY8B**

**( YRKE: Advokat )**

**Bor den eller de personer i Sverige eller i annat land (eller både och)?**

**1 I Sverige**

**2 I annat land**

**3 Både i Sverige och i annat land**

**_______________________________________________________________________________**

**NY9A**

**YRKE: Personlig assistent**

**Har någon vän, bekant, familjemedlem, flickvän / pojkvän eller släkting det yrket?**

**1 Ja**

**2 Nej**

**_______________________________________________________________________________**

**NY9B**

**( YRKE: Personlig assistent )**

**Bor den eller de personer i Sverige eller i annat land (eller både och)?**

**1 I Sverige**

**2 I annat land**

**3 Både i Sverige och i annat land**

**_______________________________________________________________________________**

**NY10A**

**YRKE: Industriarbetare**

**Har någon vän, bekant, familjemedlem, flickvän / pojkvän eller släkting det yrket?**

**1 Ja**

**2 Nej**

**_______________________________________________________________________________**

**NY10B**

**( YRKE: Industriarbetare )**

**Bor den eller de personer i Sverige eller i annat land (eller både och)?**

**1 I Sverige**

**2 I annat land**

**3 Både i Sverige och i annat land**

**_______________________________________________________________________________**

**NY11A**

**YRKE: Telefonförsäljare**

**Har någon vän, bekant, familjemedlem, flickvän / pojkvän eller släkting det yrket?**

**1 Ja**

**2 Nej**

**_______________________________________________________________________________**

**NY11B**

**( YRKE: Telefonförsäljare )**

**Bor den eller de personer i Sverige eller i annat land (eller både och)?**

**1 I Sverige**

**2 I annat land**

**3 Både i Sverige och i annat land**

**_______________________________________________________________________________**

**NY12A**

**YRKE: Lärare på grundskola eller gymnasium**

**Har någon vän, bekant, familjemedlem, flickvän / pojkvän eller släkting det yrket?**

**1 Ja**

**2 Nej**

**_______________________________________________________________________________**

**NY12B**

**( YRKE: Lärare på grundskola eller gymnasium )**

**Bor den eller de personer i Sverige eller i annat land (eller både och)?**

**1 I Sverige**

**2 I annat land**

**3 Både i Sverige och i annat land**

**_______________________________________________________________________________**

**NY13A**

**YRKE: Sjuksköterska**

**Har någon vän, bekant, familjemedlem, flickvän / pojkvän eller släkting det yrket?**

**1 Ja**

**2 Nej**

**_______________________________________________________________________________**

**NY13B**

**( YRKE: Sjuksköterska )**

**Bor den eller de personer i Sverige eller i annat land (eller både och)?**

**1 I Sverige**

**2 I annat land**

**3 Både i Sverige och i annat land**

**_______________________________________________________________________________**

**NY14A**

**YRKE: Lastbilschaufför**

**Har någon vän, bekant, familjemedlem, flickvän / pojkvän eller släkting det yrket?**

**1 Ja**

**2 Nej**

**_______________________________________________________________________________**

**NY14B**

**( YRKE: Lastbilschaufför )**

**Bor den eller de personer i Sverige eller i annat land (eller både och)?**

**1 I Sverige**

**2 I annat land**

**3 Både i Sverige och i annat land**

**_______________________________________________________________________________**

**NY15A**

**YRKE: Fastighetsmäklare**

**Har någon vän, bekant, familjemedlem, flickvän / pojkvän eller släkting det yrket?**

**1 Ja**

**2 Nej**

**_______________________________________________________________________________**

**NY15B**

**( YRKE: Fastighetsmäklare )**

**Bor den eller de personer i Sverige eller i annat land (eller både och)?**

**1 I Sverige**

**2 I annat land**

**3 Både i Sverige och i annat land**

**_______________________________________________________________________________**

**NY16A**

**YRKE: Musiker på heltid**

**Har någon vän, bekant, familjemedlem, flickvän / pojkvän eller släkting det yrket?**

**1 Ja**

**2 Nej**

**_______________________________________________________________________________**

**NY16B**

**( YRKE: Musiker på heltid )**

**Bor den eller de personer i Sverige eller i annat land (eller både och)?**

**1 I Sverige**

**2 I annat land**

**3 Både i Sverige och i annat land**

**_______________________________________________________________________________**

**NY17A**

**YRKE: Polis**

**Har någon vän, bekant, familjemedlem, flickvän / pojkvän eller släkting det yrket?**

**1 Ja**

**2 Nej**

**_______________________________________________________________________________**

**NY17B**

**( YRKE: Polis )**

**Bor den eller de personer i Sverige eller i annat land (eller både och)?**

**1 I Sverige**

**2 I annat land**

**3 Både i Sverige och i annat land**

**_______________________________________________________________________________**

**NY18A**

**YRKE: Städare**

**Har någon vän, bekant, familjemedlem, flickvän / pojkvän eller släkting det yrket?**

**1 Ja**

**2 Nej**

**_______________________________________________________________________________**

**NY18B**

**( YRKE: Städare )**

**Bor den eller de personer i Sverige eller i annat land (eller både och)?**

**1 I Sverige**

**2 I annat land**

**3 Både i Sverige och i annat land**

**_______________________________________________________________________________**

**NY19A**

**YRKE: Tandläkare**

**Har någon vän, bekant, familjemedlem, flickvän / pojkvän eller släkting det yrket?**

**1 Ja**

**2 Nej**

**_______________________________________________________________________________**

**NY19B**

**( YRKE: Tandläkare )**

**Bor den eller de personer i Sverige eller i annat land (eller både och)?**

**1 I Sverige**

**2 I annat land**

**3 Både i Sverige och i annat land**

**_______________________________________________________________________________**

**NY20A**

**YRKE: Mekaniker, t.ex. bilmekaniker**

**Har någon vän, bekant, familjemedlem, flickvän / pojkvän eller släkting det yrket?**

**1 Ja**

**2 Nej**

**_______________________________________________________________________________**

**NY20B**

**( YRKE: Mekaniker, t.ex. bilmekaniker )**

**Bor den eller de personer i Sverige eller i annat land (eller både och)?**

**1 I Sverige**

**2 I annat land**

**3 Både i Sverige och i annat land**

**_______________________________________________________________________________**

**NY21A**

**YRKE: Barnskötare**

**Har någon vän, bekant, familjemedlem, flickvän / pojkvän eller släkting det yrket?**

**1 Ja**

**2 Nej**

**_______________________________________________________________________________**

**NY21B**

**( YRKE: Barnskötare )**

**Bor den eller de personer i Sverige eller i annat land (eller både och)?**

**1 I Sverige**

**2 I annat land**

**3 Både i Sverige och i annat land**

**_______________________________________________________________________________**

**NY22A**

**YRKE: Egenföretagare med egna anställda**

**Har någon vän, bekant, familjemedlem, flickvän / pojkvän eller släkting det yrket?**

**1 Ja**

**2 Nej**

**_______________________________________________________________________________**

**NY22B**

**( YRKE: Egenföretagare med egna anställda )**

**Bor den eller de personer i Sverige eller i annat land (eller både och)?**

**1 I Sverige**

**2 I annat land**

**3 Både i Sverige och i annat land**

**_______________________________________________________________________________**

**NY23A**

**YRKE: Kassapersonal**

**Har någon vän, bekant, familjemedlem, flickvän / pojkvän eller släkting det yrket?**

**1 Ja**

**2 Nej**

**_______________________________________________________________________________**

**NY23B**

**( YRKE: Kassapersonal )**

**Bor den eller de personer i Sverige eller i annat land (eller både och)?**

**1 I Sverige**

**2 I annat land**

**3 Både i Sverige och i annat land**

**_______________________________________________________________________________**

**NY24A**

**YRKE: Väktare**

**Har någon vän, bekant, familjemedlem, flickvän / pojkvän eller släkting det yrket?**

**1 Ja**

**2 Nej**

**_______________________________________________________________________________**

**NY24B**

**( YRKE: Väktare )**

**Bor den eller de personer i Sverige eller i annat land (eller både och)?**

**1 I Sverige**

**2 I annat land**

**3 Både i Sverige och i annat land**

**_______________________________________________________________________________**

**NY25A**

**YRKE: Journalist**

**Har någon vän, bekant, familjemedlem, flickvän / pojkvän eller släkting det yrket?**

**1 Ja**

**2 Nej**

**_______________________________________________________________________________**

**NY25B**

**( YRKE: Journalist )**

**Bor den eller de personer i Sverige eller i annat land (eller både och)?**

**1 I Sverige**

**2 I annat land**

**3 Både i Sverige och i annat land**

**_______________________________________________________________________________**

**NY26A**

**YRKE: Skådespelare på heltid**

**Har någon vän, bekant, familjemedlem, flickvän / pojkvän eller släkting det yrket?**

**1 Ja**

**2 Nej**

**_______________________________________________________________________________**

**NY26B**

**( YRKE: Skådespelare på heltid )**

**Bor den eller de personer i Sverige eller i annat land (eller både och)?**

**1 I Sverige**

**2 I annat land**

**3 Både i Sverige och i annat land**

**_______________________________________________________________________________**

**NY27A**

**YRKE: Receptionist**

**Har någon vän, bekant, familjemedlem, flickvän / pojkvän eller släkting det yrket?**

**1 Ja**

**2 Nej**

**_______________________________________________________________________________**

**NY27B**

**( YRKE: Receptionist )**

**Bor den eller de personer i Sverige eller i annat land (eller både och)?**

**1 I Sverige**

**2 I annat land**

**3 Både i Sverige och i annat land**

**_______________________________________________________________________________**

**NY28A**

**YRKE: Ekonomichef**

**Har någon vän, bekant, familjemedlem, flickvän / pojkvän eller släkting det yrket?**

**1 Ja**

**2 Nej**

**_______________________________________________________________________________**

**NY28B**

**( YRKE: Ekonomichef )**

**Bor den eller de personer i Sverige eller i annat land (eller både och)?**

**1 I Sverige**

**2 I annat land**

**3 Både i Sverige och i annat land**

**_______________________________________________________________________________**

**NY29A**

**YRKE: Student på universitet**

**Har någon vän, bekant, familjemedlem, flickvän / pojkvän eller släkting det yrket?**

**1 Ja**

**2 Nej**

**_______________________________________________________________________________**

**NY29B**

**( YRKE: Student på universitet )**

**Bor den eller de personer i Sverige eller i annat land (eller både och)?**

**1 I Sverige**

**2 I annat land**

**3 Både i Sverige och i annat land**

**_______________________________________________________________________________**

**NY30A**

**YRKE: Taxichaufför**

**Har någon vän, bekant, familjemedlem, flickvän / pojkvän eller släkting det yrket?**

**1 Ja**

**2 Nej**

**_______________________________________________________________________________**

**NY30B**

**( YRKE: Taxichaufför )**

**Bor den eller de personer i Sverige eller i annat land (eller både och)?**

**1 I Sverige**

**2 I annat land**

**3 Både i Sverige och i annat land**

**_______________________________________________________________________________**

**NY31A**

**YRKE: Rektor**

**Har någon vän, bekant, familjemedlem, flickvän / pojkvän eller släkting det yrket?**

**1 Ja**

**2 Nej**

**_______________________________________________________________________________**

**NY31B**

**( YRKE: Rektor )**

**Bor den eller de personer i Sverige eller i annat land (eller både och)?**

**1 I Sverige**

**2 I annat land**

**3 Både i Sverige och i annat land**

**_______________________________________________________________________________**

**NY32A**

**YRKE: Datatekniker**

**Har någon vän, bekant, familjemedlem, flickvän / pojkvän eller släkting det yrket?**

**1 Ja**

**2 Nej**

**_______________________________________________________________________________**

**NY32B**

**( YRKE: Datatekniker )**

**Bor den eller de personer i Sverige eller i annat land (eller både och)?**

**1 I Sverige**

**2 I annat land**

**3 Både i Sverige och i annat land**

**_______________________________________________________________________________**

**NY33A**

**YRKE: Fritidsledare**

**Har någon vän, bekant, familjemedlem, flickvän / pojkvän eller släkting det yrket?**

**1 Ja**

**2 Nej**

**_______________________________________________________________________________**

**NY33B**

**( YRKE: Fritidsledare )**

**Bor den eller de personer i Sverige eller i annat land (eller både och)?**

**1 I Sverige**

**2 I annat land**

**3 Både i Sverige och i annat land**

**_______________________________________________________________________________**

**NY34A**

**YRKE: Banktjänsteman**

**Har någon vän, bekant, familjemedlem, flickvän / pojkvän eller släkting det yrket?**

**1 Ja**

**2 Nej**

**_______________________________________________________________________________**

**NY34B**

**( YRKE: Banktjänsteman )**

**Bor den eller de personer i Sverige eller i annat land (eller både och)?**

**1 I Sverige**

**2 I annat land**

**3 Både i Sverige och i annat land**

**_______________________________________________________________________________**

**NY35A**

**YRKE: Lagerarbetare**

**Har någon vän, bekant, familjemedlem, flickvän / pojkvän eller släkting det yrket?**

**1 Ja**

**2 Nej**

**_______________________________________________________________________________**

**NY35B**

**( YRKE: Lagerarbetare )**

**Bor den eller de personer i Sverige eller i annat land (eller både och)?**

**1 I Sverige**

**2 I annat land**

**3 Både i Sverige och i annat land**

**_______________________________________________________________________________**

**NY36A**

**YRKE: Datorprogrammerare**

**Har någon vän, bekant, familjemedlem, flickvän / pojkvän eller släkting det yrket?**

**1 Ja**

**2 Nej**

**_______________________________________________________________________________**

**NY36B**

**( YRKE: Datorprogrammerare )**

**Bor den eller de personer i Sverige eller i annat land (eller både och)?**

**1 I Sverige**

**2 I annat land**

**3 Både i Sverige och i annat land**

**_______________________________________________________________________________**

**NY37A**

**YRKE: Revisor**

**Har någon vän, bekant, familjemedlem, flickvän / pojkvän eller släkting det yrket?**

**1 Ja**

**2 Nej**

**_______________________________________________________________________________**

**NY37B**

**( YRKE: Revisor )**

**Bor den eller de personer i Sverige eller i annat land (eller både och)?**

**1 I Sverige**

**2 I annat land**

**3 Både i Sverige och i annat land**

**_______________________________________________________________________________**

**NY38A**

**YRKE: Vaktmästare**

**Har någon vän, bekant, familjemedlem, flickvän / pojkvän eller släkting det yrket?**

**1 Ja**

**2 Nej**

**_______________________________________________________________________________**

**NY38B**

**( YRKE: Vaktmästare )**

**Bor den eller de personer i Sverige eller i annat land (eller både och)?**

**1 I Sverige**

**2 I annat land**

**3 Både i Sverige och i annat land**

**_______________________________________________________________________________**

**NY39A**

**YRKE: Forskare**

**Har någon vän, bekant, familjemedlem, flickvän / pojkvän eller släkting det yrket?**

**1 Ja**

**2 Nej**

**_______________________________________________________________________________**

**NY39B**

**( YRKE: Forskare )**

**Bor den eller de personer i Sverige eller i annat land (eller både och)?**

**1 I Sverige**

**2 I annat land**

**3 Både i Sverige och i annat land**

**_______________________________________________________________________________**

**NY40A**

**YRKE: Servitör eller servitris**

**Har någon vän, bekant, familjemedlem, flickvän / pojkvän eller släkting det yrket?**

**1 Ja**

**2 Nej**

**_______________________________________________________________________________**

**NY40B**

**YRKE: Servitör eller servitris**

**Bor den eller de personer i Sverige eller i annat land (eller både och)?**

**1 I Sverige**

**2 I annat land**

**3 Både i Sverige och i annat land**

**_______________________________________________________________________________**

**F91**

**Tack! Nu skulle jag vilja gå över till att ställa några frågor som handlar**

**om din nuvarande sysselsättning ...**

**Vilken är din nuvarande sysselsättning?**

**Studerar du, arbetar du, är du arbetslös, eller gör du något annat?**

**1 Studerar enbart**

**2 Arbetar enbart**

**3 Studerar och arbetar**

**4 Arbetslös**

**5 Gör något annat ...**

**_______________________________________________________________________________**

**F92**

**Vad skulle du själv säga är din nuvarande sysselsättning?**

**Är du hemma och sköter hushållet, eller är du arbetslös, eller sjukskriven,**

**eller gör du ingenting, eller är det något annat du gör?**

**1 Sköter hushåll (EJ SOM LÖNEANSTÄLLD)**

**2 Sjukskriven**

**3 Ingenting**

**4 Annat, nämligen ...**

**_______________________________________________________________________________**

**F92TXT**

**( Vad skulle du själv säga är din nuvarande sysselsättning? )**

**ANGE ANNAN SYSSELSÄTTNING:**

**_______________________________________________________________________________**

**F103AS1**

**VISAS EJ PÅ SKÄRMEN!**

**Här lagras svar från F103A:**

**1 Nej**

**_______________________________________________________________________________**

**F103AS2**

**VISAS EJ PÅ SKÄRMEN!**

**Här lagras svar från F103A:**

**2 Ja, komvux**

**_______________________________________________________________________________**

**F103AS3**

**VISAS EJ PÅ SKÄRMEN!**

**Här lagras svar från F103A:**

**3 Ja, universitet / högskola**

**_______________________________________________________________________________**

**F103AS4**

**VISAS EJ PÅ SKÄRMEN!**

**Här lagras svar från F103A:**

**4 Ja, folkhögskola**

**_______________________________________________________________________________**

**F103AS5**

**VISAS EJ PÅ SKÄRMEN!**

**Här lagras svar från F103A:**

**5 Ja, annan utbildning ...**

**_______________________________________________________________________________**

**F103A**

**<<TEXT 'Läser du ' om F91 = (1, 3) >><<TEXT 'Har du läst ' om F91^= (1, 3) >>vid komvux, universitet / högskola, folkhögskola eller någon annan utbildning?**

**FLERA SVAR KAN ANGES. TRYCK MELLANSLAG MELLAN SVAREN.**

**<<TEXT '1 Nej ' om F91^= (1, 3) >>**

**2 Ja, komvux**

**3 Ja, universitet / högskola**

**4 Ja, folkhögskola**

**5 Ja, annan utbildning ...**

**_______________________________________________________________________________**

**F103ATXT**

**( <<TEXT 'Läser du ' om F91 = (1, 3) >><<TEXT 'Har du läst ' om F91^= (1, 3) >>vid komvux, universitet / högskola, folkhögskola eller någon annan utbildning? )**

**ANGE ANNAN UTBILDNING:**

**_______________________________________________________________________________**

**F103B**

**Har du avslutat (dvs gått ut) den utbildningen?**

**1 Ja**

**2 Nej**

**_______________________________________________________________________________**

**F103C**

**För tillfället, har du ett eller flera jobb?**

**Räkna även med jobb som du nu kanske har semester ifrån eller är sjukledig ifrån.**

**1 Ja, ett jobb**

**2 Ja, flera jobb**

**<<TEXT '3 Nej, inget jobb ' om F91^= (2, 3) >>**

**_______________________________________________________________________________**

**F103D**

**Till vilket yrke vill du räkna ditt arbete?**

**1 = YRKET FINNS INTE I LISTAN**

**YRKE:**

**_______________________________________________________________________________**

**F103DTXT**

**( Till vilket yrke vill du räkna ditt arbete? )**

**___________________________________________________________________________**

**TILL IVE: Försök ta reda på följande**

**- Huvudsakliga arbetsuppgifter**

**- Bransch**

**- Verksamhet (ex hemtjänst äldreomsorg, bygg inom mur och puts)**

**- Kvalifikationsnivå (ex ledningsarbete, militärt arbete, arbetare och tjänstemän)**

**___________________________________________________________________________**

**ANGE YRKE I KLARTEXT:**

**_______________________________________________________________________________**

**F103E**

**Hur många jobb har du för tillfället?**

**ANGE ANTAL JOBB:**

**_______________________________________________________________________________**

**F103F**

**Till vilket yrke vill du räkna ditt huvudsakliga arbete?**

**~~~~~~~~~~**

**1 = YRKET FINNS INTE I LISTAN**

**YRKE:**

**_______________________________________________________________________________**

**F103FTXT**

**( Till vilket yrke vill du räkna ditt huvudsakliga arbete? )**

**~~~~~~~~~~**

**___________________________________________________________________________**

**TILL IVE: Försök ta reda på följande**

**- Huvudsakliga arbetsuppgifter**

**- Bransch**

**- Verksamhet (ex hemtjänst äldreomsorg, bygg inom mur och puts)**

**- Kvalifikationsnivå (ex ledningsarbete, militärt arbete, arbetare och tjänstemän)**

**___________________________________________________________________________**

**ANGE YRKE I KLARTEXT:**

**_______________________________________________________________________________**

**F103G**

**Är din arbetsgivare en familjemedlem?**

**1 Ja**

**2 Nej**

**_______________________________________________________________________________**

**F103H**

**Jobbar du heltid eller deltid?**

**::**

**1 Heltid**

**2 Deltid**

**_______________________________________________________________________________**

**F103I**

**Är jobbet fast eller tidsbegränsat?**

**1 Fast**

**2 Tidsbegränsat**

**_______________________________________________________________________________**

**F103J**

**Var det någon av de personer du nämnde förut som hjälpt dig att få jobbet?**

**___________________________________________________________________**

**TILL IVE: Följande vänner har uppgetts tidigare i intervjun:**

**<<TEXT Van1Namn >>**

**<<TEXT Van2Namn >>**

**<<TEXT Van3Namn >>**

**<<TEXT Van4Namn >>**

**<<TEXT Van5Namn >>**

**___________________________________________________________________**

**1 Ja**

**2 Nej**

**_______________________________________________________________________________**

**F103JNAMN**

**VISAS EJ PÅ SKÄRMEN!**

**Här lagras namnet på den person som anges i fråga F103JJ.**

**_______________________________________________________________________________**

**F103JJ**

**Vem av dem?**

**LÄS UPP SVARSALTERNATIVEN VID BEHOV!**

**<<TEXT '1 ' om AntalVanner >= 1 >><<TEXT Van1Namn >>**

**<<TEXT '2 ' om AntalVanner >= 2 >><<TEXT Van2Namn >>**

**<<TEXT '3 ' om AntalVanner >= 3 >><<TEXT Van3Namn >>**

**<<TEXT '4 ' om AntalVanner >= 4 >><<TEXT Van4Namn >>**

**<<TEXT '5 ' om AntalVanner >= 5 >><<TEXT Van5Namn >>**

**_______________________________________________________________________________**

**F104**

**Har du under de senaste tre åren haft sommarjobb eller extrajobb?**

**1 Ja**

**2 Nej**

**_______________________________________________________________________________**

**F105B**

**Var det något av dem namn du nämnde förut som hjälpt dig att få jobbet?**

**___________________________________________________________________**

**TILL IVE: Följande vänner har uppgetts tidigare i intervjun:**

**<<TEXT Van1Namn >>**

**<<TEXT Van2Namn >>**

**<<TEXT Van3Namn >>**

**<<TEXT Van4Namn >>**

**<<TEXT Van5Namn >>**

**___________________________________________________________________**

**1 Ja**

**2 Nej**

**_______________________________________________________________________________**

**F105BNAMN**

**VISAS EJ PÅ SKÄRMEN!**

**Här lagras namnet på den person som anges i fråga F105BB.**

**_______________________________________________________________________________**

**F105BB**

**Vem av dem?**

**LÄS UPP SVARSALTERNATIVEN VID BEHOV!**

**<<TEXT '1 ' om AntalVanner >= 1 >><<TEXT Van1Namn >>**

**<<TEXT '2 ' om AntalVanner >= 2 >><<TEXT Van2Namn >>**

**<<TEXT '3 ' om AntalVanner >= 3 >><<TEXT Van3Namn >>**

**<<TEXT '4 ' om AntalVanner >= 4 >><<TEXT Van4Namn >>**

**<<TEXT '5 ' om AntalVanner >= 5 >><<TEXT Van5Namn >>**

**_______________________________________________________________________________**

**F106B**

**Om du fick erbjudande om en fast anställning på det jobbet,**

**skulle du vara intresserad av det?**

**---------------------------------------------------------------------------------------------**

**TILL IVE: Frågan avser det sommarjobb / extrajobb som UP haft**

**under de senaste tre åren.**

**---------------------------------------------------------------------------------------------**

**1 Ja**

**2 Nej**

**_______________________________________________________________________________**

**F107**

**Håller du på att söka arbete som inte är sommarjobb eller extrajobb?**

**1 Ja**

**2 Nej**

**_______________________________________________________________________________**

**F108S1**

**VISAS EJ PÅ SKÄRMEN!**

**Här lagras svar från F108:**

**1 Genom arbetsförmedlingen**

**_______________________________________________________________________________**

**F108S2**

**VISAS EJ PÅ SKÄRMEN!**

**Här lagras svar från F108:**

**2 Läser annonser i tidningen**

**_______________________________________________________________________________**

**F108S3**

**VISAS EJ PÅ SKÄRMEN!**

**Här lagras svar från F108:**

**3 Jobbsajter på nätet**

**_______________________________________________________________________________**

**F108S4**

**VISAS EJ PÅ SKÄRMEN!**

**Här lagras svar från F108:**

**4 Ringer / skriver brev till arbetsgivare**

**_______________________________________________________________________________**

**F108S5**

**VISAS EJ PÅ SKÄRMEN!**

**Här lagras svar från F108:**

**5 Frågar runt bland vänner och släktingar**

**_______________________________________________________________________________**

**F108S6**

**VISAS EJ PÅ SKÄRMEN!**

**Här lagras svar från F108:**

**6 Frågar nuvarande arbetsgivare**

**_______________________________________________________________________________**

**F108S7**

**VISAS EJ PÅ SKÄRMEN!**

**Här lagras svar från F108:**

**7 Annat ...**

**_______________________________________________________________________________**

**F108**

**Om du <<TEXT 'söker ' om F107 = 1 >><<TEXT 'skulle söka ' om F107^= 1 >>arbete - varifrån <<TEXT 'får du ' om F107 = 1 >><<TEXT 'skulle du få ' om F107^= 1 >>information om lediga jobb?**

**Från arbetsförmedlingen, genom att läsa annonser i tidningen, jobbsajter på nätet,**

**genom att ringa eller skriva till arbetsgivare, fråga runt bland vänner och släktingar,**

**fråga nuvarande arbetsgivare, eller något annat?**

**____________________________________________________________**

**TILL IVE: Läs ett svarsalternativ i taget och notera UP:s svar.**

**____________________________________________________________**

**FLERA SVAR KAN ANGES. TRYCK MELLANSLAG MELLAN SVAREN.**

**1 Genom arbetsförmedlingen**

**2 Läser annonser i tidningen**

**3 Jobbsajter på nätet**

**4 Ringer / skriver brev till arbetsgivare**

**5 Frågar runt bland vänner och släktingar**

**6 Frågar nuvarande arbetsgivare**

**7 Annat ...**

**_______________________________________________________________________________**

**F108TXT**

**( Om du <<TEXT 'söker ' om F107 = 1 >><<TEXT 'skulle söka ' om F107^= 1 >>arbete - varifrån <<TEXT 'får du ' om F107 = 1 >><<TEXT 'skulle du få ' om F107^= 1 >>information om lediga jobb? )**

**ANGE ANNAN SÖKMETOD I KLARTEXT:**

**_______________________________________________________________________________**

**F108C**

**Om du <<TEXT 'söker ' om F107 = 1 >><<TEXT 'skulle söka ' om F107^= 1 >>jobb nu, hur goda chanser tror du att du själv har att få ett (nytt) arbete inom 6 månader?**

**Mycket goda, goda, någorlunda, dåliga eller mycket dåliga?**

**1 Mycket goda**

**2 Goda**

**3 Någorlunda**

**4 Dåliga**

**5 Mycket dåliga**

**_______________________________________________________________________________**

**F108D**

**Om du ser tillbaka på de senaste 12 månaderna, har någon nämnt eller tipsat dig personligen**

**om jobb utan att du frågat, t.ex. via samtal, telefon, email?**

**1 Ja**

**2 Nej**

**_______________________________________________________________________________**

**F108E**

**Hur många sådana tips har du fått?**

**ANTAL TIPS:**

**_______________________________________________________________________________**

**F131**

**Nu kommer jag att ställa en del frågor som bland annat handlar om hälsa.**

**Jag börjar med motion under fritiden.**

**Motionerar du regelbundet minst en gång i veckan på din fritid?**

**Räkna bara motionspass som är 30 minuter eller längre.**

**1 Ja**

**2 Nej**

**_______________________________________________________________________________**

**F132**

**Hur många dagar i veckan motionerar du sammanhängande under minst 30 minuter?**

**_______________________________________________________________**

**TILL IVE: Avser en normal vecka. om UP har svårt att svara exakt,**

**ange det lägre alternativet. T.ex. "3 - 4 dagar", ange 3.**

**_______________________________________________________________**

**ANTAL DAGAR PER VECKA:**

**_______________________________________________________________________________**

**F143**

**Hur lång är du?**

**LÄNGD (CM):**

**_______________________________________________________________________________**

**F144**

**Ungefär hur mycket väger du?**

**VIKT (KG):**

**_______________________________________________________________________________**

**F145**

**När du väljer vad du skall äta, hur viktigt är det att maten är nyttig och hälsosam?**

**1 Mycket viktigt**

**2 Ganska viktigt**

**3 Oviktigt**

**_______________________________________________________________________________**

**F150**

**Hur bedömer du ditt allmänna hälsotillstånd?**

**Är det mycket bra, bra, någorlunda, dåligt eller mycket dåligt?**

**1 Mycket bra**

**2 Bra**

**3 Någorlunda**

**4 Dåligt**

**5 Mycket dåligt**

**_______________________________________________________________________________**

**F160**

**Använder du regelbundet någon receptbelagd medicin?**

**1 Ja**

**2 Nej**

**_______________________________________________________________________________**

**F171**

**Har du under de senaste 12 månaderna haft någon av följande sjukdomar eller besvär...**

**... huvudvärk eller migrän?**

**LÄS UPP SVARSALTERNATIVEN!**

**1 Svåra besvär**

**2 Lätta besvär**

**3 Inga besvär**

**_______________________________________________________________________________**

**F172**

**( Har du under de senaste 12 månaderna haft ... )**

**... Magont eller magvärk?**

**1 Svåra besvär**

**2 Lätta besvär**

**3 Inga besvär**

**_______________________________________________________________________________**

**F173**

**( Har du under de senaste 12 månaderna haft ... )**

**... Smärtor i ryggen eller nacke?**

**1 Svåra besvär**

**2 Lätta besvär**

**3 Inga besvär**

**_______________________________________________________________________________**

**F174**

**( Har du under de senaste 12 månaderna haft ... )**

**... Allmän trötthet?**

**_______________________________________________________________________________**

**FÖRTYDLIGA VID BEHOV: Hängig och orkeslös under dagar och kvällar, inte bara morgontrött.**

**_______________________________________________________________________________**

**1 Svåra besvär**

**2 Lätta besvär**

**3 Inga besvär**

**_______________________________________________________________________________**

**F175**

**( Har du under de senaste 12 månaderna haft ... )**

**... Sömnbesvär?**

**____________________________________________________**

**FÖRTYDLIGA VID BEHOV: Svårt att sova på nätterna.**

**____________________________________________________**

**1 Svåra besvär**

**2 Lätta besvär**

**3 Inga besvär**

**_______________________________________________________________________________**

**F176**

**( Har du under de senaste 12 månaderna haft ... )**

**... Ängslan, oro eller ångest?**

**1 Svåra besvär**

**2 Lätta besvär**

**3 Inga besvär**

**_______________________________________________________________________________**

**F177**

**( Har du under de senaste 12 månaderna haft ... )**

**... Depression?**

**____________________________________________________**

**FÖRTYDLIGA VID BEHOV: Nedstämdhet.**

**____________________________________________________**

**1 Svåra besvär**

**2 Lätta besvär**

**3 Inga besvär**

**_______________________________________________________________________________**

**F1720**

**Hur ofta brukar du vara förkyld?**

**1 Mycket ofta**

**2 Ganska ofta**

**3 Inte speciellt ofta**

**4 Aldrig**

**_______________________________________________________________________________**

**F181**

**Röker du dagligen?**

**1 Ja**

**2 Nej**

**_______________________________________________________________________________**

**F182**

**Hur många cigaretter röker du om dagen i genomsnitt?**

**_________________________________________**

**TILL IVE: 1 paket = 20 cigaretter**

**_________________________________________**

**ANTAL CIGARETTER PER DAG:**

**_______________________________________________________________________________**

**F183**

**Händer det att du röker någon gång då och då?**

**1 Ja**

**2 Nej**

**_______________________________________________________________________________**

**F191**

**Har du bantat under de senaste 12 månaderna?**

**1 Ja**

**2 Nej**

**_______________________________________________________________________________**

**F192**

**Vid hur många tillfällen har du bantat de 12 senaste månaderna?**

**__________________________________________________________________________**

**TILL IVE: Vad som menas med ett tillfälle och längden på uppehållet mellan två tillfällen**

**definieras av UP själv. Om UP anser sig ha batat hela året utan avbrott,**

**ange 1 tillfälle.**

**__________________________________________________________________________**

**ANGE ANTAL GÅNGER:**

**_______________________________________________________________________________**

**F201**

**Dricker du alkohol?**

**1 Ja**

**2 Nej**

**_______________________________________________________________________________**

**F202**

**Hur ofta?**

**LÄS UPP SVARSALTERNATIVEN!**

**1 Tre dagar i veckan eller oftare**

**2 En till två gånger per vecka**

**3 Två till tre gånger per månad**

**4 En gång per månad**

**5 Mer sällan**

**_______________________________________________________________________________**

**F203**

**Ungefär hur många gånger har du, under de 12 senaste månaderna,**

**druckit så mycket alkohol att du blivit berusad?**

**1 Tre dagar i veckan eller oftare**

**2 En till två gånger per vecka**

**3 Två till tre gånger per månad**

**4 En gång per månad**

**5 Mer sällan**

**6 Aldrig**

**_______________________________________________________________________________**

**F211**

**Har du under de 12 senaste månaderna rökt cannabis?**

**1 Ja**

**2 Nej**

**_______________________________________________________________________________**

**F221**

**Har du under de senaste 3 månaderna, med andra ord de senaste 90 dagarna,**

**på grund av egen sjukdom besökt läkare vid vårdcentral, privatläkarmottagning,**

**sjukhus eller skolläkare?**

**1 Ja**

**2 Nej**

**_______________________________________________________________________________**

**F222**

**Hur många gånger?**

**ANGE ANTAL GÅNGER:**

**_______________________________________________________________________________**

**F222A**

**Har du under de senaste 12 månaderna utsatts för någon typ av brott?**

**1 Ja**

**2 Nej**

**_______________________________________________________________________________**

**F222BS1**

**VISAS EJ PÅ SKÄRMEN!**

**Här lagras svar från F222B:**

**1 Hot om våld**

**_______________________________________________________________________________**

**F222BS2**

**VISAS EJ PÅ SKÄRMEN!**

**Här lagras svar från F222B:**

**2 Sexuella övergrepp**

**_______________________________________________________________________________**

**F222BS3**

**VISAS EJ PÅ SKÄRMEN!**

**Här lagras svar från F222B:**

**3 Sexuella trakasserier**

**_______________________________________________________________________________**

**F222BS4**

**VISAS EJ PÅ SKÄRMEN!**

**Här lagras svar från F222B:**

**4 Misshandel**

**_______________________________________________________________________________**

**F222BS5**

**VISAS EJ PÅ SKÄRMEN!**

**Här lagras svar från F222B:**

**5 Rån**

**_______________________________________________________________________________**

**F222BS6**

**VISAS EJ PÅ SKÄRMEN!**

**Här lagras svar från F222B:**

**6 Blivit bestulen på något värdefullt**

**_______________________________________________________________________________**

**F222BS7**

**VISAS EJ PÅ SKÄRMEN!**

**Här lagras svar från F222B:**

**7 Inbrott**

**_______________________________________________________________________________**

**F222BS8**

**VISAS EJ PÅ SKÄRMEN!**

**Här lagras svar från F222B:**

**8 Vapenhot**

**_______________________________________________________________________________**

**F222BS9**

**VISAS EJ PÅ SKÄRMEN!**

**Här lagras svar från F222B:**

**9 Mobbing**

**_______________________________________________________________________________**

**F222BS10**

**VISAS EJ PÅ SKÄRMEN!**

**Här lagras svar från F222B:**

**10 Annat**

**_______________________________________________________________________________**

**F222B**

**Vilken typ av brott?**

**TILLÄGGSFRÅGA: Har du blivit utsatt för något mer?**

**FLERA SVAR KAN ANGES. TRYCK MELLANSLAG MELLAN SVAREN.**

**1 Hot om våld**

**2 Sexuella övergrepp**

**3 Sexuella trakasserier**

**4 Misshandel**

**5 Rån**

**6 Blivit bestulen på något värdefullt**

**7 Inbrott**

**8 Vapenhot**

**9 Mobbing**

**10 Annat**

**_______________________________________________________________________________**

**F222C**

**Brukar du oroa dig över att utsättas för brottshandlingar?**

**1 Ja, ofta**

**2 Ja, ibland**

**3 Nej**

**_______________________________________________________________________________**

**F222D**

**Är du rädd för att promenera ensam i ditt bostadsområde?**

**1 Ja, ofta**

**2 Ja, ibland**

**3 Nej**

**_______________________________________________________________________________**

**F223A**

**Nu skall jag gå över till andra frågor.**

**Om du skulle beskriva vem du är, vad skulle vara VIKTIGAST för dig**

**att säga något om? Att Du ...**

**LÄS UPP SVARSALTERNATIVEN!**

**1 ... har en viss religion?**

**2 ... är född i ett visst land?**

**3 ... har ett visst kön?**

**4 ... har en viss politisk åsikt?**

**5 ... har en viss sexuell läggning?**

**6 ... tillhör en viss klass i samhället?**

**7 ... har en viss musikstil?**

**8 ... bor i ett visst område / ställe?**

**9 ... tillhör en viss etnisk grupp?**

**10 ... eller att du hejar på ett visst lag?**

**_______________________________________________________________________________**

**F223B**

**Vad skulle vara NÄST VIKTIGAST för dig att säga något om? Att du ...**

**LÄS UPP SVARSALTERNATIVEN!**

**<<TEXT ' 1 ... har en viss religion? ' om F223A^= 1 >>**

**<<TEXT ' 2 ... är född i ett visst land? ' om F223A^= 2 >>**

**<<TEXT ' 3 ... har ett visst kön? ' om F223A^= 3 >>**

**<<TEXT ' 4 ... har en viss politisk åsikt? ' om F223A^= 4 >>**

**<<TEXT ' 5 ... har en viss sexuell läggning? ' om F223A^= 5 >>**

**<<TEXT ' 6 ... tillhör en viss klass i samhället? ' om F223A^= 6 >>**

**<<TEXT ' 7 ... har en viss musikstil? ' om F223A^= 7 >>**

**<<TEXT ' 8 ... bor i ett visst område / ställe? ' om F223A^= 8 >>**

**<<TEXT ' 9 ... tillhör en viss etnisk grupp? ' om F223A^= 9 >>**

**<<TEXT '10 ... eller att du hejar på ett visst lag? ' om F223A^= 10 >>**

**_______________________________________________________________________________**

**F223C**

**Vad skulle vara TREDJE VIKTIGAST för dig att säga något om? Att du ...**

**LÄS UPP SVARSALTERNATIVEN!**

**<<TEXT ' 1 ... har en viss religion? ' om F223A^= 1 & F223B^= 1 >>**

**<<TEXT ' 2 ... är född i ett visst land? ' om F223A^= 2 & F223B^= 2 >>**

**<<TEXT ' 3 ... har ett visst kön? ' om F223A^= 3 & F223B^= 3 >>**

**<<TEXT ' 4 ... har en viss politisk åsikt? ' om F223A^= 4 & F223B^= 4 >>**

**<<TEXT ' 5 ... har en viss sexuell läggning? ' om F223A^= 5 & F223B^= 5 >>**

**<<TEXT ' 6 ... tillhör en viss klass i samhället? ' om F223A^= 6 & F223B^= 6 >>**

**<<TEXT ' 7 ... har en viss musikstil? ' om F223A^= 7 & F223B^= 7 >>**

**<<TEXT ' 8 ... bor i ett visst område / ställe? ' om F223A^= 8 & F223B^= 8 >>**

**<<TEXT ' 9 ... tillhör en viss etnisk grupp? ' om F223A^= 9 & F223B^= 9 >>**

**<<TEXT '10 ... eller att du hejar på ett visst lag? ' om F223A^= 10 & F223B^= 10 >>**

**_______________________________________________________________________________**

**F224**

**Om du skulle beskriva var du kommer ifrån, skulle det vara VIKTIGAST att tala om**

**att du är från en viss del av världen - alltså kontinent eller världsdel -**

**eller från ett visst land, eller från en viss del av landet, eller från en viss plats eller ort?**

**1 Viss kontinent**

**2 Visst land**

**3 Visst område**

**4 Viss ort**

**_______________________________________________________________________________**

**F224B**

**Om du skulle beskriva var du kommer ifrån, vad skulle vara NÄST VIKTIGAST?**

**LÄS UPP SVARSALTERNATIVEN!**

**<<TEXT '1 Viss kontinent? ' om F224^= 1 >>**

**<<TEXT '2 Visst land? ' om F224^= 2 >>**

**<<TEXT '3 Visst område? ' om F224^= 3 >>**

**<<TEXT '4 Viss ort? ' om F224^= 4 >>**

**_______________________________________________________________________________**

**F231**

**Människor använder olika uttryck för att beskriva sin etniska tillhörighet.**

**Vilken beteckning tycker du beskriver dig själv bäst?**

**1 Svensk**

**<<TEXT ' 2 Iranier ' om Grupp = 'IR' >>**

**<<TEXT ' 3 Kurd ' om Grupp = 'IR' >>**

**<<TEXT ' 4 Perser ' om Grupp = 'IR' >><<TEXT ' 5 Invandrare ' om Grupp = 'JU' >>**

**<<TEXT ' 5 Invandrare ' om Grupp = 'IR' >><<TEXT ' 6 Kroat ' om Grupp = 'JU' >>**

**<<TEXT ' ' om Grupp = 'IR' >><<TEXT ' 7 Serb ' om Grupp = 'JU' >>**

**<<TEXT ' ' om Grupp = 'IR' >><<TEXT ' 8 Sloven ' om Grupp = 'JU' >>**

**<<TEXT '13 Annan ... ' om Grupp = 'IR' >><<TEXT ' 9 Bosnier ' om Grupp = 'JU' >>**

**<<TEXT ' ' om Grupp = 'IR' >><<TEXT '10 Kosovoalban ' om Grupp = 'JU' >>**

**<<TEXT ' ' om Grupp = 'IR' >><<TEXT '11 Makedonier ' om Grupp = 'JU' >>**

**<<TEXT ' ' om Grupp = 'IR' >><<TEXT '12 Jugoslav ' om Grupp = 'JU' >>**

**<<TEXT ' ' om Grupp = 'IR' >>**

**<<TEXT ' ' om Grupp = 'IR' >><<TEXT '13 Annan ... ' om Grupp = 'JU' >>**

**_______________________________________________________________________________**

**F231TXT**

**( Människor använder olika uttryck för att beskriva sin etniska tillhörighet.**

**Vilken beteckning tycker du beskriver dig själv bäst? )**

**ANGE ANNAN BETECKNING:**

**_______________________________________________________________________________**

**F231B**

**Är det någon annan beteckning som du också tycker passar in på dig själv?**

**_______________________________________________________________________**

**TILL IVE: Om UP anger fler än 1 beteckning, be UP välja den som passar allra bäst.**

**_______________________________________________________________________**

**0 NEJ, INGEN YTTERLIGARE BETECKNING PASSAR IN**

**<<TEXT ' 1 Svensk ' om F231^= 1 >>**

**<<TEXT ' 2 Iranier ' om Grupp = 'IR' & F231^= 2 >>**

**<<TEXT ' 3 Kurd ' om Grupp = 'IR' & F231^= 3 >>**

**<<TEXT ' 4 Perser ' om Grupp = 'IR' & F231^= 4 >><<TEXT ' 5 Invandrare ' om Grupp = 'JU' & F231^= 5 >>**

**<<TEXT ' 5 Invandrare ' om Grupp = 'IR' & F231^= 5 >><<TEXT ' 6 Kroat ' om Grupp = 'JU' & F231^= 6 >>**

**<<TEXT ' ' om Grupp = 'IR' >><<TEXT ' 7 Serb ' om Grupp = 'JU' & F231^= 7 >>**

**<<TEXT ' ' om Grupp = 'IR' >><<TEXT ' 8 Sloven ' om Grupp = 'JU' & F231^= 8 >>**

**<<TEXT '13 Annan ... ' om Grupp = 'IR' >><<TEXT ' 9 Bosnier ' om Grupp = 'JU' & F231^= 9 >>**

**<<TEXT ' ' om Grupp = 'IR' >><<TEXT '10 Kosovoalban ' om Grupp = 'JU' & F231^= 10 >>**

**<<TEXT ' ' om Grupp = 'IR' >><<TEXT '11 Makedonier ' om Grupp = 'JU' & F231^= 11 >>**

**<<TEXT ' ' om Grupp = 'IR' >><<TEXT '12 Jugoslav ' om Grupp = 'JU' & F231^= 12 >>**

**<<TEXT ' ' om Grupp = 'IR' >>**

**<<TEXT ' ' om Grupp = 'IR' >><<TEXT '13 Annan ... ' om Grupp = 'JU' & F231^= 13 >>**

**_______________________________________________________________________________**

**F231BTXT**

**( Är det någon annan beteckning som du också tycker passar in på dig själv? )**

**ANGE ANNAN BETECKNING:**

**_______________________________________________________________________________**

**F232B**

**Jag kommer nu att läsa upp några olika påståenden och vill att du uppger i vilken grad**

**du instämmer i påståendet på en skala från 1 till 5, där 1 betyder instämmer inte alls**

**och 5 betyder instämmer helt och hållet.**

**PÅSTÅENDE:**

**Det är viktigt för mig att lära mig mycket om svensk kultur, traditioner och värderingar.**

**1 2 3 4 5**

**|---------------|---------------|---------------|---------------|**

**instämmer instämmer**

**inte alls helt och hållet**

**_______________________________________________________________________________**

**F232C**

**PÅSTÅENDE:**

**Jag försöker följa svenska vanor och traditioner.**

**1 2 3 4 5**

**|---------------|---------------|---------------|---------------|**

**instämmer instämmer**

**inte alls helt och hållet**

**_______________________________________________________________________________**

**F232D**

**PÅSTÅENDE:**

**Jag vill att mina barn uppfostras i enlighet med svenska traditioner.**

**1 2 3 4 5**

**|---------------|---------------|---------------|---------------|**

**instämmer instämmer**

**inte alls helt och hållet**

**_______________________________________________________________________________**

**F232E**

**PÅSTÅENDE:**

**Jag känner stolthet när svenskar är framgångsrika i t.ex. sport eller musik.**

**1 2 3 4 5**

**|---------------|---------------|---------------|---------------|**

**instämmer instämmer**

**inte alls helt och hållet**

**_______________________________________________________________________________**

**F232G**

**I vilken utsträckning känner du samhörighet med svensk kultur och svenska traditioner?**

**Svara på en skala från 1 till 5 där 1 betyder ingen samhörighet alls och**

**5 betyder stor samhörighet.**

**1 2 3 4 5**

**|---------------|---------------|---------------|---------------|**

**ingen stor**

**samhörighet samhörighet**

**_______________________________________________________________________________**

**F233B**

**Nu följer fler påståenden och jag vill att du uppger i vilken grad du instämmer**

**i påståendet på en skala från 1 till 5, där 1 betyder instämmer inte alls och**

**5 betyder instämmer helt och hållet.**

**PÅSTÅENDE:**

**Det är viktigt för mig att lära mig mycket om mina föräldrars hemlands**

**kultur, traditioner och värderingar.**

**1 2 3 4 5**

**|---------------|---------------|---------------|---------------|**

**instämmer instämmer**

**inte alls helt och hållet**

**_______________________________________________________________________________**

**F233C**

**PÅSTÅENDE:**

**Jag försöker följa vanor och traditioner som är vanliga i mina föräldrars hemland.**

**1 2 3 4 5**

**|---------------|---------------|---------------|---------------|**

**instämmer instämmer**

**inte alls helt och hållet**

**_______________________________________________________________________________**

**F233D**

**PÅSTÅENDE:**

**Jag vill att mina barn uppfostras i enlighet med traditioner som är vanliga i**

**mina föräldrars hemland.**

**1 2 3 4 5**

**|---------------|---------------|---------------|---------------|**

**instämmer instämmer**

**inte alls helt och hållet**

**_______________________________________________________________________________**

**F233E**

**PÅSTÅENDE:**

**Jag känner stolthet när personer som kommer från samma land som mina föräldrar**

**är framgångsrika i t.ex. sport eller musik.**

**1 2 3 4 5**

**|---------------|---------------|---------------|---------------|**

**instämmer instämmer**

**inte alls helt och hållet**

**_______________________________________________________________________________**

**F233F**

**PÅSTÅENDE:**

**Det är viktigt för mig att gifta mig med någon från mina föräldrars hemland.**

**1 2 3 4 5**

**|---------------|---------------|---------------|---------------|**

**instämmer instämmer**

**inte alls helt och hållet**

**_______________________________________________________________________________**

**F233G**

**Sen vill jag också fråga i vilken grad du känner samhörighet med dina föräldrars**

**hemlands kultur och traditioner?**

**Svara på en skala från 1 till 5 där 1 betyder ingen samhörighet alls och**

**5 betyder stor samhörighet.**

**1 2 3 4 5**

**|---------------|---------------|---------------|---------------|**

**ingen stor**

**samhörighet samhörighet**

**_______________________________________________________________________________**

**F233BM**

**Nu följer fler påståenden och jag vill att du uppger i vilken grad du instämmer**

**i påståendet på en skala från 1 till 5, där 1 betyder instämmer inte alls och**

**5 betyder instämmer helt och hållet.**

**PÅSTÅENDE:**

**Det är viktigt för mig att lära mig mycket om min mammas hemlands**

**kultur, traditioner och värderingar. ~~~~~~~**

**1 2 3 4 5**

**|---------------|---------------|---------------|---------------|**

**instämmer instämmer**

**inte alls helt och hållet**

**_______________________________________________________________________________**

**F233BP**

**Nu följer fler påståenden och jag vill att du uppger i vilken grad du instämmer**

**i påståendet på en skala från 1 till 5, där 1 betyder instämmer inte alls och**

**5 betyder instämmer helt och hållet.**

**PÅSTÅENDE:**

**Det är viktigt för mig att lära mig mycket om min pappas hemlands**

**kultur, traditioner och värderingar. ~~~~~~**

**1 2 3 4 5**

**|---------------|---------------|---------------|---------------|**

**instämmer instämmer**

**inte alls helt och hållet**

**_______________________________________________________________________________**

**F233CM**

**PÅSTÅENDE:**

**Jag försöker följa vanor och traditioner som är vanliga i min mammas hemland.**

**~~~~~~~**

**1 2 3 4 5**

**|---------------|---------------|---------------|---------------|**

**instämmer instämmer**

**inte alls helt och hållet**

**_______________________________________________________________________________**

**F233CP**

**PÅSTÅENDE:**

**Jag försöker följa vanor och traditioner som är vanliga i min pappas hemland.**

**~~~~~~**

**1 2 3 4 5**

**|---------------|---------------|---------------|---------------|**

**instämmer instämmer**

**inte alls helt och hållet**

**_______________________________________________________________________________**

**F233DM**

**PÅSTÅENDE:**

**Jag vill att mina barn uppfostras i enlighet med traditioner som är vanliga i min mammas hemland.**

**~~~~~~~**

**1 2 3 4 5**

**|---------------|---------------|---------------|---------------|**

**instämmer instämmer**

**inte alls helt och hållet**

**_______________________________________________________________________________**

**F233DP**

**PÅSTÅENDE:**

**Jag vill att mina barn uppfostras i enlighet med traditioner som är vanliga i min pappas hemland.**

**~~~~~~**

**1 2 3 4 5**

**|---------------|---------------|---------------|---------------|**

**instämmer instämmer**

**inte alls helt och hållet**

**_______________________________________________________________________________**

**F233EM**

**PÅSTÅENDE:**

**Jag känner stolthet när personer som kommer från samma land som min mamma**

**är framgångsrika i t.ex. sport eller musik. ~~~~~~**

**1 2 3 4 5**

**|---------------|---------------|---------------|---------------|**

**instämmer instämmer**

**inte alls helt och hållet**

**_______________________________________________________________________________**

**F233EP**

**PÅSTÅENDE:**

**Jag känner stolthet när personer som kommer från samma land som min pappa**

**är framgångsrika i t.ex. sport eller musik. ~~~~~**

**1 2 3 4 5**

**|---------------|---------------|---------------|---------------|**

**instämmer instämmer**

**inte alls helt och hållet**

**_______________________________________________________________________________**

**F233FM**

**PÅSTÅENDE:**

**Det är viktigt för mig att gifta mig med någon från min mammas hemland.**

**~~~~~~~**

**1 2 3 4 5**

**|---------------|---------------|---------------|---------------|**

**instämmer instämmer**

**inte alls helt och hållet**

**_______________________________________________________________________________**

**F233FP**

**PÅSTÅENDE:**

**Det är viktigt för mig att gifta mig med någon från min pappas hemland.**

**~~~~~~**

**1 2 3 4 5**

**|---------------|---------------|---------------|---------------|**

**instämmer instämmer**

**inte alls helt och hållet**

**_______________________________________________________________________________**

**F233GM**

**Sen vill jag också fråga i vilken grad du känner samhörighet med din mammas**

**hemlands kultur och traditioner? ~~~~~~~**

**Svara på en skala från 1 till 5 där 1 betyder ingen samhörighet alls och**

**5 betyder stor samhörighet.**

**1 2 3 4 5**

**|---------------|---------------|---------------|---------------|**

**ingen stor**

**samhörighet samhörighet**

**_______________________________________________________________________________**

**F233GP**

**Sen vill jag också fråga i vilken grad du känner samhörighet med din pappas**

**hemlands kultur och traditioner? ~~~~~~**

**Svara på en skala från 1 till 5 där 1 betyder ingen samhörighet alls och**

**5 betyder stor samhörighet.**

**1 2 3 4 5**

**|---------------|---------------|---------------|---------------|**

**ingen stor**

**samhörighet samhörighet**

**_______________________________________________________________________________**

**F235**

**Hur ofta deltar du i religiösa aktiviteter i kyrkan, moskén, synagogan eller liknande?**

**LÄS UPP SVARSALTERNATIVEN!**

**1 Varje dag**

**2 Mer än en gång i veckan**

**3 Ungefär en gång i veckan**

**4 Mer än en gång i månaden**

**5 Mer sällan**

**6 Aldrig**

**_______________________________________________________________________________**

**F236A**

**Hur religiös skulle du vilja säga att du är?**

**1 Mycket religiös**

**2 Ganska religiös**

**3 Lite religiös**

**4 Inte alls religiös**

**_______________________________________________________________________________**

**F236B**

**Vilken religion tillhör du?**

**1 Protestantisk kristendom**

**2 Katolsk kristendom**

**3 Ortodox kristendom**

**4 Frikyrklig (inkl mormon, jehovas vittne etc)**

**5 Kristendom, ospecifierat**

**6 Islam, shia (muslim = islam)**

**7 Islam, sunni (muslim = islam)**

**8 Islam - ospecifierat (muslim = islam)**

**9 Judendom**

**10 Annan ...**

**_______________________________________________________________________________**

**F236BTXT**

**( Vilken religion tillhör du? )**

**ANGE ANNAN RELIGION:**

**_______________________________________________________________________________**

**F237**

**Deltar du regelbundet i någon organiserad aktivitet (där man träffas på bestämda tider**

**för att träna, öva eller diskutera något)?**

**1 Ja**

**2 Nej**

**_______________________________________________________________________________**

**F238**

**Hur många gånger i veckan deltar du i sådana aktiviteter (en normal vecka)?**

**ANTAL GÅNGER PER VECKA:**

**_______________________________________________________________________________**

**F239A**

**Röstade du i 2010 års val till riksdagen?**

**1 Ja**

**2 Nej**

**3 UP ej röstberättigad**

**_______________________________________________________________________________**

**F239B**

**Röstade du i 2010 års val till kommunfullmäktige?**

**1 Ja**

**2 Nej**

**_______________________________________________________________________________**

**F240A**

**Om det vore riksdagsval idag, skulle du då rösta i valet?**

**1 Ja**

**2 Nej**

**3 UP ej röstberättigad**

**_______________________________________________________________________________**

**F240B**

**Om det vore kommunalval idag, skulle du då rösta i valet?**

**1 Ja**

**2 Nej**

**_______________________________________________________________________________**

**F241**

**Ungefär hur många timmar tittar du på TV en vanlig dag? (Inklusive DVD och video.)**

**ANTAL TIMMAR PER DAG:**

**_______________________________________________________________________________**

**F281**

**Brukar du gå på bio?**

**1 Någon gång i veckan eller oftare**

**2 Någon eller några gånger i månaden**

**3 Någon eller några gånger i kvartalet**

**4 Mer sällan eller aldrig**

**_______________________________________________________________________________**

**F281B**

**Vilken är den bästa film du sett i hela ditt liv?**

**Om du inte kan komma på vilken du tycker är bäst,**

**nämn en film eller regissör som du verkligen gillar.**

**FILM ELLER REGISSÖR:**

**_______________________________________________________________________________**

**F282**

**Hur ofta brukar du gå på teater?**

**1 Någon gång i veckan eller oftare**

**2 Någon eller några gånger i månaden**

**3 Någon eller några gånger i kvartalet**

**4 Mer sällan eller aldrig**

**_______________________________________________________________________________**

**F283**

**Hur ofta brukar du gå på konserter?**

**1 Någon gång i veckan eller oftare**

**2 Någon eller några gånger i månaden**

**3 Någon eller några gånger i kvartalet**

**4 Mer sällan eller aldrig**

**_______________________________________________________________________________**

**F283B**

**Vilken är din favoritartist eller grupp?**

**Om du inte kan komma på vem du tycker är bäst,**

**nämn en artist eller grupp som du verkligen gillar?**

**ARTIST ELLER GRUPP:**

**_______________________________________________________________________________**

**F284**

**Hur ofta brukar du gå på museum?**

**1 Någon gång i veckan eller oftare**

**2 Någon eller några gånger i månaden**

**3 Någon eller några gånger i kvartalet**

**4 Mer sällan eller aldrig**

**_______________________________________________________________________________**

**F285**

**Hur ofta brukar du gå på bibliotek på din fritid?**

**1 Någon gång i veckan eller oftare**

**2 Någon eller några gånger i månaden**

**3 Någon eller några gånger i kvartalet**

**4 Mer sällan eller aldrig**

**_______________________________________________________________________________**

**F286**

**Brukar du gå som åskådare på fotboll, ishockey, handboll eller annan lagidrott?**

**1 Någon gång i veckan eller oftare**

**2 Någon eller några gånger i månaden**

**3 Någon eller några gånger i kvartalet**

**4 Mer sällan eller aldrig**

**_______________________________________________________________________________**

**F286B**

**Vilket landslag skulle du heja du på i nästa fotbolls-VM, om alla lag vore med?**

**ANGE NATIONSLAG:**

**_______________________________________________________________________________**

**F288**

**Hur ofta brukar du gå ut och äta på restaurang eller krog?**

**1 Någon gång i veckan eller oftare**

**2 Någon eller några gånger i månaden**

**3 Någon eller några gånger i kvartalet**

**4 Mer sällan eller aldrig**

**_______________________________________________________________________________**

**F288B**

**Vilken är din favoriträtt?**

**ANGE MATRÄTT:**

**_______________________________________________________________________________**

**F288C**

**Hur ofta brukar du gå på fest?**

**1 Någon gång i veckan eller oftare**

**2 Någon eller några gånger i månaden**

**3 Någon eller några gånger i kvartalet**

**4 Mer sällan eller aldrig**

**_______________________________________________________________________________**

**F289A**

**Hur ofta brukar läsa en bok som inte är en lärobok?**

**1 Någon gång i veckan eller oftare**

**2 Någon eller några gånger i månaden**

**3 Någon eller några gånger i kvartalet**

**4 Mer sällan eller aldrig**

**_______________________________________________________________________________**

**F289B**

**Vilket är den bästa bok du läst i hela ditt liv?**

**Om du inte kan komma på vilken du tycker är bäst,**

**nämn en bok eller författare som du verkligen gillar.**

**TITEL ELLER FÖRFATTARE:**

**_______________________________________________________________________________**

**F289D**

**Ungefär hur många böcker finns det hemma hos dig?**

**__________________________________________________________**

**TILL IVE: En normal bokhyllerad rymmer ca 40 böcker och**

**en full bokhylla ca 200 böcker.**

**__________________________________________________________**

**ANTAL BÖCKER:**

**_______________________________________________________________________________**

**F289E**

**Hur ofta brukar du spela spel på dator och/eller konsol?**

**1 Någon gång i veckan eller oftare**

**2 Någon eller några gånger i månaden**

**3 Någon eller några gånger i kvartalet**

**4 Mer sällan eller aldrig**

**_______________________________________________________________________________**

**F2810**

**Ungefär hur många personer skulle du säga att du har kontakt med en vanlig dag, i genomsnitt?**

**Inklusive alla som du hälsar på och småpratar med, oavsett om det är ansikte mot ansikte,**

**på telefon, via e-post eller på internet och oavsett om du känner personen eller ej.**

**__________________________________________________________________________**

**TILL IVE: Om UP har svårt att uppskatta, föreslå: "Är det ett titoal eller ett femtiotal?".**

**Om UP säger t.ex. "tjugo-trettio", ange ett medelvärd (i detta fall 25).**

**__________________________________________________________________________**

**ANTAL PERSONER:**

**_______________________________________________________________________________**

**F291**

**Nu skall jag, till sist i intervjun, läsa upp ett antal påståenden.**

**För varje påstående ska du svara hur väl det stämmer in på dig.**

**PÅSTÅENDE:**

**Jag har svårt att sitta still och koncentrera mig.**

**LÄS UPP SVARSALTERNATIVEN!**

**1 Stämmer inte alls in på dig**

**2 Stämmer ganska dåligt in på dig**

**3 Stämmer varken bra eller dåligt in på dig**

**4 Stämmer ganska bra in på dig**

**5 Stämmer in på dig**

**_______________________________________________________________________________**

**F292**

**PÅSTÅENDE:**

**Jag ställer sällan till bråk.**

**LÄS UPP SVARSALTERNATIVEN!**

**1 Stämmer inte alls in på dig**

**2 Stämmer ganska dåligt in på dig**

**3 Stämmer varken bra eller dåligt in på dig**

**4 Stämmer ganska bra in på dig**

**5 Stämmer in på dig**

**_______________________________________________________________________________**

**F293**

**PÅSTÅENDE:**

**Jag är för det mesta beredd att ta risker.**

**LÄS UPP SVARSALTERNATIVEN VID BEHOV!**

**1 Stämmer inte alls in på dig**

**2 Stämmer ganska dåligt in på dig**

**3 Stämmer varken bra eller dåligt in på dig**

**4 Stämmer ganska bra in på dig**

**5 Stämmer in på dig**

**_______________________________________________________________________________**

**F294**

**PÅSTÅENDE:**

**Jag är ofta spänd och nervös.**

**LÄS UPP SVARSALTERNATIVEN VID BEHOV!**

**1 Stämmer inte alls in på dig**

**2 Stämmer ganska dåligt in på dig**

**3 Stämmer varken bra eller dåligt in på dig**

**4 Stämmer ganska bra in på dig**

**5 Stämmer in på dig**

**_______________________________________________________________________________**

**F295**

**PÅSTÅENDE:**

**Jag känner många gånger att jag har lite inflytande över saker som händer mig.**

**LÄS UPP SVARSALTERNATIVEN VID BEHOV!**

**1 Stämmer inte alls in på dig**

**2 Stämmer ganska dåligt in på dig**

**3 Stämmer varken bra eller dåligt in på dig**

**4 Stämmer ganska bra in på dig**

**5 Stämmer in på dig**

**_______________________________________________________________________________**

**F296**

**PÅSTÅENDE:**

**Jag känner mig ofta ledsen och nere.**

**LÄS UPP SVARSALTERNATIVEN VID BEHOV!**

**1 Stämmer inte alls in på dig**

**2 Stämmer ganska dåligt in på dig**

**3 Stämmer varken bra eller dåligt in på dig**

**4 Stämmer ganska bra in på dig**

**5 Stämmer in på dig**

**_______________________________________________________________________________**

**F297**

**PÅSTÅENDE:**

**Jag orkar göra mycket.**

**LÄS UPP SVARSALTERNATIVEN VID BEHOV!**

**1 Stämmer inte alls in på dig**

**2 Stämmer ganska dåligt in på dig**

**3 Stämmer varken bra eller dåligt in på dig**

**4 Stämmer ganska bra in på dig**

**5 Stämmer in på dig**

**_______________________________________________________________________________**

**F298**

**PÅSTÅENDE:**

**Jag kan påverka hur min framtid blir.**

**LÄS UPP SVARSALTERNATIVEN VID BEHOV!**

**1 Stämmer inte alls in på dig**

**2 Stämmer ganska dåligt in på dig**

**3 Stämmer varken bra eller dåligt in på dig**

**4 Stämmer ganska bra in på dig**

**5 Stämmer in på dig**

**_______________________________________________________________________________**

**F299**

**PÅSTÅENDE:**

**Jag tycker det är viktigt för mig att bilda familj.**

**LÄS UPP SVARSALTERNATIVEN VID BEHOV!**

**1 Stämmer inte alls in på dig**

**2 Stämmer ganska dåligt in på dig**

**3 Stämmer varken bra eller dåligt in på dig**

**4 Stämmer ganska bra in på dig**

**5 Stämmer in på dig**

**_______________________________________________________________________________**

**F2910**

**PÅSTÅENDE:**

**Jag tycker det är viktigt för mig att få gå på universitet eller högskola.**

**LÄS UPP SVARSALTERNATIVEN VID BEHOV!**

**1 Stämmer inte alls in på dig**

**2 Stämmer ganska dåligt in på dig**

**3 Stämmer varken bra eller dåligt in på dig**

**4 Stämmer ganska bra in på dig**

**5 Stämmer in på dig**

**_______________________________________________________________________________**

**F2912**

**PÅSTÅENDE:**

**Sett på det hela är jag lycklig.**

**LÄS UPP SVARSALTERNATIVEN VID BEHOV!**

**1 Stämmer inte alls in på dig**

**2 Stämmer ganska dåligt in på dig**

**3 Stämmer varken bra eller dåligt in på dig**

**4 Stämmer ganska bra in på dig**

**5 Stämmer in på dig**

**_______________________________________________________________________________**

**F2913**

**PÅSTÅENDE:**

**Jag känner mig ofta ensam.**

**LÄS UPP SVARSALTERNATIVEN VID BEHOV!**

**1 Stämmer inte alls in på dig**

**2 Stämmer ganska dåligt in på dig**

**3 Stämmer varken bra eller dåligt in på dig**

**4 Stämmer ganska bra in på dig**

**5 Stämmer in på dig**

**_______________________________________________________________________________**

**F2914**

**PÅSTÅENDE:**

**Jag försöker undvika risker, och tar det säkra före det osäkra.**

**LÄS UPP SVARSALTERNATIVEN VID BEHOV!**

**1 Stämmer inte alls in på dig**

**2 Stämmer ganska dåligt in på dig**

**3 Stämmer varken bra eller dåligt in på dig**

**4 Stämmer ganska bra in på dig**

**5 Stämmer in på dig**

**_______________________________________________________________________________**

**F2915**

**PÅSTÅENDE:**

**Det är viktigt för mig att få ett arbete där jag tjänar mycket pengar.**

**LÄS UPP SVARSALTERNATIVEN VID BEHOV!**

**1 Stämmer inte alls in på dig**

**2 Stämmer ganska dåligt in på dig**

**3 Stämmer varken bra eller dåligt in på dig**

**4 Stämmer ganska bra in på dig**

**5 Stämmer in på dig**

**_______________________________________________________________________________**

**F2916**

**PÅSTÅENDE:**

**Jag anser att bli framgångsrik är ett resultat av hårt arbete - tur har lite eller inget att göra med det.**

**LÄS UPP SVARSALTERNATIVEN VID BEHOV!**

**1 Stämmer inte alls in på dig**

**2 Stämmer ganska dåligt in på dig**

**3 Stämmer varken bra eller dåligt in på dig**

**4 Stämmer ganska bra in på dig**

**5 Stämmer in på dig**

**_______________________________________________________________________________**

**F2917**

**PÅSTÅENDE:**

**Jag är för det mesta nöjd med mig själv.**

**LÄS UPP SVARSALTERNATIVEN VID BEHOV!**

**1 Stämmer inte alls in på dig**

**2 Stämmer ganska dåligt in på dig**

**3 Stämmer varken bra eller dåligt in på dig**

**4 Stämmer ganska bra in på dig**

**5 Stämmer in på dig**

**_______________________________________________________________________________**

**F2918**

**PÅSTÅENDE:**

**Jag har bra självförtroende.**

**LÄS UPP SVARSALTERNATIVEN VID BEHOV!**

**1 Stämmer inte alls in på dig**

**2 Stämmer ganska dåligt in på dig**

**3 Stämmer varken bra eller dåligt in på dig**

**4 Stämmer ganska bra in på dig**

**5 Stämmer in på dig**

**_______________________________________________________________________________**

**F2919**

**PÅSTÅENDE:**

**Jag är ofta sur och irriterad.**

**LÄS UPP SVARSALTERNATIVEN VID BEHOV!**

**1 Stämmer inte alls in på dig**

**2 Stämmer ganska dåligt in på dig**

**3 Stämmer varken bra eller dåligt in på dig**

**4 Stämmer ganska bra in på dig**

**5 Stämmer in på dig**

**_______________________________________________________________________________**

**F2920**

**PÅSTÅENDE:**

**Jag vågar säga vad jag tycker.**

**LÄS UPP SVARSALTERNATIVEN VID BEHOV!**

**1 Stämmer inte alls in på dig**

**2 Stämmer ganska dåligt in på dig**

**3 Stämmer varken bra eller dåligt in på dig**

**4 Stämmer ganska bra in på dig**

**5 Stämmer in på dig**

**_______________________________________________________________________________**

**F2921**

**PÅSTÅENDE:**

**Jag är inflytelserik.**

**LÄS UPP SVARSALTERNATIVEN VID BEHOV!**

**1 Stämmer inte alls in på dig**

**2 Stämmer ganska dåligt in på dig**

**3 Stämmer varken bra eller dåligt in på dig**

**4 Stämmer ganska bra in på dig**

**5 Stämmer in på dig**

**_______________________________________________________________________________**

**F2922**

**PÅSTÅENDE:**

**Jag kan styra över mitt liv.**

**LÄS UPP SVARSALTERNATIVEN VID BEHOV!**

**1 Stämmer inte alls in på dig**

**2 Stämmer ganska dåligt in på dig**

**3 Stämmer varken bra eller dåligt in på dig**

**4 Stämmer ganska bra in på dig**

**5 Stämmer in på dig**

**_______________________________________________________________________________**

**F2923**

**PÅSTÅENDE:**

**Jag är nöjd med mitt utseende.**

**LÄS UPP SVARSALTERNATIVEN VID BEHOV!**

**1 Stämmer inte alls in på dig**

**2 Stämmer ganska dåligt in på dig**

**3 Stämmer varken bra eller dåligt in på dig**

**4 Stämmer ganska bra in på dig**

**5 Stämmer in på dig**

**_______________________________________________________________________________**

**F2924**

**PÅSTÅENDE:**

**Jag gillar att bestämma.**

**LÄS UPP SVARSALTERNATIVEN VID BEHOV!**

**1 Stämmer inte alls in på dig**

**2 Stämmer ganska dåligt in på dig**

**3 Stämmer varken bra eller dåligt in på dig**

**4 Stämmer ganska bra in på dig**

**5 Stämmer in på dig**

**_______________________________________________________________________________**

**F2925**

**PÅSTÅENDE:**

**Jag tror att jag kommer att få det bra i framtiden.**

**LÄS UPP SVARSALTERNATIVEN VID BEHOV!**

**1 Stämmer inte alls in på dig**

**2 Stämmer ganska dåligt in på dig**

**3 Stämmer varken bra eller dåligt in på dig**

**4 Stämmer ganska bra in på dig**

**5 Stämmer in på dig**

**_______________________________________________________________________________**

**F2926**

**PÅSTÅENDE:**

**Jag har inga bekymmer.**

**LÄS UPP SVARSALTERNATIVEN VID BEHOV!**

**1 Stämmer inte alls in på dig**

**2 Stämmer ganska dåligt in på dig**

**3 Stämmer varken bra eller dåligt in på dig**

**4 Stämmer ganska bra in på dig**

**5 Stämmer in på dig**

**_______________________________________________________________________________**

**F2927**

**PÅSTÅENDE:**

**När jag planerar är jag säker på att jag kan realisera mina planer.**

**LÄS UPP SVARSALTERNATIVEN VID BEHOV!**

**1 Stämmer inte alls in på dig**

**2 Stämmer ganska dåligt in på dig**

**3 Stämmer varken bra eller dåligt in på dig**

**4 Stämmer ganska bra in på dig**

**5 Stämmer in på dig**

**_______________________________________________________________________________**

**F2928**

**PÅSTÅENDE:**

**Jag blir väldigt lätt arg.**

**LÄS UPP SVARSALTERNATIVEN VID BEHOV!**

**1 Stämmer inte alls in på dig**

**2 Stämmer ganska dåligt in på dig**

**3 Stämmer varken bra eller dåligt in på dig**

**4 Stämmer ganska bra in på dig**

**5 Stämmer in på dig**

**_______________________________________________________________________________**

**F2929**

**PÅSTÅENDE:**

**Jag anser att vad som händer mig beror av mina egna handlingar.**

**LÄS UPP SVARSALTERNATIVEN VID BEHOV!**

**1 Stämmer inte alls in på dig**

**2 Stämmer ganska dåligt in på dig**

**3 Stämmer varken bra eller dåligt in på dig**

**4 Stämmer ganska bra in på dig**

**5 Stämmer in på dig**

**_______________________________________________________________________________**

**F2930**

**PÅSTÅENDE:**

**Jag skulle hellre välja att få 1000 kr idag än att få 2000 kr om ett år.**

**LÄS UPP SVARSALTERNATIVEN VID BEHOV!**

**1 Stämmer inte alls in på dig**

**2 Stämmer ganska dåligt in på dig**

**3 Stämmer varken bra eller dåligt in på dig**

**4 Stämmer ganska bra in på dig**

**5 Stämmer in på dig**

**_______________________________________________________________________________**

**F2931**

**PÅSTÅENDE:**

**Jag önskar att jag hade mer självkontroll.**

**LÄS UPP SVARSALTERNATIVEN VID BEHOV!**

**1 Stämmer inte alls in på dig**

**2 Stämmer ganska dåligt in på dig**

**3 Stämmer varken bra eller dåligt in på dig**

**4 Stämmer ganska bra in på dig**

**5 Stämmer in på dig**

**_______________________________________________________________________________**

**F2932**

**PÅSTÅENDE:**

**Jag gör ibland saker utan att tänka igenom alternativen.**

**LÄS UPP SVARSALTERNATIVEN VID BEHOV!**

**1 Stämmer inte alls in på dig**

**2 Stämmer ganska dåligt in på dig**

**3 Stämmer varken bra eller dåligt in på dig**

**4 Stämmer ganska bra in på dig**

**5 Stämmer in på dig**

**_______________________________________________________________________________**

**F30B**

**Det var alla frågor jag hade.**

**Forskargruppen vid Stockholms universitet har planer på att fortsätta**

**denna studie om unga människors livssituation.**

**Därför återkommer forskarna gärna till de som medverkat i undersökningen**

**om några år för att höra om och hur situationen förändrats.**

**Kan du tänka dig, att om några år, ta dig tid för en liknande intervju och**

**därigenom bidra med värdefull kunskap till forskningen om hur unga**

**människor lättare ska kunna etablera sig på arbetsmarknaden?**

**Även om du nu kan tänka dig att delta och tackar ja till det - så betyder det**

**inte att du förbinder dig till att delta.**

**Om forskningsstudien upprepas, och SCB ringer upp om några år, så tar du**

**först då ställning till om du vill medverka till intervju eller ej.**

**_____________________________________________________________**

**TILL IVE: Mer information finns under F4 (Visa hjälp).**

**_____________________________________________________________**

**1 JA, KAN TÄNKA SIG ATT DELTA**

**2 NEJ, VILL EJ DELTA**

**_______________________________________________________________________________**

**F30BB**

**I syfte att kunna kontakta dig för en eventuellt framtida studie, skulle SCB behöva spara kontaktuppgifter dvs ditt personnummer för att senare kunna återkontakta dig - dessa uppgifter lämnas inte ut till forskarna på Stockholms universitet, eller överhuvudtaget till någon.**

**Uppgifterna sparas vid SCB endast för syftet att om något år kunna återkontakta dig - för att du då ska kunna ta ställning till om du vill medverka till en uppföljningsintervju.**

**Uppgifterna skyddas enligt 24 kap. 8§ offentlighets- och sekretesslagen (2009:400) samt personuppgiftslagen (1998:204).**

**Kan vi spara dessa kontaktuppgifter för att kunna återkontakta dig om något år, för en eventuell uppföljningsintervju?**

**1 JA, UPPGIFTERNA KAN SPARAS**

**2 NEJ, VILL INTE ATT UPPGIFTERNA SPARAS**

**_______________________________________________________________________________**

**TACK**

**Det var alla frågor jag hade. Varmt tack för din värdefulla medverkan!**

**______________________________________________________________**

**TILL IVE: Avsluta samtalet med UP och fortsätt därefter till IVE-frågor.**

**______________________________________________________________**

**TRYCK 1 OCH ENTER.**

**_______________________________________________________________________________**

**F301**

**FRÅGA TILL INTERVJUAREN:**

**Hur upplever du UP:s svenska?**

**1 Helt flytande och korrekt svenska**

**2 Vissa icke-korrekta ordföljder**

**3 UP gör sig förstådd, men har stapplande språk och/eller bryter kraftigt**

**4 Mycket dålig framställningsförmåga, svår att förstå**

**5 Kan inte alls tala svenska**

**_______________________________________________________________________________**

**F302**

**FRÅGA TILL INTERVJUAREN:**

**Hur bedömer du tillförlitligheten i denna intervju?**

**1 Tillfredsställande**

**2 Inte helt tillfredsställande**

**3 Bristfällig**

**_______________________________________________________________________________**

**F303**

**FRÅGA TILL INTERVJUAREN:**

**Vilken / vilka omständigheter gav dig skäl att misstänka att tillförlitligheten inte är**

**helt tillfredsställande eller bristfällig?**

**BESKRIV (SAMMANFATTA):**

**_______________________________________________________________________________**

**F304**

**FRÅGA TILL INTERVJUAREN:**

**Finns det några särskilda områden i formuläret där tillförlitligheten verkar**

**särskilt låg i denna intervju?**

**____________________________________________________________**

**TILL IVE: Om inga särskilda områden, skriv NEJ i klartext.**

**____________________________________________________________**

**BESKRIV (SAMMANFATTA):**

**_______________________________________________________________________________**

**SLUT**

**FRÅGA TILL INTERVJUAREN:**

**Är UP bosatt eller arbetar utomlands?**

**______________________________________________**

**TILL IVE: Om ja, tryck F2 och anteckna vilket land.**

**______________________________________________**

**1 Ja**

**2 Nej**

**F8 = VET EJ**

**_______________________________________________________________________________**
