# Supplementary material for: School’s out forever? Heavy metal preferences and higher education
Source: PLoS One. 2019 Mar 19;14(3):e0213716. doi: 10.1371/journal.pone.0213716 (PMC6424403; doi:10.1371/journal.pone.0213716)
Supplement: S2 File — The survey machine translated to English. (DOCX) [file pone.0213716.s002.docx]

**MEDLYSS2**

**Visible on the screen!**

**NOTE! This issue must be the first consideration in all forms.**

**For documentation of the form, browse to question one.**

**2 = Closing of the recording**

**______________________________________________________________________________**

**TO FORM DESIGNER:**

**If the value 2 at some point during the interview that recording will pause.**

**(Calculate MEDLYSS2 = 2 if MEDLYSS1 = 1; *** Turn the recording)**

**______________________________________________________________________________**

**_______________________________________________________________________________**

**MEDLYSS3**

**Visible on the screen!**

**NOTE! This question must be that second question in all forms.**

**1 Yes, it is OK (REC)**

**2 No, do not agree (NO RECORDING)**

**3 Decline interview completely after question about recording**

**4 Indirect interview (NOT RECORDING)**

**5 IVE has not TRIO**

**_________________________________________________________________________________**

**TO FORM DESIGNER:**

**Stores answers from MEDLYSS1. Values ​​are 1 - 3, 5 allocated from MEDLYSS1 and value 4 from IDKOLL.**

**_________________________________________________________________________________**

**_______________________________________________________________________________**

**medly**

**Visible on the screen!**

**NOTE! This question must be that third question in any form.**

**1 MEDLYSS1 shown but no recording is performed**

**2 MEDLYSS1 displayed and recording is done at consent**

**______________________________________________________________________________**

**TO FORM DESIGNER:**

**Stores value from bakgrundsvariabeln implemented. The value assigned to the IDKOLL.**

**______________________________________________________________________________**

**_______________________________________________________________________________**

**MEDLYINFO**

**Visible on the screen!**

**To call screening off to work, the form must include the following five questions:**

**MEDLYSS1 The issue will come up on bakgrundsvariabeln FORS = '01', '02'.**

**The issue may be one of the following values**

**1 = Consent for recording**

**2 = Not consent for recording**

**3 = Decline interview**

**4 = INDIRECT INTERVIEW. The question of consent is when ever and the value 4 is assigned.**

**5 = IVE has not TRIO**

**MEDLYSS2 Dummy question. It gets the value 2 when the recording should be turned off.**

**If it has no value, going on record until the call is disconnected.**

**MEDLYSS3 Dummy question. Here are stored the response from MEDLYSS1. Value**

**MEDLYSS4 Dummy question. The question appears on MEDLYSS1 = 3rd**

**The interviewer encouraged to anbryta interview and coding loss.**

**(Result 60 with comment recording.)**

**medly Dummy question. Stores the value of bakgrundsvariabeln implemented.**

**1 of FORS = '01'**

**2 on FORS = '02'**

**and ask who Sets with whom the interview takes place, that is, whether it is direct or indirect interview.**

**Recording will take place only at the direct interview**

**_______________________________________________________________________________**

**ONE**

**Visible on the screen!**

**Investigation: Social capital, Wave 2**

**Study leader: Malin Forsberg**

**Production manager: Emma Agerberg**

**Form Designer: Slavica Dimic Kozarac**

**Duration: about xx h**

**Field work: 2013 Week 2 -**

**-------------------------------------------------- -------------------------------------------------- ---------------------------**

**2012-10-30 SDK The form began.**

**2013-01-14 SDK Production version created: Soc_Kap_vag2.**

**2013-01-16 SDK New prod.version created.**

**New answers salt added in the case ended.**

**2013-01-22 SDK New prod.version created. New reply length in question F236B.**

**-------------------------------------------------- -------------------------------------------------- ---------------------------**

**_______________________________________________________________________________**

**START DATE**

**Visible on the screen!**

**Here are stored the date (YYYYMMDD) when the interview started.**

**The date sättsi matter who.**

**_______________________________________________________________________________**

**COMPLETION**

**Visible on the screen!**

**Here are stored the date (YYYYMMDD) when the interview started.**

**The date is set in the case ended.**

**_______________________________________________________________________________**

**START TIME**

**Visible on the screen!**

**Here are stored the interview start time (HHMMSS).**

**The time put into question WHO.**

**_______________________________________________________________________________**

**END**

**Visible on the screen!**

**Here are stored the interview end time (HHMMSS).**

**The time is concerned END.**

**_______________________________________________________________________________**

**CONE**

**Visible on the screen!**

**Here are stored the person's gender selection.**

**1 = WOMEN**

**2 = MAN**

**_______________________________________________________________________________**

**T1**

**ISSUE ONLY USED IN PRACTICE FORM!**

**Enter the DP's full name (max 35 characters):**

**_______________________________________________________________________________**

**T2**

**ISSUE ONLY USED IN PRACTICE FORM!**

**Enter the DP's birth (YYYY):**

**_______________________________________________________________________________**

**T3**

**ISSUE ONLY USED IN PRACTICE FORM!**

**Enter the DP's birth month with two digits (MM):**

**_______________________________________________________________________________**

**T4**

**ISSUE ONLY USED IN PRACTICE FORM!**

**Enter the DP's birthday with two digits (DD):**

**_______________________________________________________________________________**

**T5**

**ISSUE ONLY USED IN PRACTICE FORM!**

**Enter the DP's sex:**

**Male 1**

**2 Female**

**_______________________________________________________________________________**

**T6**

**ISSUE ONLY USED IN PRACTICE FORM!**

**Was there any interview with UP in wave 1 (three years ago)?**

**1 Yes**

**No. 2**

**_______________________________________________________________________________**

**T7**

**ISSUE ONLY USED IN PRACTICE FORM!**

**Enter the DP's ethnic background:**

**1 Yugoslavian**

**2 Iranian**

**3 Swedish**

**_______________________________________________________________________________**

**WHO**

**WHO interviewed?**

**1 UP - THE SWEDISH**

**2 UP - THE LANGUAGE (Without interpreter)**

**3 ELSE**

**_______________________________________________________________________________**

**IDKOLL**

**First I want to assure me that the interview takes place with the right person.**

**Is it true you were born on FodDagTxt << TEXT >> << TEXT FodManadTxt >> << TEXT >> FodAr?**

**1 YES**

**NO ---> STOP INTERVIEW**

**_________________________________________________________________________**

**TO IVE: NOTE! The response should be spoken only where explicitly stated.**

**WinDATI's standard response can be set on most issues: F8 = Do not know**

**F9 = Do not answer**

**NOTE! F3 usually can not be used in this form, use instead F7.**

**The symbol :: indicates that there are instructions for the interviewer during the F4 key (Display Help).**

**_________________________________________________________________________**

**_______________________________________________________________________________**

**MEDLYSS1**

**________________________________________________________________________**

**| |**

**| THE INTERVIEWER: |**

**| |**

**| If you have not TRIO installed on your computer, the issue is not set to UP. |**

**| |**

**| Press 5 and ENTER to continue. |**

**| _______________________________________________________________________ |**

**We are constantly working to improve the questions and interview techniques in our surveys**

**and therefore we want, if you agree, to record this interview.**

**The recording is protected by the Official Secrets Act and the Data Protection Act. We who work**

**the investigation of professional and recording is destroyed within 3 months.**

**Is it good for you that we are recording this interview?**

**_________________________________________________________________________**

**TO IVE: Statistics Sweden's statistical activities protected data on individuals, companies**

**and other organizations of privacy. Statistical confidentiality also applies to the**

**other authorities with specific statistical activities. (For details, press F4.)**

**_________________________________________________________________________**

**1 YES, it goes well**

**2 NO, NOT AGREE**

**Three declines INTERVIEW FULLY DEMAND FOR RECORDING**

**_______________________________________________________________________________**

**MEDLYSS4**

**STOP THE INTERVIEW!**

**_________________________________________________________________**

**TO IVE: Clear Koda with result code 60 and**

**write "Recording", commenting on the result code.**

**_________________________________________________________________**

**_______________________________________________________________________________**

**F10A**

**I was going to start by asking if you were born in Sweden or in another country?**

**1 Sweden**

**2 Other country**

**_______________________________________________________________________________**

**F10B**

**Is one of your parents was born abroad?**

**1 Yes, Dad**

**2 Yes, mom**

**3 Yes, both**

**4 No, none of them**

**_______________________________________________________________________________**

**F10c**

**If your parents were born in the same country?**

**1 Yes, the same country**

**2 No, different countries**

**_______________________________________________________________________________**

**F11A**

**During the last twelve months, have you mainly been living alone or with someone else?**

**Alone 1**

**2 together with any**

**_______________________________________________________________________________**

**F11BS1**

**Visible on the screen!**

**Here are stored responses from F11B:**

**Parents 1**

**_______________________________________________________________________________**

**F11BS2**

**Visible on the screen!**

**Here are stored responses from F11B:**

**2 Siblings**

**_______________________________________________________________________________**

**F11BS3**

**Visible on the screen!**

**Here are stored responses from F11B:**

**3 Relatives who are not parents or siblings**

**_______________________________________________________________________________**

**F11BS4**

**Visible on the screen!**

**Here are stored responses from F11B:**

**4 Partner**

**_______________________________________________________________________________**

**F11BS5**

**Visible on the screen!**

**Here are stored responses from F11B:**

**5 Groups**

**_______________________________________________________________________________**

**F11B**

**During the last twelve months, you have mainly lived with**

**friend, sibling, partner or with your parents?**

**SEVERAL ANSWERS CAN BE GIVEN. Press space between the answers.**

**Parents 1**

**2 Siblings**

**3 Relatives who are not parents or siblings**

**4 Partner (incl boyfriend / girlfriend, husband / wife, partner)**

**5 Groups**

**_______________________________________________________________________________**

**F12A**

**What type of accommodation do you live in?**

**1 LEASEHOLDING for apartment / house / terraced**

**2 Sublease for apartment / house / terraced**

**3 condominium**

**4 Villa**

**5 Duplex**

**6 Dormitory / -Apartment**

**Other 7**

**_______________________________________________________________________________**

**F12B**

**How long do << TEXT 'you' if F11A = 1 >> << TEXT 'you' if F11A = 1 >> live there?**

**____________________________________________________**

**TO IVE: Enter the answer in months of occupancy.**

**If less than 1 month, enter 0th**

**____________________________________________________**

**NUMBER OF MONTHS:**

**_______________________________________________________________________________**

**F12C**

**How did you get in your accommodation?**

**1 Purchased by brokers**

**2 Purchased black**

**Tip 3 via contacts**

**4 the housing**

**5 via the relay service, for example on the Internet**

**6 By ads**

**7 through membership in cooperative**

**8 via the social office**

**9 via the student body / nation**

**10 Others**

**_______________________________________________________________________________**

**F21**

**How many siblings do you have?**

**Enter the number of:**

**_______________________________________________________________________________**

**F22**

**Have you fixed the company or are you married?**

**1 No, neither partner / the company or are married**

**2 Yes, partner / the company (including boy / girlfriend or partner)**

**3 Yes, married**

**_______________________________________________________________________________**

**F23**

**How many own children do you have?**

**If there are no children of their own, SET 0th**

**NUMBER OF CHILDREN:**

**_______________________________________________________________________________**

**F24A**

**Is your biological child?**

**1 Yes**

**No. 2**

**_______________________________________________________________________________**

**Q24b**

**How many are your biological children?**

**If no biological children, SET 0th**

**NUMBER OF BIOLOGICAL CHILDREN:**

**_______________________________________________________________________________**

**F31**

**Has your mother or father studied at a university in Sweden?**

**1 Yes, Dad**

**2 Yes, mom**

**3 Yes, both**

**4 No, none of them**

**_______________________________________________________________________________**

**F32**

**Has your mother or father studied at a university in another country?**

**1 Yes, Dad**

**2 Yes, mom**

**3 Yes, both**

**4 No, none of them**

**_______________________________________________________________________________**

**F33OPA1**

**What did your father (stepfather) had the main occupation or employment**

**During his time in Sweden until today?**

**~~~~~~~**

**________________________________________________________________________**

**TO IVE: If the DP's father (stepfather) had a job that is not in the list,**

**e.g. unemployed, students, sick leave, etc. economic independence**

**enter 1 and DP's answers in plain text, rather than "forcing" the answer in the list.**

**________________________________________________________________________**

**1 = PROFESSION / JOBS ARE NOT LISTED**

**PROFESSION / OCCUPATION:**

**_______________________________________________________________________________**

**F33OPA1TXT**

**(What is your father (stepfather) had the main occupation or employment**

**During his time in Sweden until today? )**

**~~~~~~~**

**___________________________________________________________________________**

**TO IVE: Try to find out the following**

**- Main duties**

**- Industry**

**- Operation (as home care for the elderly, the construction of wall and plaster)**

**- The qualification level (eg management work, military work, workers and employees)**

**___________________________________________________________________________**

**SET CAREER / EMPLOYMENT IN PLAIN LANGUAGE:**

**_______________________________________________________________________________**

**F33OPA2**

**What did your father (stepfather) main occupation or employment**

**before moving to Sweden?**

**~~~~**

**________________________________________________________________________**

**TO IVE: If the DP's father (stepfather) had a job that is not in the list,**

**e.g. unemployed, students, sick leave, etc. economic independence**

**enter 1 and DP's answers in plain text, rather than "forcing" the answer in the list.**

**________________________________________________________________________**

**1 = PROFESSION / JOBS ARE NOT LISTED**

**PROFESSION / OCCUPATION:**

**_______________________________________________________________________________**

**F33OPA2TXT**

**(What was your father (stepfather) main occupation or employment**

**before moving to Sweden? )**

**~~~~**

**___________________________________________________________________________**

**TO IVE: Try to find out the following**

**- Main duties**

**- Industry**

**- Operation (as home care for the elderly, the construction of wall and plaster)**

**- The qualification level (eg management work, military work, workers and employees)**

**___________________________________________________________________________**

**SET CAREER / EMPLOYMENT IN PLAIN LANGUAGE:**

**_______________________________________________________________________________**

**F33OMA1**

**What is your mother (stepmother) had the main occupation or employment**

**During his time in Sweden until today?**

**~~~~~~~**

**__________________________________________________________________________**

**TO IVE: If the DP's mother (stepmother) had a job that is not in the list,**

**e.g. housewife, unemployed, students, sick leave, etc. economic independence**

**enter 1 and DP's answers in plain text, rather than "forcing" the answer in the list.**

**__________________________________________________________________________**

**1 = PROFESSION / JOBS ARE NOT LISTED**

**PROFESSION / OCCUPATION:**

**_______________________________________________________________________________**

**F33OMA1TXT**

**(What is your mother (stepmother) had the main occupation or employment**

**During his time in Sweden until today? )**

**~~~~~~~**

**___________________________________________________________________________**

**TO IVE: Try to find out the following**

**- Main duties**

**- Industry**

**- Operation (as home care for the elderly, the construction of wall and plaster)**

**- The qualification level (eg management work, military work, workers and employees)**

**___________________________________________________________________________**

**SET CAREER / EMPLOYMENT IN PLAIN LANGUAGE:**

**_______________________________________________________________________________**

**F33OMA2**

**What did your mother (stepmother) main occupation or employment**

**before moving to Sweden?**

**~~~~**

**__________________________________________________________________________**

**TO IVE: If the DP's mother (stepmother) had a job that is not in the list,**

**e.g. housewife, unemployed, students, sick leave, etc. economic independence**

**enter 1 and DP's answers in plain text, rather than "forcing" the answer in the list.**

**__________________________________________________________________________**

**1 = PROFESSION / JOBS ARE NOT LISTED**

**PROFESSION / OCCUPATION:**

**_______________________________________________________________________________**

**F33OMA2TXT**

**(What was your mother (stepmother) main occupation or employment**

**before moving to Sweden? )**

**~~~~**

**___________________________________________________________________________**

**TO IVE: Try to find out the following**

**- Main duties**

**- Industry**

**- Operation (as home care for the elderly, the construction of wall and plaster)**

**- The qualification level (eg management work, military work, workers and employees)**

**___________________________________________________________________________**

**SET CAREER / EMPLOYMENT IN PLAIN LANGUAGE:**

**_______________________________________________________________________________**

**F33**

**If you look back on your childhood, that is, until today, what has your father (stepfather)**

**had the principal occupation or employment?**

**________________________________________________________________________**

**TO IVE: If the DP's father (stepfather) had a job that is not in the list,**

**e.g. unemployed, students, sick leave, etc. economic independence**

**enter 1 and DP's answers in plain text, rather than "forcing" the answer in the list.**

**________________________________________________________________________**

**1 = PROFESSION / JOBS ARE NOT LISTED**

**PROFESSION / OCCUPATION:**

**_______________________________________________________________________________**

**F33TXT**

**(If you look back on your childhood, that is, until today, what has your father (stepfather)**

**had the principal occupation or employment? )**

**___________________________________________________________________________**

**TO IVE: Try to find out the following**

**- Main duties**

**- Industry**

**- Operation (as home care for the elderly, the construction of wall and plaster)**

**- The qualification level (eg management work, military work, workers and employees)**

**___________________________________________________________________________**

**SET CAREER / EMPLOYMENT IN PLAIN LANGUAGE:**

**_______________________________________________________________________________**

**F34**

**If you look back on your childhood, that is, until today, what is your mother (stepmother)**

**had the principal occupation or employment?**

**__________________________________________________________________________**

**TO IVE: If the DP's mother (stepmother) had a job that is not in the list,**

**e.g. housewife, unemployed, students, sick leave, etc. economic independence**

**enter 1 and DP's answers in plain text, rather than "forcing" the answer in the list.**

**__________________________________________________________________________**

**1 = PROFESSION / JOBS ARE NOT LISTED**

**PROFESSION / OCCUPATION:**

**_______________________________________________________________________________**

**F34TXT**

**(If you look back on your childhood, that is, until today, what is your mother (stepmother)**

**had the principal occupation or employment? )**

**___________________________________________________________________________**

**TO IVE: Try to find out the following**

**- Main duties**

**- Industry**

**- Operation (as home care for the elderly, the construction of wall and plaster)**

**- The qualification level (eg management work, military work, workers and employees)**

**___________________________________________________________________________**

**SET CAREER / EMPLOYMENT IN PLAIN LANGUAGE:**

**_______________________________________________________________________________**

**F41A**

**How would you describe your father's attitude toward religion?**

**Would describe him as ...**

**1 ... very religious,**

**2 ... quite religious,**

**3 ... not very religious, or**

**4 ... not religious at all?**

**_______________________________________________________________________________**

**F41B**

**What religion belong / belonged to him?**

**1 Protestant Christianity**

**2 Catholic Christianity**

**3 Orthodox Christianity**

**4 Frikyrklig (Incl Mormon, Witness etc.)**

**5 Kristendom, unspecified**

**6 Islam, Shia (Muslim = Islam)**

**7 Islam, Sunni (Muslim = Islam)**

**8 Islam - unspecified (Muslim = Islam)**

**9 Judaism**

**... 10 Other**

**_______________________________________________________________________________**

**F41BTXT**

**(What religion belong / belonged to him?)**

**ENTERING ANOTHER RELIGION:**

**_______________________________________________________________________________**

**F42A**

**How would you describe your mother's attitude to religion?**

**Would describe her as ...**

**READ OUT THE OPTIONS!**

**1 ... very religious,**

**2 ... quite religious,**

**3 ... not very religious, or**

**4 ... not religious at all?**

**_______________________________________________________________________________**

**F42B**

**What religion belong / belonged to her?**

**1 Protestant Christianity**

**2 Catholic Christianity**

**3 Orthodox Christianity**

**4 Frikyrklig (Incl Mormon, Witness etc.)**

**5 Kristendom, unspecified**

**6 Islam, Shia (Muslim = Islam)**

**7 Islam, Sunni (Muslim = Islam)**

**8 Islam - unspecified (Muslim = Islam)**

**9 Judaism**

**... 10 Other**

**_______________________________________________________________________________**

**F42BTXT**

**(What religion belong / belonged to her?)**

**ENTERING ANOTHER RELIGION:**

**_______________________________________________________________________________**

**F51A**

**<< TEXT 'Have you lived in a country other than Sweden for at least two years?' if UPUtlandsFodd = 'no' >> << TEXT 'Since you came to Sweden for the first time, you have lived in another country for at least two years?' if UPUtlandsFodd = 'yes' >>**

**1 Yes**

**No. 2**

**_______________________________________________________________________________**

**TAB1START**

**Visible on the screen!**

**Here begins the tableau: A stay abroad**

**_______________________________________________________________________________**

**F52A**

**<< TEXT 'If you have lived abroad in several countries and at several different times, so we take a stay abroad' on TAB1varv = 1 >> << TEXT 'In which country did you live then? 'Of TAB1varv> 1 >>**

**<< TEXT 'time. We begin with the first stay abroad 'on TAB1varv = 1 >> << TEXT' ... 'if TAB1varv = 1 >> << TEXT' (since you came to Sweden for the first time). ' if TAB1varv = 1 & UPUtlandsFodd = 'yes' >>**

**<< TEXT 'In which country did you live then? 'Of TAB1varv = 1 >>**

**__________________________________________________________________**

**TO IVE: Enter DP's spontaneous reply. Do not answer to get hit in the list,**

**but rather write the country in clear text in the next issue.**

**__________________________________________________________________**

**1 = COUNTRY ARE LISTED**

**COUNTRY:**

**_______________________________________________________________________________**

**F52ATXT**

**(In which country did you live then?)**

**ENTERING THE COUNTRY IN PLAIN LANGUAGE:**

**_______________________________________________________________________________**

**F52B1**

**STAY ABROAD: << TEXT TAB1varv >>**

**What year did you move there?**

**__________________________________________________________**

**TO IVE: Country: TEXT << Country name >>**

**__________________________________________________________**

**From the year:**

**_______________________________________________________________________________**

**F52B2**

**STAY ABROAD: << TEXT TAB1varv >>**

**What year did you move from there?**

**__________________________________________________________**

**TO IVE: Country: TEXT << Country name >>**

**__________________________________________________________**

**THROUGH THE YEARS:**

**_______________________________________________________________________________**

**F53**

**STAY ABROAD: << TEXT TAB1varv >>**

**Did you go to school there during that period?**

**______________________________________________________________________**

**TO IVE: Period: << TEXT FromAr >> - << TEXT >> Tomar in TEXT << Country name >>**

**______________________________________________________________________**

**1 Yes**

**No. 2**

**_______________________________________________________________________________**

**F54**

**STAY ABROAD: << TEXT TAB1varv >>**

**Did you work there during that period?**

**______________________________________________________________________**

**TO IVE: Period: << TEXT FromAr >> - << TEXT >> Tomar in TEXT << Country name >>**

**______________________________________________________________________**

**1 Yes**

**No. 2**

**_______________________________________________________________________________**

**TAB1EXT**

**If you have since lived in a country other than Sweden for at least two years?**

**1 Yes**

**No. 2**

**_______________________________________________________________________________**

**TAB1SLUT**

**Visible on the screen!**

**Here ends the tableau: A stay abroad**

**_______________________________________________________________________________**

**F55A**

**Have you visited << TEXT 'your parents' if MorUtlandsFodd = 'yes' & FarUtlandsFodd = 'yes' & ForaldrarSammaFodelseLand = 'yes' >> << TEXT 'your mother' of MorUtlandsFodd = 'yes' & (FarUtlandsFodd = 'no' | ForaldrarSammaFodelseLand = 'no') >> << TEXT 'and / or' if MorUtlandsFodd = 'yes' & FarUtlandsFodd = 'yes' & ForaldrarSammaFodelseLand = 'no' >> << TEXT 'your father' of FarUtlandsFodd = 'yes' & (MorUtlandsFodd = 'no' | ForaldrarSammaFodelseLand = 'no') >> birth during the last 5 years?**

**1 Yes**

**No. 2**

**_______________________________________________________________________________**

**F55B**

**How many times have you visited << TEXT 'your parents' if MorUtlandsFodd = 'yes' & FarUtlandsFodd = 'yes' & ForaldrarSammaFodelseLand = 'yes' >> << TEXT 'your mother' of MorUtlandsFodd = 'yes' & (FarUtlandsFodd = 'no' | ForaldrarSammaFodelseLand = 'no') >> << TEXT 'and / or' if MorUtlandsFodd = 'yes' & FarUtlandsFodd = 'yes' & ForaldrarSammaFodelseLand = 'no' >> << TEXT 'your father' of FarUtlandsFodd = 'yes' & (MorUtlandsFodd = 'no' | ForaldrarSammaFodelseLand = 'no') >> birth during the last 5 years?**

**ENTER NUMBER OF TIMES:**

**_______________________________________________________________________________**

**F56**

**How often are you in contact with family or friends in << TEXT 'your parents' if MorUtlandsFodd =' yes' & FarUtlandsFodd = 'yes' & ForaldrarSammaFodelseLand =' yes' >> << TEXT 'your mother' of MorUtlandsFodd = 'yes '& (FarUtlandsFodd =' no '| ForaldrarSammaFodelseLand =' no ') >> << TEXT' and / or 'if MorUtlandsFodd =' yes '& FarUtlandsFodd =' yes '& ForaldrarSammaFodelseLand =' no '>> << TEXT' your daddy 'of FarUtlandsFodd =' yes' & (MorUtlandsFodd = 'no' | ForaldrarSammaFodelseLand = 'no') >> birth?**

**(It may be by mail, phone, email, etc.).**

**READ OUT OPTIONS WHEN NEEDED!**

**1 At least weekly**

**2 At least once a month**

**3 At least twice a year**

**4 Rarely**

**5 Never**

**_______________________________________________________________________________**

**F57**

**Have you over the past two years sent money to someone in << TEXT 'your parents' if MorUtlandsFodd =' yes' & FarUtlandsFodd = 'yes' & ForaldrarSammaFodelseLand =' yes' >> << TEXT 'your mother' of MorUtlandsFodd = ' yes '& (FarUtlandsFodd =' no '| ForaldrarSammaFodelseLand =' no ') >> << TEXT' and / or 'if MorUtlandsFodd =' yes '& FarUtlandsFodd =' yes '& ForaldrarSammaFodelseLand =' no '>> << TEXT' your father 'of FarUtlandsFodd =' yes' & (MorUtlandsFodd = 'no' | ForaldrarSammaFodelseLand = 'no') >> birth?**

**1 Yes**

**No. 2**

**_______________________________________________________________________________**

**F61**

**What language do you mostly speak with your parents?**

**Swedish 1**

**2 Persian**

**3 Kurdish**

**4 Arabic**

**5 English**

**6 Albania**

**7 Serbo-Croatian (incl. Serb and Croatian)**

**8 Bosnian**

**9 Other language ...**

**_______________________________________________________________________________**

**F61TXT**

**(What language do you mostly speak with your parents?)**

**SET LANGUAGE:**

**_______________________________________________________________________________**

**F62**

**Do you also speak another language with your parents?**

**1 NO**

**<< TEXT 'Swedish 2 'To F61 = 1 >>**

**<< TEXT '3 Persian 'To F61 = 2 >>**

**<< TEXT '4 Kurdish 'To F61 = 3 >>**

**<< TEXT '5 Arabic 'To F61 = 4 >>**

**<< TEXT 'English 6 'To F61 = 5 >>**

**<< TEXT '7 Albania 'To F61 = 6 >>**

**<< TEXT '8 Serbo-Croatian (incl. Serb and Croatian) 'To F61 = 7 >>**

**<< TEXT '9 Bosnian 'To F61 = 8 >>**

**10 Other language ...**

**_______________________________________________________________________________**

**F62TXT**

**(Do you even speak another language with your parents?)**

**SET LANGUAGE:**

**_______________________________________________________________________________**

**F63**

**What language do you mostly speak with your friends?**

**Swedish 1**

**2 Persian**

**3 Kurdish**

**4 Arabic**

**5 English**

**6 Albania**

**7 Serbo-Croatian (incl. Serb and Croatian)**

**8 Bosnian**

**9 Other language ...**

**_______________________________________________________________________________**

**F63TXT**

**(What language do you mostly speak with your friends?)**

**SET LANGUAGE:**

**_______________________________________________________________________________**

**F64**

**Do you also speak another language with your friends?**

**1 NO**

**<< TEXT 'Swedish 2 'To F63 = 1 >>**

**<< TEXT '3 Persian 'To F63 = 2 >>**

**<< TEXT '4 Kurdish 'To F63 = 3 >>**

**<< TEXT '5 Arabic 'To F63 = 4 >>**

**<< TEXT 'English 6 'To F63 = 5 >>**

**<< TEXT '7 Albania 'To F63 = 6 >>**

**<< TEXT '8 Serbo-Croatian (incl. Serb and Croatian) 'To F63 = 7 >>**

**<< TEXT '9 Bosnian 'To F63 = 8 >>**

**10 Other language ...**

**_______________________________________________________________________________**

**F64TXT**

**(Do you even speak another language with your friends?)**

**SET LANGUAGE:**

**_______________________________________________________________________________**

**F7INTRO**

**Now comes a section that deals with your friends and acquaintances.**

**Think of five people you meet and interact with most frequently at your leisure.**

**Before I start with the questions, I need to write down their names.**

**Mention only people older than 10 years.**

**Suffice first name and the first letter of the surname. I need**

**names just because we both later in the interview to know which person is**

**I ask for.**

**__________________________________________________________________**

**TO IVE: It's about friends simply; but this may also parents,**

**siblings and other relatives to join.**

**Try to get UP to disclose all 5 people!**

**NOTE! If UP is not able or willing to provide any person at all, press F9.**

**__________________________________________________________________**

**Press 1 and ENTER to start Record the names.**

**_______________________________________________________________________________**

**TAB2START**

**Visible on the screen!**

**Here begins the tableau: Friends name**

**_______________________________________________________________________________**

**F7NAMN**

**PERSONAL << TEXT TAB2varv >>**

**<< TEXT 'Shall we start with the friend or the person that you meet the most. What's he? 'If TAB2varv = 1 >> << TEXT' What's the other person? 'If TAB2varv = 2 >> << TEXT' ... and the next person? 'Of TAB2varv> 2 >>**

**___________________________________________________________________**

**TO IVE: If the UP find it uncomfortable to disclose the person's real name,**

**ask for an "alias" instead.**

**___________________________________________________________________**

**<< TEXT 'if no more persons, FILL 0.' of TAB2varv> 1 >>**

**ENTER NAME (PERSON << TEXT >> TAB2varv):**

**_______________________________________________________________________________**

**TAB2SLUT**

**Visible on the screen!**

**Here ends the tableau: Friends name**

**_______________________________________________________________________________**

**TAB3START**

**Visible on the screen!**

**Here begins the tableau: Friends**

**_______________________________________________________________________________**

**F71**

**<< TEXT 'Then I start with a few questions about your friends, and I'm starting with' if TAB3varv = 1 >> << TEXT 'Let's go over to the' on TAB3varv> 1 >> << TEXT >> friends name.**

**Is it a man or a woman?**

**___________________________________________________________________**

**TO IVE: The question need not be about sex clearly evident from the name,**

**but confirm the answer, so that DPs know what you are signing.**

**___________________________________________________________________**

**Male 1**

**2 Female**

**_______________________________________________________________________________**

**F7RELATION**

**PERSONAL << TEXT TAB3varv >>: << TEXT >> friends name**

**Is << >> TEXT HanHon a sibling, << TEXT 'boyfriend' of VanKon = '1' >> << TEXT 'girlfriend' of VanKon = '2' >> << TEXT 'boyfriend / girlfriend' of VanKon = ' '>> or related to you in some other way?**

**1 Parent**

**2 Siblings**

**3 << TEXT 'boyfriend' of VanKon = '1' >> << TEXT 'girlfriend' of VanKon = '2' >> << TEXT 'boyfriend / girlfriend' of VanKon = '' >>**

**4 << TEXT 'make' on VanKon = '1' >> << TEXT 'Wife' of VanKon = '2' >> << TEXT 'husband / wife' of VanKon = '' >>**

**5 Cousin**

**6 another family**

**7 No, not relative or partner**

**_______________________________________________________________________________**

**F72**

**PERSONAL << TEXT TAB3varv >>: << TEXT >> friends name**

**Approximately how old is HanHon << TEXT >>?**

**_________________________________________________________________________**

**TO IVE: Enter the answer in the whole year. Ask DP to answer as precisely as possible.**

**If UP responds with range, enter mean, for example, "30-40 years," enter the 35th**

**_________________________________________________________________________**

**AGE (years):**

**_______________________________________________________________________________**

**F73**

**PERSONAL << TEXT TAB3varv >>: << TEXT >> friends name**

**Lives << TEXT >> HanHon in the same neighborhood as you?**

**CLARIFY IF NECESSARY: In residential, we mean the area that you**

**consider your neighborhood.**

**1 Yes**

**No. 2**

**_______________________________________________________________________________**

**F74**

**PERSONAL << TEXT TAB3varv >>: << TEXT >> friends name**

**Is << TEXT >> HanHon born in the same country as you?**

**1 Yes**

**No. 2**

**_______________________________________________________________________________**

**F75**

**PERSONAL << TEXT TAB3varv >>: << TEXT >> friends name**

**In which country is << TEXT >> HanHon born?**

**__________________________________________________________________**

**TO IVE: Enter DP's spontaneous reply. Do not answer to get hit in the list,**

**but rather write the country in clear text in the next issue.**

**__________________________________________________________________**

**1 = COUNTRY ARE LISTED**

**COUNTRY:**

**_______________________________________________________________________________**

**F75TXT**

**PERSONAL << TEXT TAB3varv >>: << TEXT >> friends name**

**(In which country is << TEXT >> HanHon born?)**

**ENTERING THE COUNTRY IN PLAIN LANGUAGE:**

**_______________________________________________________________________________**

**F75REGION**

**PERSONAL << TEXT TAB3varv >>: << TEXT >> friends name**

**Do you know of any region in the world << TEXT >> HanHon born?**

**READ OUT OPTIONS WHEN NEEDED!**

**No. 1**

**2 Yes, in the Nordic countries / Scandinavia**

**3 Yes, in Western Europe (except the Nordic countries)**

**4 Yes, in Eastern Europe (excluding Turkey)**

**5 Yes, in the Middle East (including Turkey)**

**6 Yes, in Asia (excluding the Middle East)**

**7 Yes, in Africa**

**8 Yes, in North America**

**9 Yes, in South America**

**10 Yes, but none of the above regions**

**_______________________________________________________________________________**

**F76A**

**PERSONAL << TEXT TAB3varv >>: << TEXT >> friends name**

**Is << TEXT >> His Her parents were born in Sweden?**

**1 Yes, both parents**

**2 No, only the mother (born in Sweden)**

**3 No, only the father (born in Sweden)**

**4 No, none of them**

**_______________________________________________________________________________**

**F76B**

**PERSONAL << TEXT TAB3varv >>: << TEXT >> friends name**

**In which country is << TEXT >> His Her mother born?**

**__________________________________________________________________**

**TO IVE: Enter DP's spontaneous reply. Do not answer to get hit in the list,**

**but rather write the country in clear text in the next issue.**

**__________________________________________________________________**

**1 = COUNTRY ARE LISTED**

**COUNTRY:**

**_______________________________________________________________________________**

**F76BTXT**

**PERSONAL << TEXT TAB3varv >>: << TEXT >> friends name**

**(In which country is << TEXT >> His Her mother born?)**

**ENTERING THE COUNTRY IN PLAIN LANGUAGE:**

**_______________________________________________________________________________**

**F76BREGION**

**PERSONAL << TEXT TAB3varv >>: << TEXT >> friends name**

**Do you know of any region in the world << TEXT >> His Her mother was born?**

**READ OUT OPTIONS WHEN NEEDED!**

**No. 1**

**2 Yes, in the Nordic countries / Scandinavia**

**3 Yes, in Western Europe (except the Nordic countries)**

**4 Yes, in Eastern Europe (excluding Turkey)**

**5 Yes, in the Middle East (including Turkey)**

**6 Yes, in Asia (excluding the Middle East)**

**7 Yes, in Africa**

**8 Yes, in North America**

**9 Yes, in South America**

**10 Yes, but none of the above regions**

**_______________________________________________________________________________**

**F76C**

**PERSONAL << TEXT TAB3varv >>: << TEXT >> friends name**

**In which country is << TEXT >> His Her father born?**

**__________________________________________________________________**

**TO IVE: Enter DP's spontaneous reply. Do not answer to get hit in the list,**

**but rather write the country in clear text in the next issue.**

**__________________________________________________________________**

**1 = COUNTRY ARE LISTED**

**COUNTRY:**

**_______________________________________________________________________________**

**F76CTXT**

**PERSONAL << TEXT TAB3varv >>: << TEXT >> friends name**

**(In which country is << TEXT >> His Her father born?)**

**ENTERING THE COUNTRY IN PLAIN LANGUAGE:**

**_______________________________________________________________________________**

**F76CREGION**

**PERSONAL << TEXT TAB3varv >>: << TEXT >> friends name**

**Do you know of any region in the world << TEXT >> His Her father born?**

**READ OUT OPTIONS WHEN NEEDED!**

**No. 1**

**2 Yes, in the Nordic countries / Scandinavia**

**3 Yes, in Western Europe (except the Nordic countries)**

**4 Yes, in Eastern Europe (excluding Turkey)**

**5 Yes, in the Middle East (including Turkey)**

**6 Yes, in Asia (excluding the Middle East)**

**7 Yes, in Africa**

**8 Yes, in North America**

**9 Yes, in South America**

**10 Yes, but none of the above regions**

**_______________________________________________________________________________**

**F78D**

**PERSONAL << TEXT TAB3varv >>: << TEXT >> friends name**

**Went << TEXT >> friends name and you are in the same elementary school or high school?**

**_______________________________________________________________**

**TO IVE: Respond YES even if the UP and friends name << TEXT >> gone to the same school**

**only a short time.**

**_______________________________________________________________**

**1 Yes**

**No. 2**

**_______________________________________________________________________________**

**F78DD**

**PERSONAL << TEXT TAB3varv >>: << TEXT >> friends name**

**Did you go in the same class?**

**_______________________________________________________________**

**TO IVE: Respond YES even if the UP and << TEXT >> friends name gone in the same class**

**only a short time.**

**_______________________________________________________________**

**1 Yes**

**No. 2**

**_______________________________________________________________________________**

**F78F**

**PERSONAL << TEXT TAB3varv >>: << TEXT >> friends name**

**Are you, or have you been, association or club mates?**

**1 Yes, we are the association / club mates**

**2 Yes, we have been the association / club mates**

**No. 3**

**_______________________________________________________________________________**

**F78HS1**

**Visible on the screen!**

**Here are stored responses from F78H:**

**1 Yes, we are fellow students at the university / college**

**_______________________________________________________________________________**

**F78HS2**

**Visible on the screen!**

**Here are stored responses from F78H:**

**2 Yes, we have been fellow students at the university / college**

**_______________________________________________________________________________**

**F78H**

**PERSONAL << TEXT TAB3varv >>: << TEXT >> friends name**

**Are you, or have you been, fellow students at the university / college?**

**SEVERAL ANSWERS CAN BE GIVEN. Press space between the answers.**

**1 Yes, we are fellow students at the university / college**

**2 Yes, we have been fellow students at the university / college**

**No. 3**

**_______________________________________________________________________________**

**F78HHS1**

**Visible on the screen!**

**Here are stored responses from F78H:**

**1 Yes, we are colleagues**

**_______________________________________________________________________________**

**F78HHS2**

**Visible on the screen!**

**Here are stored responses from F78H:**

**2 Yes, we have been colleagues**

**_______________________________________________________________________________**

**F78HH**

**PERSONAL << TEXT TAB3varv >>: << TEXT >> friends name**

**Are you, or have you been, colleagues?**

**SEVERAL ANSWERS CAN BE GIVEN. Press space between the answers.**

**1 Yes, we are colleagues**

**2 Yes, we have been colleagues**

**No. 3**

**_______________________________________________________________________________**

**F79A**

**PERSONAL << TEXT TAB3varv >>: << TEXT >> friends name**

**How often do you meet friends name << TEXT >>?**

**::**

**_______________________________________________________________________________**

**TO IVE: "Meet" means that meet physically, that is to be seen in the same place at the same time**

**(Unlike the phone call or virtual contact phone / IM / SMS or e-mail).**

**With 'usually' means 'normally', i.e. a normal week / month.**

**_______________________________________________________________________________**

**1 Daily**

**2 Several times a week**

**3 About once a week**

**4 Once a month**

**5 Several times a year**

**6 Rarely or never**

**_______________________________________________________________________________**

**F79B**

**PERSONAL << TEXT TAB3varv >>: << TEXT >> friends name**

**How often do you have to be in contact with << TEXT Him Her >> via telephone, Internet, e-mail or text message?**

**::**

**_____________________________________________________________________**

**TO IVE: "Being in contact" means: phone calls, or so-called virtual contact**

**through chat / SMS or e-mail.**

**With 'usually' means 'normally', i.e. a normal week / month.**

**_____________________________________________________________________**

**1 Daily**

**2 Several times a week**

**3 About once a week**

**4 Once a month**

**5 Several times a year**

**6 Rarely or never**

**_______________________________________________________________________________**

**F79DS1**

**Visible on the screen!**

**Here are stored responses from F79D:**

**1 policy and societal**

**_______________________________________________________________________________**

**F79DS2**

**Visible on the screen!**

**Here are stored responses from F79D:**

**2 Culture**

**_______________________________________________________________________________**

**F79DS3**

**Visible on the screen!**

**Here are stored responses from F79D:**

**3 Literature**

**_______________________________________________________________________________**

**F79DS4**

**Visible on the screen!**

**Here are stored responses from F79D:**

**4 Sports**

**_______________________________________________________________________________**

**F79DS5**

**Visible on the screen!**

**Here are stored responses from F79D:**

**5 Religion**

**_______________________________________________________________________________**

**F79DS6**

**Visible on the screen!**

**Here are stored responses from F79D:**

**6 Economy**

**_______________________________________________________________________________**

**F79DS7**

**Visible on the screen!**

**Here are stored responses from F79D:**

**7 Six**

**_______________________________________________________________________________**

**F79D**

**PERSONAL << TEXT TAB3varv >>: << TEXT >> friends name**

**Do you discuss the following with << TEXT >> Her Him?**

**SEVERAL ANSWERS CAN BE GIVEN. Press space between the answers.**

**READ OUT OPTIONS (one by one)!**

**1 policy and societal**

**2 Culture**

**3 Literature**

**4 Sports**

**5 Religion**

**6 Economy**

**7 Six**

**NO 8, none of the above**

**_______________________________________________________________________________**

**F79E**

**PERSONAL << TEXT TAB3varv >>: << TEXT >> friends name**

**What language do you mostly speak with << TEXT >> friends name?**

**Swedish 1**

**2 Persian**

**3 Kurdish**

**4 Arabic**

**5 English**

**6 Albania**

**7 Serbo-Croatian (incl. Serb and Croatian)**

**8 Bosnian**

**9 Other language ...**

**_______________________________________________________________________________**

**F79ETXT**

**PERSONAL << TEXT TAB3varv >>: << TEXT >> friends name**

**(What language do you mostly speak with << TEXT >> friends name?)**

**SET LANGUAGE:**

**_______________________________________________________________________________**

**F710**

**PERSONAL << TEXT TAB3varv >>: << TEXT >> friends name**

**How many years have you known << TEXT >> Her Him?**

**_____________________________________________________________________**

**TO IVE: If the DP does not know, ask the UP appreciate.**

**If less than 1 year, enter 0th**

**If UP responds with an interval, calculate an average and round down.**

**For example, "10-15 years," enter the 12th**

**_____________________________________________________________________**

**NUMBER OF YEARS:**

**_______________________________________________________________________________**

**F711**

**PERSONAL << TEXT TAB3varv >>: << TEXT >> friends name**

**Where you'll meet most of the time?**

**READ OUT OPTIONS WHEN NEEDED!**

**1 At the university / college / school**

**2 In a compound**

**3 on work**

**4 family gatherings**

**5 homes of any of you**

**6 Home at other buddies**

**7 Out on the town**

**8 At any Internet community (eg Facebook)**

**9 on vacation trip / sommarstället**

**10 ... ELSE**

**_______________________________________________________________________________**

**F711TXT**

**PERSONAL << TEXT TAB3varv >>: << TEXT >> friends name**

**(Where you'll meet most of the time?)**

**ENTERING ANOTHER PLACE:**

**_______________________________________________________________________________**

**F712**

**PERSONAL << TEXT TAB3varv >>: << TEXT >> friends name**

**How well do you feel that your relationship is?**

**Responding to a scale of 1 to 5, with 1 being not at all and 5 is very good.**

**1 2 3 4:05 a.m.**

**| --------------- | --------------- | --------------- | - -------------- |**

**not good at all very good**

**_______________________________________________________________________________**

**F713A**

**PERSONAL << TEXT TAB3varv >>: << TEXT >> friends name**

**How much do you trust << TEXT >> friends name?**

**Responding to a scale of 1 to 5, with 1 being not at all and 5 is very much.**

**1 2 3 4:05 a.m.**

**| --------------- | --------------- | --------------- | - -------------- |**

**not very much**

**_______________________________________________________________________________**

**F713B**

**PERSONAL << TEXT TAB3varv >>: << TEXT >> friends name**

**Is this a person that you could discuss a major personal problem with?**

**____________________________________________________________________**

**TO IVE: Personal problems can range from love, illness, studies**

**criminality and abuse.**

**____________________________________________________________________**

**1 Yes**

**No. 2**

**_______________________________________________________________________________**

**F713CS1**

**Visible on the screen!**

**Here are stored responses from F713C;**

**1 nods towards each other**

**_______________________________________________________________________________**

**F713CS2**

**Visible on the screen!**

**Here are stored responses from F713C;**

**2 Say "Hello!" (Or other greeting)**

**_______________________________________________________________________________**

**F713CS3**

**Visible on the screen!**

**Here are stored responses from F713C;**

**Shaking hands 3**

**_______________________________________________________________________________**

**F713CS4**

**Visible on the screen!**

**Here are stored responses from F713C;**

**4 cans each other in the back**

**_______________________________________________________________________________**

**F713CS5**

**Visible on the screen!**

**Here are stored responses from F713C;**

**5 hugging each other**

**_______________________________________________________________________________**

**F713CS6**

**Visible on the screen!**

**Here are stored responses from F713C;**

**6 kiss on the cheek**

**_______________________________________________________________________________**

**F713CS7**

**Visible on the screen!**

**Here are stored responses from F713C;**

**7 Kiss on the mouth**

**_______________________________________________________________________________**

**F713CS8**

**Visible on the screen!**

**Here are stored responses from F713C;**

**8 Otherwise**

**_______________________________________________________________________________**

**F713C**

**PERSONAL << TEXT TAB3varv >>: << TEXT >> friends name**

**How do you greet friends name << TEXT >> if you bump into on the town or on the street?**

**SEVERAL ANSWERS CAN BE GIVEN. Press space between the answers.**

**<< TEXT 'READ OUT THE OPTIONS! 'If TAB3varv = 1 >> << TEXT' READ OUT OPTIONS WHEN NEEDED! 'Of TAB3varv> 1 >>**

**1 nods towards each other**

**2 Say "Hello!" (Or other greeting)**

**Shaking hands 3**

**4 cans each other in the back**

**5 hugging each other**

**6 kiss on the cheek**

**7 Kiss on the mouth**

**8 Otherwise**

**_______________________________________________________________________________**

**F714**

**PERSONAL << TEXT TAB3varv >>: << TEXT >> friends name**

**What is << TEXT >> His Her highest started training?**

**1 Elementary**

**2 Occupation**

**3 High Schools**

**4 School / University**

**_______________________________________________________________________________**

**F715**

**PERSONAL << TEXT TAB3varv >>: << TEXT >> friends name**

**Work or study << TEXT >> friends name, or << TEXT >> HanHon unemployed?**

**1 Yes, work**

**2 Yes, studying**

**3 Yes, both working and studying**

**4 Yes, unemployed**

**No. 5**

**_______________________________________________________________________________**

**F716**

**PERSONAL << TEXT TAB3varv >>: << TEXT >> friends name**

**What is your occupation << TEXT >> HanHon?**

**1 = PROFESSION IS NOT LISTED**

**PROFESSION:**

**_______________________________________________________________________________**

**F716TXT**

**PERSONAL << TEXT TAB3varv >>: << TEXT >> friends name**

**(What is your occupation << TEXT >> HanHon?)**

**___________________________________________________________________________**

**TO IVE: Try to find out the following**

**- Main duties**

**- Industry**

**- Operation (as home care for the elderly, the construction of wall and plaster)**

**- The qualification level (eg management work, military work, workers and employees)**

**___________________________________________________________________________**

**ENTER PROFESSION IN PLAIN LANGUAGE:**

**_______________________________________________________________________________**

**F718A**

**PERSONAL << TEXT TAB3varv >>: << TEXT >> friends name**

**Smoke << TEXT >> friends name?**

**1 Yes**

**No. 2**

**_______________________________________________________________________________**

**F718AA**

**PERSONAL << TEXT TAB3varv >>: << TEXT >> friends name**

**How much?**

**Occasionally << TEXT >> HanHon or smokes << TEXT >> HanHon less than a pack a day**

**or more than a pack a day?**

**1 Occasionally**

**2 smoke less than a pack a day**

**3 Smoking a pack a day or more**

**_______________________________________________________________________________**

**F718B**

**PERSONAL << TEXT TAB3varv >>: << TEXT >> friends name**

**Drinking << TEXT >> Alcohol friends name?**

**1 Yes**

**No. 2**

**_______________________________________________________________________________**

**F718BB**

**PERSONAL << TEXT TAB3varv >>: << TEXT >> friends name**

**About how often do << TEXT >> HanHon so much alcohol that << TEXT >> HanHon gets drunk?**

**READ OUT OPTIONS WHEN NEEDED!**

**1 Three times a week, or more often**

**2 One to two times per week**

**3 Two to three times per month**

**4 Once per month**

**5 More rarely**

**6 Never**

**_______________________________________________________________________________**

**F719A**

**PERSONAL << TEXT TAB3varv >>: << TEXT >> friends name**

**Exercise or sports << TEXT >> friends name?**

**There should be at least half an hour of continuous exercise / sports at every opportunity.**

**::**

**1 Yes**

**No. 2**

**_______________________________________________________________________________**

**F719B**

**PERSONAL << TEXT TAB3varv >>: << TEXT >> friends name**

**Would you say << TEXT >> HanHon training more than you, about the same as you or less than you?**

**::**

**1 More**

**2 As much**

**3 Small**

**_______________________________________________________________________________**

**F719C**

**PERSONAL << TEXT TAB3varv >>: << TEXT >> friends name**

**Do << TEXT >> HanHon eat nutritious and healthy food?**

**1 Yes**

**No. 2**

**_______________________________________________________________________________**

**F719D**

**PERSONAL << TEXT TAB3varv >>: << TEXT >> friends name**

**Is << TEXT >> friends name a person who likes to take risks or try to avoid risks?**

**Responding to a scale of 1 to 10, with 1 being very careful and 10 very risk-averse.**

**1 2 3 4 5 6 7 8 9 10**

**| ---------- | ---------- | ---------- | ---------- | ----- ----- | ---------- | ---------- | ---------- | ---------- |**

**very much**

**careful risk-averse**

**_______________________________________________________________________________**

**F719E**

**PERSONAL << TEXT TAB3varv >>: << TEXT >> friends name**

**How would you describe << TEXT >> His Her physique?**

**As underweight, normal weight, overweight or obese?**

**1 Underweight**

**2 Normal Important**

**3 Overweight**

**4 Very overweight**

**_______________________________________________________________________________**

**F719F**

**PERSONAL << TEXT TAB3varv >>: << TEXT >> friends name**

**Have << TEXT >> HanHon victim of a crime in the last 12 months?**

**1 Yes**

**No. 2**

**_______________________________________________________________________________**

**F720A**

**PERSONAL << TEXT TAB3varv >>: << TEXT >> friends name**

**Is << TEXT >> friends name very religious, very religious, not very religious or not religious?**

**1 Very religious**

**2 Fairly religious**

**3 Not particularly religious**

**4 Not at all religious**

**_______________________________________________________________________________**

**F720B**

**PERSONAL << TEXT TAB3varv >>: << TEXT >> friends name**

**What religion belongs << TEXT >> HanHon?**

**1 Protestant Christianity**

**2 Catholic Christianity**

**3 Orthodox Christianity**

**4 Frikyrklig (including Mormon, Witness etc.)**

**5 Kristendom, unspecified**

**6 Islam, Shia (Muslim = Islam)**

**7 Islam, Sunni (Muslim = Islam)**

**8 Islam - unspecified (Muslim = Islam)**

**9 Judaism**

**... 10 Other**

**_______________________________________________________________________________**

**F720BTXT**

**PERSONAL << TEXT TAB3varv >>: << TEXT >> friends name**

**(What religion belongs << TEXT >> HanHon?)**

**ENTERING ANOTHER RELIGION:**

**_______________________________________________________________________________**

**TAB3SLUT**

**Visible on the screen!**

**Here ends the tableau: Friends**

**_______________________________________________________________________________**

**F721V12A**

**I would also like to know if these people, as you have mentioned know each other.**

**Do << TEXT Van1Namn >> and << TEXT >> Van2Namn each other?**

**1 Yes**

**No. 2**

**_______________________________________________________________________________**

**F721V12B**

**ISSUE CONCERNS: << TEXT Van1Namn >> and << TEXT Van2Namn >>**

**How well do the other?**

**READ OUT THE OPTIONS!**

**1 Not so well**

**2 Fairly well**

**3 Very well**

**_______________________________________________________________________________**

**F721V12C**

**ISSUE CONCERNS: << TEXT Van1Namn >> and << TEXT Van2Namn >>**

**How well will they agree?**

**Responding to a scale of 1 to 5 where 1 is not at all and 5 is very good.**

**1 2 3 4:05 a.m.**

**| --------------- | --------------- | --------------- | - -------------- |**

**not good at all very good**

**_______________________________________________________________________________**

**F721V13A**

**I would also like to know if these people, as you have mentioned know each other.**

**Do << TEXT Van1Namn >> and << TEXT >> Van3Namn each other?**

**1 Yes**

**No. 2**

**_______________________________________________________________________________**

**F721V13B**

**ISSUE CONCERNS: << TEXT Van1Namn >> and << TEXT Van3Namn >>**

**How well do the other?**

**READ OUT THE OPTIONS!**

**1 Not so well**

**2 Fairly well**

**3 Very well**

**_______________________________________________________________________________**

**F721V13C**

**ISSUE CONCERNS: << TEXT Van1Namn >> and << TEXT Van3Namn >>**

**How well will they agree?**

**Responding to a scale of 1 to 5 where 1 is not at all and 5 is very good.**

**1 2 3 4:05 a.m.**

**| --------------- | --------------- | --------------- | - -------------- |**

**not good at all very good**

**_______________________________________________________________________________**

**F721V23A**

**Do << TEXT Van2Namn >> and << TEXT >> Van3Namn each other?**

**1 Yes**

**No. 2**

**_______________________________________________________________________________**

**F721V23B**

**ISSUE CONCERNS: << TEXT Van2Namn >> and << TEXT Van3Namn >>**

**How well do the other?**

**READ OUT THE OPTIONS!**

**1 Not so well**

**2 Fairly well**

**3 Very well**

**_______________________________________________________________________________**

**F721V23C**

**ISSUE CONCERNS: << TEXT Van2Namn >> and << TEXT Van3Namn >>**

**How well will they agree?**

**Responding to a scale of 1 to 5 where 1 is not at all and 5 is very good.**

**1 2 3 4:05 a.m.**

**| --------------- | --------------- | --------------- | - -------------- |**

**not good at all very good**

**_______________________________________________________________________________**

**F721V14A**

**Do << TEXT Van1Namn >> and << TEXT >> Van4Namn each other?**

**1 Yes**

**No. 2**

**_______________________________________________________________________________**

**F721V14B**

**ISSUE CONCERNS: << TEXT Van1Namn >> and << TEXT Van4Namn >>**

**How well do the other?**

**READ OUT THE OPTIONS!**

**1 Not so well**

**2 Fairly well**

**3 Very well**

**_______________________________________________________________________________**

**F721V14C**

**ISSUE CONCERNS: << TEXT Van1Namn >> and << TEXT Van4Namn >>**

**How well will they agree?**

**Responding to a scale of 1 to 5 where 1 is not at all and 5 is very good.**

**1 2 3 4:05 a.m.**

**| --------------- | --------------- | --------------- | - -------------- |**

**not good at all very good**

**_______________________________________________________________________________**

**F721V24A**

**Do << TEXT Van2Namn >> and << TEXT >> Van4Namn each other?**

**1 Yes**

**No. 2**

**_______________________________________________________________________________**

**F721V24B**

**ISSUE CONCERNS: << TEXT Van2Namn >> and << TEXT Van4Namn >>**

**How well do the other?**

**READ OUT THE OPTIONS!**

**1 Not so well**

**2 Fairly well**

**3 Very well**

**_______________________________________________________________________________**

**F721V24C**

**ISSUE CONCERNS: << TEXT Van2Namn >> and << TEXT Van4Namn >>**

**How well will they agree?**

**Responding to a scale of 1 to 5 where 1 is not at all and 5 is very good.**

**1 2 3 4:05 a.m.**

**| --------------- | --------------- | --------------- | - -------------- |**

**not good at all very good**

**_______________________________________________________________________________**

**F721V34A**

**Do << TEXT Van3Namn >> and << TEXT >> Van4Namn each other?**

**1 Yes**

**No. 2**

**_______________________________________________________________________________**

**F721V34B**

**ISSUE CONCERNS: << TEXT Van3Namn >> and << TEXT Van4Namn >>**

**How well do the other?**

**READ OUT THE OPTIONS!**

**1 Not so well**

**2 Fairly well**

**3 Very well**

**_______________________________________________________________________________**

**F721V34C**

**ISSUE CONCERNS: << TEXT Van3Namn >> and << TEXT Van4Namn >>**

**How well will they agree?**

**Responding to a scale of 1 to 5 where 1 is not at all and 5 is very good.**

**1 2 3 4:05 a.m.**

**| --------------- | --------------- | --------------- | - -------------- |**

**not good at all very good**

**_______________________________________________________________________________**

**F721V15A**

**Do << TEXT Van1Namn >> and << TEXT >> Van5Namn each other?**

**1 Yes**

**No. 2**

**_______________________________________________________________________________**

**F721V15B**

**ISSUE CONCERNS: << TEXT Van1Namn >> and << TEXT Van5Namn >>**

**How well do the other?**

**READ OUT THE OPTIONS!**

**1 Not so well**

**2 Fairly well**

**3 Very well**

**_______________________________________________________________________________**

**F721V15C**

**ISSUE CONCERNS: << TEXT Van1Namn >> and << TEXT Van5Namn >>**

**How well will they agree?**

**Responding to a scale of 1 to 5 where 1 is not at all and 5 is very good.**

**1 2 3 4:05 a.m.**

**| --------------- | --------------- | --------------- | - -------------- |**

**not good at all very good**

**_______________________________________________________________________________**

**F721V25A**

**Do << TEXT Van2Namn >> and << TEXT >> Van5Namn each other?**

**1 Yes**

**No. 2**

**_______________________________________________________________________________**

**F721V25B**

**ISSUE CONCERNS: << TEXT Van2Namn >> and << TEXT Van5Namn >>**

**How well do the other?**

**READ OUT THE OPTIONS!**

**1 Not so well**

**2 Fairly well**

**3 Very well**

**_______________________________________________________________________________**

**F721V25C**

**ISSUE CONCERNS: << TEXT Van2Namn >> and << TEXT Van5Namn >>**

**How well will they agree?**

**Responding to a scale of 1 to 5 where 1 is not at all and 5 is very good.**

**1 2 3 4:05 a.m.**

**| --------------- | --------------- | --------------- | - -------------- |**

**not good at all very good**

**_______________________________________________________________________________**

**F721V35A**

**Do << TEXT Van3Namn >> and << TEXT >> Van5Namn each other?**

**1 Yes**

**No. 2**

**_______________________________________________________________________________**

**F721V35B**

**ISSUE CONCERNS: << TEXT Van3Namn >> and << TEXT Van5Namn >>**

**How well do the other?**

**READ OUT THE OPTIONS!**

**1 Not so well**

**2 Fairly well**

**3 Very well**

**_______________________________________________________________________________**

**F721V35C**

**ISSUE CONCERNS: << TEXT Van3Namn >> and << TEXT Van5Namn >>**

**How well will they agree?**

**Responding to a scale of 1 to 5 where 1 is not at all and 5 is very good.**

**1 2 3 4:05 a.m.**

**| --------------- | --------------- | --------------- | - -------------- |**

**not good at all very good**

**_______________________________________________________________________________**

**F721V45A**

**Do << TEXT Van4Namn >> and << TEXT >> Van5Namn each other?**

**1 Yes**

**No. 2**

**_______________________________________________________________________________**

**F721V45B**

**ISSUE CONCERNS: << TEXT Van4Namn >> and << TEXT Van5Namn >>**

**How well do the other?**

**READ OUT THE OPTIONS!**

**1 Not so well**

**2 Fairly well**

**3 Very well**

**_______________________________________________________________________________**

**F721V45C**

**ISSUE CONCERNS: << TEXT Van4Namn >> and << TEXT Van5Namn >>**

**How well will they agree?**

**Responding to a scale of 1 to 5, with 1 being not at all and 5 is very good.**

**1 2 3 4:05 a.m.**

**| --------------- | --------------- | --------------- | - -------------- |**

**not good at all very good**

**_______________________________________________________________________________**

**F722INTRO**

**I will now ask you a few questions about the people you mentioned**

**When we talked three years ago.**

**When you mentioned these people ...**

**READ OUT NAMES!**

**NAME: << >> TEXT OldVan1Namn**

**<< >> TEXT OldVan2Namn**

**<< >> TEXT OldVan3Namn**

**<< >> TEXT OldVan4Namn**

**<< >> TEXT OldVan5Namn**

**1 PRESSURE AND ENTER TO CONTINUE.**

**_______________________________________________________________________________**

**TAB4START**

**Visible on the screen!**

**Here begins the tableau: Old friends**

**_______________________________________________________________________________**

**F722A**

**<< TEXT 'We begin with' if TAB4varv = 1 & Number Vanner> 0 >> << TEXT 'Then we continue with' if TAB4varv> 1 & Number Vanner> 0 >> << TEXT OldVanNamn about the number of friends> 0 >>**

**Is << TEXT >> OldVanNamn identical to any of the << TEXT >> number of friends people you've mentioned earlier in the interview?**

**___________________________________________________________________**

**TO IVE: If the DP has set an alias or just an initial, and not remember**

**which person it refers to, enter F8 = DO NOT KNOW.**

**___________________________________________________________________**

**0 NO**

**<< TEXT '1 Yes, identical' to the number of friends> = 1 >> << >> TEXT Van1Namn**

**<< TEXT '2 Yes, identical' to the number of friends> = 2 >> << >> TEXT Van2Namn**

**<< TEXT '3 Yes, identical' to the number of friends> = 3 >> << >> TEXT Van3Namn**

**<< TEXT '4 Yes, identical' to the number of friends> = 4 >> << >> TEXT Van4Namn**

**<< TEXT '5 Yes, identical' to the number of friends> = 5 >> << >> TEXT Van5Namn**

**_______________________________________________________________________________**

**F722B**

**<< TEXT 'PERSON' to the number of friends> 0 >> << Number of TEXT TAB4varv Friends> 0 >> << TEXT '(from route 1),' Number of Friends> 0 >> << Number of TEXT OldVanNamn Friends> 0 >> < <TEXT 'we begin with' if TAB4varv = 1 & number of friends = 0 >> << TEXT 'Then we continue with' if TAB4varv> 1 & number of friends = 0 >> << TEXT OldVanNamn about the number of friends = 0 >>**

**Do you still have contact with him / her?**

**<< TEXT '_______________________________________________________________________________ 'To the number of friends = 0 >> << TEXT' 1 Yes, often 'To the number of friends> 0 >>**

**<< TEXT 'TO IVE: If the DP has set an alias or just an initial, and not remember 'To the number of friends = 0 >> << TEXT' 2 Yes, sometimes 'To the number of friends> 0 >>**

**which person it refers to, enter F8 (DK) on this and the next two questions. 'To the number of friends = 0 >>**

**<< TEXT '_______________________________________________________________________________ 'To the number of friends = 0 >> << TEXT' 3 No 'To the number of friends> 0 >>**

**<< TEXT '1 Yes, often 'To the number of friends = 0 >>**

**<< TEXT '2 Yes, sometimes 'To the number of friends = 0 >>**

**<< TEXT '3 No 'To the number of friends = 0 >>**

**_______________________________________________________________________________**

**F722C**

**PERSONAL << TEXT >> TAB4varv (from Route 1): << TEXT OldVanNamn >>**

**How well do you feel that your relationship is?**

**Responding to a scale of 1 to 5, with 1 being not at all and 5 is very good.**

**1 2 3 4:05 a.m.**

**| --------------- | --------------- | --------------- | - -------------- |**

**not good at all very good**

**_______________________________________________________________________________**

**F722D**

**PERSONAL << TEXT >> TAB4varv (from Route 1): << TEXT OldVanNamn >>**

**Do you know what he / she is doing today?**

**Working or studying, he / she, or he / she is unemployed?**

**1 Yes, work**

**2 Yes, studying**

**3 Yes, both working and studying**

**4 Yes, unemployed**

**5 No, none of the above**

**_______________________________________________________________________________**

**TAB4SLUT**

**Visible on the screen!**

**Here ends the tableau: Old friends**

**_______________________________________________________________________________**

**F723**

**How many friends do you have (in all)?**

**NUMBER OF FRIENDS:**

**_______________________________________________________________________________**

**F724A**

**How important would you say that social media is for your contact with friends and acquaintances?**

**~~~~~~~~~~~**

**Responding to a scale of 1 to 5, with 1 being not at all important and 5 is very important.**

**1 2 3 4:05 a.m.**

**| --------------- | --------------- | --------------- | - -------------- |**

**not very**

**important important**

**_______________________________________________________________________________**

**F724B**

**How important would you say that the phone is on your contact with friends and acquaintances?**

**~~~~~~~**

**Responding to a scale of 1 to 5, with 1 being not at all important and 5 is very important.**

**1 2 3 4:05 a.m.**

**| --------------- | --------------- | --------------- | - -------------- |**

**not very**

**important important**

**_______________________________________________________________________________**

**F724C**

**How important would you say that face-to-face meetings are for your contact with friends and acquaintances?**

**~~~~~~~~~~~~~~~**

**Responding to a scale of 1 to 5, with 1 being not at all important and 5 is very important.**

**_______________________________________________________________________________**

**TO IVE: "Face-to-face" means to meet physically, that is to be seen in the same place at the same time**

**(Unlike e.g., video call and Skype).**

**_______________________________________________________________________________**

**1 2 3 4:05 a.m.**

**| --------------- | --------------- | --------------- | - -------------- |**

**not very**

**important important**

**_______________________________________________________________________________**

**NY1A**

**Now I will read you a list of professions and ask you to say about any friend,**

**acquaintance, family member, girlfriend / boyfriend or relative has the profession.**

**PROFESSIONAL: Doctors**

**Have a friend, acquaintance, family member, girlfriend / boyfriend or relative of the profession?**

**1 Yes**

**No. 2**

**_______________________________________________________________________________**

**NY1B**

**(PROFESSIONAL: Physician)**

**Lives the person in Sweden or in another country (or both)?**

**1 In Sweden**

**2 In another country**

**3 Both in Sweden and in other countries**

**_______________________________________________________________________________**

**NY2A**

**PROFESSIONAL: Cook**

**Have a friend, acquaintance, family member, girlfriend / boyfriend or relative of the profession?**

**1 Yes**

**No. 2**

**_______________________________________________________________________________**

**NY2B**

**(PROFESSIONAL: Cook)**

**Lives the person in Sweden or in another country (or both)?**

**1 In Sweden**

**2 In another country**

**3 Both in Sweden and in other countries**

**_______________________________________________________________________________**

**NY3A**

**PROFESSIONAL: Construction Worker**

**Have a friend, acquaintance, family member, girlfriend / boyfriend or relative of the profession?**

**1 Yes**

**No. 2**

**_______________________________________________________________________________**

**NY3B**

**(PROFESSIONAL: Construction Worker)**

**Lives the person in Sweden or in another country (or both)?**

**1 In Sweden**

**2 In another country**

**3 Both in Sweden and in other countries**

**_______________________________________________________________________________**

**NY4A**

**OCCUPATION: Assistant**

**Have a friend, acquaintance, family member, girlfriend / boyfriend or relative of the profession?**

**1 Yes**

**No. 2**

**_______________________________________________________________________________**

**NY4B**

**(PROFESSIONAL: Assistant)**

**Lives the person in Sweden or in another country (or both)?**

**1 In Sweden**

**2 In another country**

**3 Both in Sweden and in other countries**

**_______________________________________________________________________________**

**NY5A**

**Occupation: Engineering**

**Have a friend, acquaintance, family member, girlfriend / boyfriend or relative of the profession?**

**1 Yes**

**No. 2**

**_______________________________________________________________________________**

**NY5B**

**(Occupation Engineering)**

**Lives the person in Sweden or in another country (or both)?**

**1 In Sweden**

**2 In another country**

**3 Both in Sweden and in other countries**

**_______________________________________________________________________________**

**NY6A**

**PROFESSIONAL: Barber**

**Have a friend, acquaintance, family member, girlfriend / boyfriend or relative of the profession?**

**1 Yes**

**No. 2**

**_______________________________________________________________________________**

**NY6B**

**(PROFESSIONAL Barber)**

**Lives the person in Sweden or in another country (or both)?**

**1 In Sweden**

**2 In another country**

**3 Both in Sweden and in other countries**

**_______________________________________________________________________________**

**NY7A**

**PROFESSIONAL: Postman**

**Have a friend, acquaintance, family member, girlfriend / boyfriend or relative of the profession?**

**1 Yes**

**No. 2**

**_______________________________________________________________________________**

**NY7B**

**(PROFESSIONAL: Mailman)**

**Lives the person in Sweden or in another country (or both)?**

**1 In Sweden**

**2 In another country**

**3 Both in Sweden and in other countries**

**_______________________________________________________________________________**

**NY8A**

**OCCUPATION: Lawyer**

**Have a friend, acquaintance, family member, girlfriend / boyfriend or relative of the profession?**

**1 Yes**

**No. 2**

**_______________________________________________________________________________**

**NY8B**

**(Profession: Lawyer)**

**Lives the person in Sweden or in another country (or both)?**

**1 In Sweden**

**2 In another country**

**3 Both in Sweden and in other countries**

**_______________________________________________________________________________**

**NY9A**

**OCCUPATION: Personal assistant**

**Have a friend, acquaintance, family member, girlfriend / boyfriend or relative of the profession?**

**1 Yes**

**No. 2**

**_______________________________________________________________________________**

**NY9B**

**(PROFESSIONAL: Assistant)**

**Lives the person in Sweden or in another country (or both)?**

**1 In Sweden**

**2 In another country**

**3 Both in Sweden and in other countries**

**_______________________________________________________________________________**

**NY10A**

**PROFESSION: Industrial**

**Have a friend, acquaintance, family member, girlfriend / boyfriend or relative of the profession?**

**1 Yes**

**No. 2**

**_______________________________________________________________________________**

**NY10B**

**(PROFESSIONAL: Industrial)**

**Lives the person in Sweden or in another country (or both)?**

**1 In Sweden**

**2 In another country**

**3 Both in Sweden and in other countries**

**_______________________________________________________________________________**

**NY11A**

**PROFESSIONAL: Telemarketer**

**Have a friend, acquaintance, family member, girlfriend / boyfriend or relative of the profession?**

**1 Yes**

**No. 2**

**_______________________________________________________________________________**

**NY11B**

**(PROFESSIONAL: Dealer Telephone)**

**Lives the person in Sweden or in another country (or both)?**

**1 In Sweden**

**2 In another country**

**3 Both in Sweden and in other countries**

**_______________________________________________________________________________**

**NY12A**

**OCCUPATION: Teacher at primary school or secondary school**

**Have a friend, acquaintance, family member, girlfriend / boyfriend or relative of the profession?**

**1 Yes**

**No. 2**

**_______________________________________________________________________________**

**NY12B**

**(PROFESSIONAL: Teacher on primary or secondary)**

**Lives the person in Sweden or in another country (or both)?**

**1 In Sweden**

**2 In another country**

**3 Both in Sweden and in other countries**

**_______________________________________________________________________________**

**NY13A**

**OCCUPATION: Nurse**

**Have a friend, acquaintance, family member, girlfriend / boyfriend or relative of the profession?**

**1 Yes**

**No. 2**

**_______________________________________________________________________________**

**NY13B**

**(PROFESSIONAL: Nurse)**

**Lives the person in Sweden or in another country (or both)?**

**1 In Sweden**

**2 In another country**

**3 Both in Sweden and in other countries**

**_______________________________________________________________________________**

**NY14A**

**Profession: Truck driver**

**Have a friend, acquaintance, family member, girlfriend / boyfriend or relative of the profession?**

**1 Yes**

**No. 2**

**_______________________________________________________________________________**

**NY14B**

**(PROFESSIONAL: Truck driver)**

**Lives the person in Sweden or in another country (or both)?**

**1 In Sweden**

**2 In another country**

**3 Both in Sweden and in other countries**

**_______________________________________________________________________________**

**NY15A**

**OCCUPATION: Real estate**

**Have a friend, acquaintance, family member, girlfriend / boyfriend or relative of the profession?**

**1 Yes**

**No. 2**

**_______________________________________________________________________________**

**NY15B**

**(PROFESSIONAL: Estate)**

**Lives the person in Sweden or in another country (or both)?**

**1 In Sweden**

**2 In another country**

**3 Both in Sweden and in other countries**

**_______________________________________________________________________________**

**NY16A**

**OCCUPATION: Musician full time**

**Have a friend, acquaintance, family member, girlfriend / boyfriend or relative of the profession?**

**1 Yes**

**No. 2**

**_______________________________________________________________________________**

**NY16B**

**(PROFESSIONAL: Musicians full time)**

**Lives the person in Sweden or in another country (or both)?**

**1 In Sweden**

**2 In another country**

**3 Both in Sweden and in other countries**

**_______________________________________________________________________________**

**NY17A**

**OCCUPATION: Police**

**Have a friend, acquaintance, family member, girlfriend / boyfriend or relative of the profession?**

**1 Yes**

**No. 2**

**_______________________________________________________________________________**

**NY17B**

**(PROFESSIONAL: Police)**

**Lives the person in Sweden or in another country (or both)?**

**1 In Sweden**

**2 In another country**

**3 Both in Sweden and in other countries**

**_______________________________________________________________________________**

**NY18A**

**OCCUPATION: Cleaners**

**Have a friend, acquaintance, family member, girlfriend / boyfriend or relative of the profession?**

**1 Yes**

**No. 2**

**_______________________________________________________________________________**

**NY18B**

**(PROFESSIONAL: The cleaners)**

**Lives the person in Sweden or in another country (or both)?**

**1 In Sweden**

**2 In another country**

**3 Both in Sweden and in other countries**

**_______________________________________________________________________________**

**NY19A**

**PROFESSIONAL: Dentists**

**Have a friend, acquaintance, family member, girlfriend / boyfriend or relative of the profession?**

**1 Yes**

**No. 2**

**_______________________________________________________________________________**

**NY19B**

**(PROFESSIONAL: Dentists)**

**Lives the person in Sweden or in another country (or both)?**

**1 In Sweden**

**2 In another country**

**3 Both in Sweden and in other countries**

**_______________________________________________________________________________**

**NY20A**

**PROFESSION: Mechanic, eg mechanic**

**Have a friend, acquaintance, family member, girlfriend / boyfriend or relative of the profession?**

**1 Yes**

**No. 2**

**_______________________________________________________________________________**

**NY20B**

**(PROFESSIONAL: Mechanics, such as auto mechanic)**

**Lives the person in Sweden or in another country (or both)?**

**1 In Sweden**

**2 In another country**

**3 Both in Sweden and in other countries**

**_______________________________________________________________________________**

**NY21A**

**Occupation: Nanny**

**Have a friend, acquaintance, family member, girlfriend / boyfriend or relative of the profession?**

**1 Yes**

**No. 2**

**_______________________________________________________________________________**

**NY21B**

**(PROFESSIONAL: Barnskötare)**

**Lives the person in Sweden or in another country (or both)?**

**1 In Sweden**

**2 In another country**

**3 Both in Sweden and in other countries**

**_______________________________________________________________________________**

**NY22A**

**OCCUPATION: Self-employed with own employees**

**Have a friend, acquaintance, family member, girlfriend / boyfriend or relative of the profession?**

**1 Yes**

**No. 2**

**_______________________________________________________________________________**

**NY22B**

**(OCCUPATION: Self-employed with their own employees)**

**Lives the person in Sweden or in another country (or both)?**

**1 In Sweden**

**2 In another country**

**3 Both in Sweden and in other countries**

**_______________________________________________________________________________**

**NY23A**

**PROFESSIONAL: Cashiers**

**Have a friend, acquaintance, family member, girlfriend / boyfriend or relative of the profession?**

**1 Yes**

**No. 2**

**_______________________________________________________________________________**

**NY23B**

**(PROFESSIONAL: cashiers)**

**Lives the person in Sweden or in another country (or both)?**

**1 In Sweden**

**2 In another country**

**3 Both in Sweden and in other countries**

**_______________________________________________________________________________**

**NY24A**

**OCCUPATION: Guardian**

**Have a friend, acquaintance, family member, girlfriend / boyfriend or relative of the profession?**

**1 Yes**

**No. 2**

**_______________________________________________________________________________**

**NY24B**

**(PROFESSIONAL: Security Guard)**

**Lives the person in Sweden or in another country (or both)?**

**1 In Sweden**

**2 In another country**

**3 Both in Sweden and in other countries**

**_______________________________________________________________________________**

**NY25A**

**PROFESSION: Journalist**

**Have a friend, acquaintance, family member, girlfriend / boyfriend or relative of the profession?**

**1 Yes**

**No. 2**

**_______________________________________________________________________________**

**NY25B**

**(PROFESSIONAL: reporter)**

**Lives the person in Sweden or in another country (or both)?**

**1 In Sweden**

**2 In another country**

**3 Both in Sweden and in other countries**

**_______________________________________________________________________________**

**NY26A**

**OCCUPATION: Actor fulltime**

**Have a friend, acquaintance, family member, girlfriend / boyfriend or relative of the profession?**

**1 Yes**

**No. 2**

**_______________________________________________________________________________**

**NY26B**

**(Profession: Actor full time)**

**Lives the person in Sweden or in another country (or both)?**

**1 In Sweden**

**2 In another country**

**3 Both in Sweden and in other countries**

**_______________________________________________________________________________**

**NY27A**

**Profession: Receptionist**

**Have a friend, acquaintance, family member, girlfriend / boyfriend or relative of the profession?**

**1 Yes**

**No. 2**

**_______________________________________________________________________________**

**NY27B**

**(PROFESSIONAL: receptionist)**

**Lives the person in Sweden or in another country (or both)?**

**1 In Sweden**

**2 In another country**

**3 Both in Sweden and in other countries**

**_______________________________________________________________________________**

**NY28A**

**OCCUPATION: Finance**

**Have a friend, acquaintance, family member, girlfriend / boyfriend or relative of the profession?**

**1 Yes**

**No. 2**

**_______________________________________________________________________________**

**NY28B**

**(PROFESSIONAL: CFO)**

**Lives the person in Sweden or in another country (or both)?**

**1 In Sweden**

**2 In another country**

**3 Both in Sweden and in other countries**

**_______________________________________________________________________________**

**NY29A**

**Student at university**

**Have a friend, acquaintance, family member, girlfriend / boyfriend or relative of the profession?**

**1 Yes**

**No. 2**

**_______________________________________________________________________________**

**NY29B**

**(Student at the University)**

**Lives the person in Sweden or in another country (or both)?**

**1 In Sweden**

**2 In another country**

**3 Both in Sweden and in other countries**

**_______________________________________________________________________________**

**NY30A**

**PROFESSIONAL: Taxi driver**

**Have a friend, acquaintance, family member, girlfriend / boyfriend or relative of the profession?**

**1 Yes**

**No. 2**

**_______________________________________________________________________________**

**NY30B**

**(PROFESSIONAL: taxi driver)**

**Lives the person in Sweden or in another country (or both)?**

**1 In Sweden**

**2 In another country**

**3 Both in Sweden and in other countries**

**_______________________________________________________________________________**

**NY31A**

**OCCUPATION: President**

**Have a friend, acquaintance, family member, girlfriend / boyfriend or relative of the profession?**

**1 Yes**

**No. 2**

**_______________________________________________________________________________**

**NY31B**

**(PROFESSIONAL: Rector)**

**Lives the person in Sweden or in another country (or both)?**

**1 In Sweden**

**2 In another country**

**3 Both in Sweden and in other countries**

**_______________________________________________________________________________**

**NY32A**

**PROFESSIONAL: Computer technician**

**Have a friend, acquaintance, family member, girlfriend / boyfriend or relative of the profession?**

**1 Yes**

**No. 2**

**_______________________________________________________________________________**

**NY32B**

**(PROFESSIONAL: Computer technician)**

**Lives the person in Sweden or in another country (or both)?**

**1 In Sweden**

**2 In another country**

**3 Both in Sweden and in other countries**

**_______________________________________________________________________________**

**NY33A**

**PROFESSIONAL: Recreation leader**

**Have a friend, acquaintance, family member, girlfriend / boyfriend or relative of the profession?**

**1 Yes**

**No. 2**

**_______________________________________________________________________________**

**NY33B**

**(PROFESSIONAL: Recreation leader)**

**Lives the person in Sweden or in another country (or both)?**

**1 In Sweden**

**2 In another country**

**3 Both in Sweden and in other countries**

**_______________________________________________________________________________**

**NY34A**

**Profession: Bank employee**

**Have a friend, acquaintance, family member, girlfriend / boyfriend or relative of the profession?**

**1 Yes**

**No. 2**

**_______________________________________________________________________________**

**NY34B**

**(Profession: Bank employee)**

**Lives the person in Sweden or in another country (or both)?**

**1 In Sweden**

**2 In another country**

**3 Both in Sweden and in other countries**

**_______________________________________________________________________________**

**NY35A**

**PROFESSIONAL: Warehouse worker**

**Have a friend, acquaintance, family member, girlfriend / boyfriend or relative of the profession?**

**1 Yes**

**No. 2**

**_______________________________________________________________________________**

**NY35B**

**(PROFESSIONAL: Warehouseman)**

**Lives the person in Sweden or in another country (or both)?**

**1 In Sweden**

**2 In another country**

**3 Both in Sweden and in other countries**

**_______________________________________________________________________________**

**NY36A**

**PROFESSIONAL: Computer Programmers**

**Have a friend, acquaintance, family member, girlfriend / boyfriend or relative of the profession?**

**1 Yes**

**No. 2**

**_______________________________________________________________________________**

**NY36B**

**(PROFESSIONAL computer programmer)**

**Lives the person in Sweden or in another country (or both)?**

**1 In Sweden**

**2 In another country**

**3 Both in Sweden and in other countries**

**_______________________________________________________________________________**

**NY37A**

**OCCUPATION: Accountant**

**Have a friend, acquaintance, family member, girlfriend / boyfriend or relative of the profession?**

**1 Yes**

**No. 2**

**_______________________________________________________________________________**

**NY37B**

**(PROFESSIONAL: Auditor)**

**Lives the person in Sweden or in another country (or both)?**

**1 In Sweden**

**2 In another country**

**3 Both in Sweden and in other countries**

**_______________________________________________________________________________**

**NY38A**

**PROFESSIONAL: Caretaker**

**Have a friend, acquaintance, family member, girlfriend / boyfriend or relative of the profession?**

**1 Yes**

**No. 2**

**_______________________________________________________________________________**

**NY38B**

**(PROFESSIONAL: Valet)**

**Lives the person in Sweden or in another country (or both)?**

**1 In Sweden**

**2 In another country**

**3 Both in Sweden and in other countries**

**_______________________________________________________________________________**

**NY39A**

**PROFESSIONAL: Scientists**

**Have a friend, acquaintance, family member, girlfriend / boyfriend or relative of the profession?**

**1 Yes**

**No. 2**

**_______________________________________________________________________________**

**NY39B**

**(PROFESSIONAL: Scientists)**

**Lives the person in Sweden or in another country (or both)?**

**1 In Sweden**

**2 In another country**

**3 Both in Sweden and in other countries**

**_______________________________________________________________________________**

**NY40A**

**PROFESSION: Waiter or waitress**

**Have a friend, acquaintance, family member, girlfriend / boyfriend or relative of the profession?**

**1 Yes**

**No. 2**

**_______________________________________________________________________________**

**NY40B**

**PROFESSION: Waiter or waitress**

**Lives the person in Sweden or in another country (or both)?**

**1 In Sweden**

**2 In another country**

**3 Both in Sweden and in other countries**

**_______________________________________________________________________________**

**F91**

**Thanks! Now I would like to go over to ask some questions**

**if your current employment ...**

**What is your current occupation?**

**You study, you work, you are unemployed, or do you do something else?**

**1 only Studying**

**2 operates only**

**3 study and work**

**4 unemployed**

**5 Do something else ...**

**_______________________________________________________________________________**

**F92**

**What would you say is your current occupation?**

**Are you at home and takes care of the household, or are you unemployed or on sick leave,**

**or do nothing, or is there something else you do?**

**1 Managing Household (NOT TO PAY EMPLOYEE)**

**2 sick**

**3 Nothing**

**4 Other, namely ...**

**_______________________________________________________________________________**

**F92TXT**

**(What would you say is your current occupation?)**

**ENTERING ANOTHER JOB:**

**_______________________________________________________________________________**

**F103AS1**

**Visible on the screen!**

**Here are stored responses from F103A:**

**No. 1**

**_______________________________________________________________________________**

**F103AS2**

**Visible on the screen!**

**Here are stored responses from F103A:**

**2 Yes Komvux**

**_______________________________________________________________________________**

**F103AS3**

**Visible on the screen!**

**Here are stored responses from F103A:**

**3 Yes, university / college**

**_______________________________________________________________________________**

**F103AS4**

**Visible on the screen!**

**Here are stored responses from F103A:**

**4 Yes, college**

**_______________________________________________________________________________**

**F103AS5**

**Visible on the screen!**

**Here are stored responses from F103A:**

**5 Yes, other education ...**

**_______________________________________________________________________________**

**F103A**

**<< TEXT 'see' if F91 = (1, 3) >> << TEXT 'Have you read' if F91 = (1, 3) >> in adult education, college / university, college or other education?**

**SEVERAL ANSWERS CAN BE GIVEN. Press space between the answers.**

**<< TEXT '1 No' if F91 = (1, 3) >>**

**2 Yes Komvux**

**3 Yes, university / college**

**4 Yes, college**

**5 Yes, other education ...**

**_______________________________________________________________________________**

**F103ATXT**

**(<< TEXT 'see' if F91 = (1, 3) >> << TEXT 'Have you read' if F91 = (1, 3) >> in adult education, college / university, college or other education? )**

**ENTERING OTHER EDUCATION:**

**_______________________________________________________________________________**

**F103B**

**Have you completed (ie expired) the training?**

**1 Yes**

**No. 2**

**_______________________________________________________________________________**

**F103C**

**At the moment, you have one or more jobs?**

**Expect even with jobs you now might have holiday away or sick leave from.**

**1 Yes, a job**

**2 Yes, more jobs**

**<< TEXT '3 No, no job' to F91 = (2, 3) >>**

**_______________________________________________________________________________**

**F103D**

**For what occupation do you expect your work?**

**1 = PROFESSION ARE LISTED**

**PROFESSION:**

**_______________________________________________________________________________**

**F103DTXT**

**(For what occupation do you expect your work?)**

**___________________________________________________________________________**

**TO IVE: Try to find out the following**

**- Main duties**

**- Industry**

**- Operation (as home care for the elderly, the construction of wall and plaster)**

**- The qualification level (eg management work, military work, workers and employees)**

**___________________________________________________________________________**

**ENTER PROFESSIONAL clearly:**

**_______________________________________________________________________________**

**F103E**

**How many jobs have you at the moment?**

**ENTER NUMBER OF JOBS:**

**_______________________________________________________________________________**

**F103F**

**For what occupation do you expect your main job?**

**~~~~~~~~~~**

**1 = PROFESSION ARE LISTED**

**PROFESSION:**

**_______________________________________________________________________________**

**F103FTXT**

**(For what occupation do you expect your main job?)**

**~~~~~~~~~~**

**___________________________________________________________________________**

**TO IVE: Try to find out the following**

**- Main duties**

**- Industry**

**- Operation (as home care for the elderly, the construction of wall and plaster)**

**- The qualification level (eg management work, military work, workers and employees)**

**___________________________________________________________________________**

**ENTER PROFESSIONAL clearly:**

**_______________________________________________________________________________**

**F103G**

**Is your employer a family member?**

**1 Yes**

**No. 2**

**_______________________________________________________________________________**

**F103H**

**If you work full time or part time?**

**::**

**1 Full**

**Part 2**

**_______________________________________________________________________________**

**F103I**

**Is the job fixed or temporary?**

**1 Solid**

**2 Limited**

**_______________________________________________________________________________**

**F103J**

**Did any of the people you mentioned before that helped you get the job?**

**___________________________________________________________________**

**TO IVE: The following friends provided earlier in the interview:**

**<< >> TEXT Van1Namn**

**<< >> TEXT Van2Namn**

**<< >> TEXT Van3Namn**

**<< >> TEXT Van4Namn**

**<< >> TEXT Van5Namn**

**___________________________________________________________________**

**1 Yes**

**No. 2**

**_______________________________________________________________________________**

**F103JNAMN**

**Visible on the screen!**

**It stores the name of the person named in the case F103JJ.**

**_______________________________________________________________________________**

**F103JJ**

**Which of them?**

**READ OUT OPTIONS WHEN NEEDED!**

**<< TEXT '1' to the number of friends> = 1 >> << >> TEXT Van1Namn**

**<< TEXT '2' if the number of friends> = 2 >> << >> TEXT Van2Namn**

**<< TEXT '3' if the number of friends> = 3 >> << >> TEXT Van3Namn**

**<< TEXT '4' to the number of friends> = 4 >> << >> TEXT Van4Namn**

**<< TEXT '5' to the number of friends> = 5 >> << >> TEXT Van5Namn**

**_______________________________________________________________________________**

**F104**

**Have you during the past three years have had a summer job or extra work?**

**1 Yes**

**No. 2**

**_______________________________________________________________________________**

**F105B**

**Was it one of those names you mentioned before that helped you get the job?**

**___________________________________________________________________**

**TO IVE: The following friends provided earlier in the interview:**

**<< >> TEXT Van1Namn**

**<< >> TEXT Van2Namn**

**<< >> TEXT Van3Namn**

**<< >> TEXT Van4Namn**

**<< >> TEXT Van5Namn**

**___________________________________________________________________**

**1 Yes**

**No. 2**

**_______________________________________________________________________________**

**F105BNAMN**

**Visible on the screen!**

**It stores the name of the person named in the case F105BB.**

**_______________________________________________________________________________**

**F105BB**

**Which of them?**

**READ OUT OPTIONS WHEN NEEDED!**

**<< TEXT '1' to the number of friends> = 1 >> << >> TEXT Van1Namn**

**<< TEXT '2' if the number of friends> = 2 >> << >> TEXT Van2Namn**

**<< TEXT '3' if the number of friends> = 3 >> << >> TEXT Van3Namn**

**<< TEXT '4' to the number of friends> = 4 >> << >> TEXT Van4Namn**

**<< TEXT '5' to the number of friends> = 5 >> << >> TEXT Van5Namn**

**_______________________________________________________________________________**

**F106B**

**If you got the offer of a permanent job on the job,**

**would you be interested in that?**

**-------------------------------------------------- -------------------------------------------**

**TO IVE: The question refers to the summer / student job that UP had**

**during the last three years.**

**-------------------------------------------------- -------------------------------------------**

**1 Yes**

**No. 2**

**_______________________________________________________________________________**

**F107**

**Are you in search of work that is not a summer job or extra work?**

**1 Yes**

**No. 2**

**_______________________________________________________________________________**

**F108S1**

**Visible on the screen!**

**Here are stored response from the F108:**

**1 Through employment service**

**_______________________________________________________________________________**

**F108S2**

**Visible on the screen!**

**Here are stored response from the F108:**

**2 View ads in the newspaper**

**_______________________________________________________________________________**

**F108S3**

**Visible on the screen!**

**Here are stored response from the F108:**

**3 online job sites**

**_______________________________________________________________________________**

**F108S4**

**Visible on the screen!**

**Here are stored response from the F108:**

**4 Ringer / write letters to employers**

**_______________________________________________________________________________**

**F108S5**

**Visible on the screen!**

**Here are stored response from the F108:**

**5 Asking around among friends and relatives**

**_______________________________________________________________________________**

**F108S6**

**Visible on the screen!**

**Here are stored response from the F108:**

**6 Prompts current employer**

**_______________________________________________________________________________**

**F108S7**

**Visible on the screen!**

**Here are stored response from the F108:**

**7 Other ...**

**_______________________________________________________________________________**

**F108**

**If you << TEXT 'looking' for F107 = 1 >> << TEXT 'would search' if F107 = 1 >> work - where << TEXT 'get' if F107 = 1 >> << TEXT 'would you get 'if F107 = 1 >> information about vacancies?**

**From the employment office, by reading the ads in the paper, job sites online,**

**by calling or writing to the employer, ask around among friends and relatives,**

**ask your current employer, or something else?**

**____________________________________________________________**

**TO IVE: Read one answer at a time and note the DP's response.**

**____________________________________________________________**

**SEVERAL ANSWERS CAN BE GIVEN. Press space between the answers.**

**1 Through employment service**

**2 View ads in the newspaper**

**3 online job sites**

**4 Ringer / write letters to employers**

**5 Asking around among friends and relatives**

**6 Prompts current employer**

**7 Other ...**

**_______________________________________________________________________________**

**F108TXT**

**(If you << TEXT 'looking' for F107 = 1 >> << TEXT 'would search' if F107 = 1 >> work - where << TEXT 'get' if F107 = 1 >> << TEXT 'would you get 'if F107 = 1 >> information about vacancies?)**

**ENTERING another search method clearly:**

**_______________________________________________________________________________**

**F108C**

**If you << TEXT 'looking' for F107 = 1 >> << TEXT 'would search' if F107 = 1 >> job now, how good chance do you think you have to have a (new) job within 6 months ?**

**Very good, good, reasonably, bad or very bad?**

**1 Very good**

**2 Good**

**3 Fair**

**4 Bad**

**5 Very poor**

**_______________________________________________________________________________**

**F108D**

**If you look back at the past 12 months, has anyone mentioned or tipped you personally**

**if the job without you asking, for example, through conversations, phone, email?**

**1 Yes**

**No. 2**

**_______________________________________________________________________________**

**F108E**

**How many of these tips do you have?**

**Number of tips:**

**_______________________________________________________________________________**

**F131**

**Now I will ask some questions, including about health.**

**I start with exercise during leisure time.**

**Do you exercise regularly at least once a week in your spare time?**

**Counting only exercise sessions that are 30 minutes or longer.**

**1 Yes**

**No. 2**

**_______________________________________________________________________________**

**F132**

**How many days a week do you exercise continuous for at least 30 minutes?**

**_______________________________________________________________**

**TO IVE: Refers to a normal week. if UP is difficult to say exactly,**

**enter the lower option. E.g. "3-4 days," enter the third**

**_______________________________________________________________**

**NUMBER OF DAYS PER WEEK:**

**_______________________________________________________________________________**

**F143**

**How tall are you?**

**LENGTH (CM):**

**_______________________________________________________________________________**

**F144**

**About how much do you weigh?**

**Weight (kg):**

**_______________________________________________________________________________**

**F145**

**When choosing what to eat, how important it is that the food is wholesome and healthy?**

**1 Very important**

**2 Quite important**

**3 Not important**

**_______________________________________________________________________________**

**F150**

**How would you rate your overall health?**

**Is it very good, good, reasonably, bad or very bad?**

**1 Very good**

**2 Good**

**3 Fair**

**4 Poor**

**5 Very poor**

**_______________________________________________________________________________**

**F160**

**Do you regularly use any prescription medication?**

**1 Yes**

**No. 2**

**_______________________________________________________________________________**

**F171**

**Have you during the last 12 months had any of the following illnesses or conditions ...**

**... headaches or migraines?**

**READ OUT THE OPTIONS!**

**1 Severe inconvenience**

**2 Light inconvenience**

**3 No trouble**

**_______________________________________________________________________________**

**F172**

**(Have you in the last 12 months had ...)**

**... Stomach pain or stomach ache?**

**1 Severe inconvenience**

**2 Light inconvenience**

**3 No trouble**

**_______________________________________________________________________________**

**F173**

**(Have you in the last 12 months had ...)**

**... Pain in the back or neck?**

**1 Severe inconvenience**

**2 Light inconvenience**

**3 No trouble**

**_______________________________________________________________________________**

**F174**

**(Have you in the last 12 months had ...)**

**... General fatigue?**

**_______________________________________________________________________________**

**CLARIFY IF NECESSARY: lethargic and powerless for days and nights, not just tired in the morning.**

**_______________________________________________________________________________**

**1 Severe inconvenience**

**2 Light inconvenience**

**3 No trouble**

**_______________________________________________________________________________**

**F175**

**(Have you in the last 12 months had ...)**

**... Insomnia?**

**____________________________________________________**

**CLARIFY IF NECESSARY: Hard to sleep at night.**

**____________________________________________________**

**1 Severe inconvenience**

**2 Light inconvenience**

**3 No trouble**

**_______________________________________________________________________________**

**F176**

**(Have you in the last 12 months had ...)**

**... Anxiety, worry or anxiety?**

**1 Severe inconvenience**

**2 Light inconvenience**

**3 No trouble**

**_______________________________________________________________________________**

**F177**

**(Have you in the last 12 months had ...)**

**... Depression?**

**____________________________________________________**

**CLARIFY IF NECESSARY: Depression.**

**____________________________________________________**

**1 Severe inconvenience**

**2 Light inconvenience**

**3 No trouble**

**_______________________________________________________________________________**

**F1720**

**How often do you have a cold?**

**1 Very often**

**Quite often 2**

**3 Not very often**

**4 Never**

**_______________________________________________________________________________**

**F181**

**Do you smoke a day?**

**1 Yes**

**No. 2**

**_______________________________________________________________________________**

**F182**

**How many cigarettes do you smoke a day on average?**

**_________________________________________**

**TO IVE: 1 pack = 20 cigarettes**

**_________________________________________**

**NUMBER OF CIGARETTES A DAY:**

**_______________________________________________________________________________**

**F183**

**Do you smoke once in a while?**

**1 Yes**

**No. 2**

**_______________________________________________________________________________**

**F191**

**Have you lost weight in the last 12 months?**

**1 Yes**

**No. 2**

**_______________________________________________________________________________**

**F192**

**On how many occasions have you dieted the past 12 months?**

**__________________________________________________________________________**

**TO IVE: What is meant by one point and the length of the pause between two times**

**defined by UP itself. If the UP claims to have sweet potato throughout the year without interruption,**

**enter one time.**

**__________________________________________________________________________**

**ENTER NUMBER OF TIMES:**

**_______________________________________________________________________________**

**F201**

**Do you drink alcohol?**

**1 Yes**

**No. 2**

**_______________________________________________________________________________**

**F202**

**How often?**

**READ OUT THE OPTIONS!**

**1 Three days a week or more often**

**2 One to two times per week**

**3 Two to three times per month**

**4 Once per month**

**5 More rarely**

**_______________________________________________________________________________**

**F203**

**Approximately how many times have you, during the past 12 months,**

**drunk so much alcohol that you were drunk?**

**1 Three days a week or more often**

**2 One to two times per week**

**3 Two to three times per month**

**4 Once per month**

**5 More rarely**

**6 Never**

**_______________________________________________________________________________**

**F211**

**Have you during the past 12 months smoked cannabis?**

**1 Yes**

**No. 2**

**_______________________________________________________________________________**

**F221**

**Have you during the past three months, in other words the last 90 days,**

**because of personal illness visited the doctor at the health center, private clinics,**

**hospital or school doctor?**

**1 Yes**

**No. 2**

**_______________________________________________________________________________**

**F222**

**How many times?**

**ENTER NUMBER OF TIMES:**

**_______________________________________________________________________________**

**F222A**

**Have you during the past 12 months been subjected to some kind of crime?**

**1 Yes**

**No. 2**

**_______________________________________________________________________________**

**F222BS1**

**Visible on the screen!**

**Here are stored responses from F222B:**

**1 Threat of Violence**

**_______________________________________________________________________________**

**F222BS2**

**Visible on the screen!**

**Here are stored responses from F222B:**

**2 Sexual abuse**

**_______________________________________________________________________________**

**F222BS3**

**Visible on the screen!**

**Here are stored responses from F222B:**

**3, sexual harassment**

**_______________________________________________________________________________**

**F222BS4**

**Visible on the screen!**

**Here are stored responses from F222B:**

**4 Assault**

**_______________________________________________________________________________**

**F222BS5**

**Visible on the screen!**

**Here are stored responses from F222B:**

**Wafers 5**

**_______________________________________________________________________________**

**F222BS6**

**Visible on the screen!**

**Here are stored responses from F222B:**

**6 been stolen something valuable**

**_______________________________________________________________________________**

**F222BS7**

**Visible on the screen!**

**Here are stored responses from F222B:**

**7 Burglary**

**_______________________________________________________________________________**

**F222BS8**

**Visible on the screen!**

**Here are stored responses from F222B:**

**8 gunpoint**

**_______________________________________________________________________________**

**F222BS9**

**Visible on the screen!**

**Here are stored responses from F222B:**

**9 bullying**

**_______________________________________________________________________________**

**F222BS10**

**Visible on the screen!**

**Here are stored responses from F222B:**

**10 Others**

**_______________________________________________________________________________**

**F222B**

**What kind of crime?**

**ADDITIONAL QUESTION: Have you been the victim of something more?**

**SEVERAL ANSWERS CAN BE GIVEN. Press space between the answers.**

**1 Threat of Violence**

**2 Sexual abuse**

**3, sexual harassment**

**4 Assault**

**Wafers 5**

**6 been stolen something valuable**

**7 Burglary**

**8 gunpoint**

**9 bullying**

**10 Others**

**_______________________________________________________________________________**

**F222C**

**Do you worry about being exposed to criminal acts?**

**1 Yes, often**

**2 Yes, sometimes**

**No. 3**

**_______________________________________________________________________________**

**F222D**

**Are you afraid to walk alone in your neighborhood?**

**1 Yes, often**

**2 Yes, sometimes**

**No. 3**

**_______________________________________________________________________________**

**F223A**

**Now I will move on to other issues.**

**If you were to describe who you are, what would be most important to you**

**to say nothing about? That you ...**

**READ OUT THE OPTIONS!**

**1 ... has a particular religion?**

**2 ... born in a certain country?**

**3 ... have a particular gender?**

**4 ... has a certain political view?**

**5 ... has a particular sexual orientation?**

**6 ... belongs to a certain class of society?**

**7 ... has a certain style of music?**

**8 ... living in a particular area / site?**

**9 ... belong to a certain ethnic group?**

**10 ... or you rooting for a particular team?**

**_______________________________________________________________________________**

**F223B**

**What would be the second most important thing for you to say something about? That you ...**

**READ OUT THE OPTIONS!**

**<< TEXT '1 ... has a particular religion? 'Of F223A = 1 >>**

**<< TEXT '2 ... born in a certain country? 'Of F223A = 2 >>**

**<< TEXT '3 ... have a particular gender? 'Of F223A = 3 >>**

**<< TEXT '4 ... has a certain political view? 'Of F223A = 4 >>**

**<< TEXT '5 ... has a particular sexual orientation? 'Of F223A = 5 >>**

**<< TEXT '6 ... belongs to a certain class of society? 'Of F223A = 6 >>**

**<< TEXT '7 ... has a certain style of music? 'Of F223A = 7 >>**

**<< TEXT '8 ... living in a particular area / site? 'Of F223A = 8 >>**

**<< TEXT '9 ... belong to a certain ethnic group? 'Of F223A = 9 >>**

**<< TEXT '10 ... or that you are rooting for a particular team? 'Of F223A = 10 >>**

**_______________________________________________________________________________**

**F223C**

**What would be the third most important for you to say something about? That you ...**

**READ OUT THE OPTIONS!**

**<< TEXT '1 ... has a particular religion? 'Of F223A + = 1 & F223B = 1 >>**

**<< TEXT '2 ... born in a certain country? 'Of F223A = 2 & F223B = 2 >>**

**<< TEXT '3 ... have a particular gender? 'Of F223A = 3 & F223B = 3 >>**

**<< TEXT '4 ... has a certain political view? 'Of F223A = 4 & F223B = 4 >>**

**<< TEXT '5 ... has a particular sexual orientation? 'Of F223A = 5 & F223B = 5 >>**

**<< TEXT '6 ... belongs to a certain class of society? 'Of F223A = 6 & F223B = 6 >>**

**<< TEXT '7 ... has a certain style of music? 'Of F223A = 7 & F223B = 7 >>**

**<< TEXT '8 ... living in a particular area / site? 'Of F223A = 8 & F223B = 8 >>**

**<< TEXT '9 ... belong to a certain ethnic group? 'Of F223A = 9 & F223B = 9 >>**

**<< TEXT '10 ... or that you are rooting for a particular team? 'Of F223A = 10 & F223B = 10 >>**

**_______________________________________________________________________________**

**F224**

**If you had to describe where you come from, it would be important to talk about**

**you're from a particular part of the world - that continent or continent -**

**or from a particular country, or from a particular part of the country, or from a particular place or city?**

**1 Some continent**

**2 given country**

**3 specific area**

**4 Some city**

**_______________________________________________________________________________**

**F224B**

**If you had to describe where you come from, what would be the second most important?**

**READ OUT THE OPTIONS!**

**<< TEXT '1 Some continent? 'Of F224 = 1 >>**

**<< TEXT '2 specific country? 'Of F224 = 2 >>**

**<< TEXT '3 range? 'Of F224 = 3 >>**

**<< TEXT '4 certain place? 'Of F224 = 4 >>**

**_______________________________________________________________________________**

**F231**

**People use different terms to describe their ethnicity.**

**What name do you describe yourself better?**

**Swedish 1**

**<< TEXT '2 Iranian 'Of Group =' IR '>>**

**<< TEXT '3 Kurd 'Of Group =' IR '>>**

**<< TEXT '4 Persian 'Of Group =' IR '>> << TEXT' 5 immigrants 'Of Group =' JU '>>**

**<< TEXT '5 immigrants 'Of Group =' IR '>> << TEXT' 6 Croatian 'Of Group =' JU '>>**

**<< TEXT ' 'Of Group =' IR '>> << TEXT' 7 Serb 'Of Group =' JU '>>**

**<< TEXT ' 'Of Group =' IR '>> << TEXT' 8 Finansloven 'Of Group =' JU '>>**

**<< TEXT '13 Other ... 'Of Group =' IR '>> << TEXT' 9 Bosnians 'Of Group =' JU '>>**

**<< TEXT ' 'Of Group =' IR '>> << TEXT '10 Kosovo Alban 'Of Group =' JU '>>**

**<< TEXT ' 'Of Group =' IR '>> << TEXT '11 Macedonians 'Of Group =' JU '>>**

**<< TEXT ' 'Of Group =' IR '>> << TEXT '12 Yugoslavian 'Of Group =' JU '>>**

**<< TEXT ' 'Of Group =' IR '>>**

**<< TEXT ' 'On Tour =' IR '>> << TEXT '13 Other ... 'Of Group =' JU '>>**

**_______________________________________________________________________________**

**F231TXT**

**(People use different terms to describe their ethnicity.**

**What name do you describe yourself better? )**

**ENTERING ANOTHER TITLE:**

**_______________________________________________________________________________**

**F231B**

**Is there any other element you can also fit in on yourself?**

**_______________________________________________________________________**

**TO IVE: If the UP specify more than one name, asking DPs choose the one that fits best.**

**_______________________________________________________________________**

**0 NO, no further TITLE MaTches**

**<< TEXT 'Swedish 1 'Of F231 = 1 >>**

**<< TEXT '2 Iranian 'Of Group =' IR '& F231 = 2 >>**

**<< TEXT '3 Kurd 'Of Group =' IR '& F231 = 3 >>**

**<< TEXT '4 Persian 'Of Group =' IR '& F231 = 4 >> << TEXT' 5 immigrants 'Of Group =' JU '& F231 = 5 >>**

**<< TEXT '5 immigrants 'Of Group =' IR '& F231 = 5 >> << TEXT' 6 Croatian 'Of Group =' JU '& F231 = 6 >>**

**<< TEXT ' 'Of Group =' IR '>> << TEXT' 7 Serb 'Of Group =' JU '& F231 = 7 >>**

**<< TEXT ' 'Of Group =' IR '>> << TEXT' 8 Finansloven 'Of Group =' JU '& F231 = 8 >>**

**<< TEXT '13 Other ... 'Of Group =' IR '>> << TEXT' 9 Bosnians 'Of Group =' JU '& F231 = 9 >>**

**<< TEXT ' 'Of Group =' IR '>> << TEXT '10 Kosovo Alban 'Of Group =' JU '& F231 = 10 >>**

**<< TEXT ' 'Of Group =' IR '>> << TEXT '11 Macedonians 'Of Group =' JU '& F231 = 11 >>**

**<< TEXT ' 'Of Group =' IR '>> << TEXT '12 Yugoslavian 'Of Group =' JU '& F231 = 12 >>**

**<< TEXT ' 'Of Group =' IR '>>**

**<< TEXT ' 'On Tour =' IR '>> << TEXT '13 Other ... 'Of Group =' JU '& F231 = 13 >>**

**_______________________________________________________________________________**

**F231BTXT**

**(Is there any other element you also think applies to you yourself?)**

**ENTERING ANOTHER TITLE:**

**_______________________________________________________________________________**

**F232B**

**I will now read out some different statements and want you to say to what degree**

**you agree with the statement on a scale from 1 to 5, where 1 means strongly disagree**

**and 5 means totally agree.**

**STATEMENT:**

**It is important for me to learn a lot about the Swedish culture, traditions and values.**

**1 2 3 4:05 a.m.**

**| --------------- | --------------- | --------------- | - -------------- |**

**agree**

**not entirely**

**_______________________________________________________________________________**

**F232C**

**STATEMENT:**

**I try to follow the Swedish customs and traditions.**

**1 2 3 4:05 a.m.**

**| --------------- | --------------- | --------------- | - -------------- |**

**agree**

**not entirely**

**_______________________________________________________________________________**

**F232D**

**STATEMENT:**

**I want my children to be brought up in accordance with Swedish traditions.**

**1 2 3 4:05 a.m.**

**| --------------- | --------------- | --------------- | - -------------- |**

**agree**

**not entirely**

**_______________________________________________________________________________**

**F232E**

**STATEMENT:**

**I feel pride when Swedes are successful in such sports or music.**

**1 2 3 4:05 a.m.**

**| --------------- | --------------- | --------------- | - -------------- |**

**agree**

**not entirely**

**_______________________________________________________________________________**

**F232G**

**To what extent do you feel affinity with Swedish culture and traditions?**

**Responding to a scale of 1 to 5 where 1 means no affinity at all and**

**5 means great affinity.**

**1 2 3 4:05 a.m.**

**| --------------- | --------------- | --------------- | - -------------- |**

**no big**

**affinity affinity**

**_______________________________________________________________________________**

**F233B**

**Now, following more allegations and I want you to say to what extent you agree**

**the statement on a scale from 1 to 5, where 1 means strongly disagree and**

**5 means totally agree.**

**STATEMENT:**

**It is important for me to learn a lot about my parents' home country**

**culture, traditions and values.**

**1 2 3 4:05 a.m.**

**| --------------- | --------------- | --------------- | - -------------- |**

**agree**

**not entirely**

**_______________________________________________________________________________**

**F233C**

**STATEMENT:**

**I try to follow the customs and traditions that are common in my parents' home country.**

**1 2 3 4:05 a.m.**

**| --------------- | --------------- | --------------- | - -------------- |**

**agree**

**not entirely**

**_______________________________________________________________________________**

**F233D**

**STATEMENT:**

**I want my children to be brought up in accordance with the traditions that are common in**

**my parents' homeland.**

**1 2 3 4:05 a.m.**

**| --------------- | --------------- | --------------- | - -------------- |**

**agree**

**not entirely**

**_______________________________________________________________________________**

**F233E**

**STATEMENT:**

**I feel proud when people from the same country as my parents**

**is successful in e.g. sports or music.**

**1 2 3 4:05 a.m.**

**| --------------- | --------------- | --------------- | - -------------- |**

**agree**

**not entirely**

**_______________________________________________________________________________**

**F233F**

**STATEMENT:**

**It is important for me to marry someone from my parents' home country.**

**1 2 3 4:05 a.m.**

**| --------------- | --------------- | --------------- | - -------------- |**

**agree**

**not entirely**

**_______________________________________________________________________________**

**F233G**

**Then I would also question the degree to which you feel connected with your parents**

**native culture and traditions?**

**Responding to a scale of 1 to 5 where 1 means no affinity at all and**

**5 means great affinity.**

**1 2 3 4:05 a.m.**

**| --------------- | --------------- | --------------- | - -------------- |**

**no big**

**affinity affinity**

**_______________________________________________________________________________**

**F233BM**

**Now, following more allegations and I want you to say to what extent you agree**

**the statement on a scale from 1 to 5, where 1 means strongly disagree and**

**5 means totally agree.**

**STATEMENT:**

**It is important for me to learn a lot about my mother's homeland**

**culture, traditions and values. ~~~~~~~**

**1 2 3 4:05 a.m.**

**| --------------- | --------------- | --------------- | - -------------- |**

**agree**

**not entirely**

**_______________________________________________________________________________**

**F233BP**

**Now, following more allegations and I want you to say to what extent you agree**

**the statement on a scale from 1 to 5, where 1 means strongly disagree and**

**5 means totally agree.**

**STATEMENT:**

**It is important for me to learn a lot about my dad's homeland**

**culture, traditions and values. ~~~~~~**

**1 2 3 4:05 a.m.**

**| --------------- | --------------- | --------------- | - -------------- |**

**agree**

**not entirely**

**_______________________________________________________________________________**

**F233CM**

**STATEMENT:**

**I try to follow the customs and traditions that are common in my mother's homeland.**

**~~~~~~~**

**1 2 3 4:05 a.m.**

**| --------------- | --------------- | --------------- | - -------------- |**

**agree**

**not entirely**

**_______________________________________________________________________________**

**F233CP**

**STATEMENT:**

**I try to follow the customs and traditions that are common in my father's homeland.**

**~~~~~~**

**1 2 3 4:05 a.m.**

**| --------------- | --------------- | --------------- | - -------------- |**

**agree**

**not entirely**

**_______________________________________________________________________________**

**F233DM**

**STATEMENT:**

**I want my children to be brought up in accordance with the traditions that are common in my mother's homeland.**

**~~~~~~~**

**1 2 3 4:05 a.m.**

**| --------------- | --------------- | --------------- | - -------------- |**

**agree**

**not entirely**

**_______________________________________________________________________________**

**F233DP**

**STATEMENT:**

**I want my children to be brought up in accordance with the traditions that are common in my father's homeland.**

**~~~~~~**

**1 2 3 4:05 a.m.**

**| --------------- | --------------- | --------------- | - -------------- |**

**agree**

**not entirely**

**_______________________________________________________________________________**

**F233EM**

**STATEMENT:**

**I feel proud when people from the same country as my mother**

**is successful in e.g. sports or music. ~~~~~~**

**1 2 3 4:05 a.m.**

**| --------------- | --------------- | --------------- | - -------------- |**

**agree**

**not entirely**

**_______________________________________________________________________________**

**F233EP**

**STATEMENT:**

**I feel proud when people from the same country as my father**

**is successful in e.g. sports or music. ~~~~~**

**1 2 3 4:05 a.m.**

**| --------------- | --------------- | --------------- | - -------------- |**

**agree**

**not entirely**

**_______________________________________________________________________________**

**F233FM**

**STATEMENT:**

**It is important for me to marry someone from my mother's homeland.**

**~~~~~~~**

**1 2 3 4:05 a.m.**

**| --------------- | --------------- | --------------- | - -------------- |**

**agree**

**not entirely**

**_______________________________________________________________________________**

**F233FP**

**STATEMENT:**

**It is important for me to marry someone from my father's homeland.**

**~~~~~~**

**1 2 3 4:05 a.m.**

**| --------------- | --------------- | --------------- | - -------------- |**

**agree**

**not entirely**

**_______________________________________________________________________________**

**F233GM**

**Then I would also question the degree to which you feel connected to your mother**

**native culture and traditions? ~~~~~~~**

**Responding to a scale of 1 to 5 where 1 means no affinity at all and**

**5 means great affinity.**

**1 2 3 4:05 a.m.**

**| --------------- | --------------- | --------------- | - -------------- |**

**no big**

**affinity affinity**

**_______________________________________________________________________________**

**F233GP**

**Then I would also question the degree to which you feel connected to your dad**

**native culture and traditions? ~~~~~~**

**Responding to a scale of 1 to 5 where 1 means no affinity at all and**

**5 means great affinity.**

**1 2 3 4:05 a.m.**

**| --------------- | --------------- | --------------- | - -------------- |**

**no big**

**affinity affinity**

**_______________________________________________________________________________**

**F235**

**How often do you attend religious activities in the church, mosque, synagogue or similar?**

**READ OUT THE OPTIONS!**

**Each day 1**

**2 More than once a week**

**3 About once a week**

**4 More than once a month**

**5 More rarely**

**6 Never**

**_______________________________________________________________________________**

**F236A**

**How religious would you say you are?**

**1 Very religious**

**2 Fairly religious**

**3 Little religious**

**4 Not at all religious**

**_______________________________________________________________________________**

**F236B**

**What religion do you belong to?**

**1 Protestant Christianity**

**2 Catholic Christianity**

**3 Orthodox Christianity**

**4 Frikyrklig (Incl Mormon, Witness etc.)**

**5 Kristendom, unspecified**

**6 Islam, Shia (Muslim = Islam)**

**7 Islam, Sunni (Muslim = Islam)**

**8 Islam - unspecified (Muslim = Islam)**

**9 Judaism**

**... 10 Other**

**_______________________________________________________________________________**

**F236BTXT**

**(What religion do you belong to?)**

**ENTERING ANOTHER RELIGION:**

**_______________________________________________________________________________**

**F237**

**Do you participate regularly in any organized activity (which meet at certain times**

**to practice, practice, or discuss anything)?**

**1 Yes**

**No. 2**

**_______________________________________________________________________________**

**F238**

**How many times a week you participate in such activities (a normal week)?**

**NUMBER OF TIMES A WEEK:**

**_______________________________________________________________________________**

**F239A**

**Did you vote in the 2010 elections to the parliament?**

**1 Yes**

**No. 2**

**3 UP non-voting**

**_______________________________________________________________________________**

**F239B**

**Did you vote in the 2010 elections to the city council?**

**1 Yes**

**No. 2**

**_______________________________________________________________________________**

**F240A**

**If it were the general election today, would you vote for in the election?**

**1 Yes**

**No. 2**

**3 UP non-voting**

**_______________________________________________________________________________**

**F240B**

**If it were local elections today, would you vote for in the election?**

**1 Yes**

**No. 2**

**_______________________________________________________________________________**

**F241**

**About how many hours do you watch TV on a typical day? (Including DVD and video.)**

**NUMBER OF HOURS PER DAY:**

**_______________________________________________________________________________**

**F281**

**Do you go to the movies?**

**1 About once a week or more often**

**2, one or several times a month**

**3, one or several times in quarter**

**4 More seldom or never**

**_______________________________________________________________________________**

**F281B**

**What is the best movie you've seen in your entire life?**

**If you can not think of what you think is best,**

**Name a film or director that you really like.**

**FILM AND DIRECTOR:**

**_______________________________________________________________________________**

**F282**

**How often do you go to the theater?**

**1 About once a week or more often**

**2, one or several times a month**

**3, one or several times in quarter**

**4 More seldom or never**

**_______________________________________________________________________________**

**F283**

**How often do you go to concerts?**

**1 About once a week or more often**

**2, one or several times a month**

**3, one or several times in quarter**

**4 More seldom or never**

**_______________________________________________________________________________**

**F283B**

**What is your favorite artist or group?**

**If you can not be who you think is best,**

**Name one artist or group that you really like?**

**ARTIST OR GROUP:**

**_______________________________________________________________________________**

**F284**

**How often do you go to a museum?**

**1 About once a week or more often**

**2, one or several times a month**

**3, one or several times in quarter**

**4 More seldom or never**

**_______________________________________________________________________________**

**F285**

**How often do you go to the library in your spare time?**

**1 About once a week or more often**

**2, one or several times a month**

**3, one or several times in quarter**

**4 More seldom or never**

**_______________________________________________________________________________**

**F286**

**Do you go as spectators in football, hockey, handball and other team sports?**

**1 About once a week or more often**

**2, one or several times a month**

**3, one or several times in quarter**

**4 More seldom or never**

**_______________________________________________________________________________**

**F286B**

**Which national team would cheer you on in the next World Cup, all teams were having?**

**ENTER national teams:**

**_______________________________________________________________________________**

**F288**

**How often do you go out and eat at a restaurant or tavern?**

**1 About once a week or more often**

**2, one or several times a month**

**3, one or several times in quarter**

**4 More seldom or never**

**_______________________________________________________________________________**

**F288B**

**What is your favorite dish?**

**ENTER DISH:**

**_______________________________________________________________________________**

**F288C**

**How often do you go to parties?**

**1 About once a week or more often**

**2, one or several times a month**

**3, one or several times in quarter**

**4 More seldom or never**

**_______________________________________________________________________________**

**F289A**

**How often do read a book that is not a textbook?**

**1 About once a week or more often**

**2, one or several times a month**

**3, one or several times in quarter**

**4 More seldom or never**

**_______________________________________________________________________________**

**F289B**

**What is the best book you read in your entire life?**

**If you can not think of what you think is best,**

**Name a book or author that you really like.**

**Title or author:**

**_______________________________________________________________________________**

**F289D**

**About how many books are there in your home?**

**__________________________________________________________**

**TO IVE: A normal bokhyllerad holds about 40 books and**

**a full bookcase about 200 books.**

**__________________________________________________________**

**Number of books:**

**_______________________________________________________________________________**

**F289E**

**How often do you play games on the PC and / or console?**

**1 About once a week or more often**

**2, one or several times a month**

**3, one or several times in quarter**

**4 More seldom or never**

**_______________________________________________________________________________**

**F2810**

**About how many people would you say you have contact with a normal day, on average?**

**Including all of you visiting and chatting with, whether it is face to face,**

**on the phone, via email or on the Internet, and whether you know the person or not.**

**__________________________________________________________________________**

**TO IVE: If the UP is difficult to estimate, suggest: "Is it a titoal or fifty?".**

**If the DP says such "Twenty-thirty", specify an agent host (in this case 25).**

**__________________________________________________________________________**

**NUMBER OF PEOPLE:**

**_______________________________________________________________________________**

**F291**

**Now I shall, to the end of the interview, read a number of statements.**

**For each statement, please answer how well it describes you.**

**STATEMENT:**

**I find it hard to sit still and concentrate.**

**READ OUT THE OPTIONS!**

**1 Do not agree at all to you**

**2 Compliant pretty bad to you**

**3 Do either good or bad to you**

**4 Is pretty good to you**

**5 apply to you**

**_______________________________________________________________________________**

**F292**

**STATEMENT:**

**I rarely cause trouble.**

**READ OUT THE OPTIONS!**

**1 Do not agree at all to you**

**2 Compliant pretty bad to you**

**3 Do either good or bad to you**

**4 Is pretty good to you**

**5 apply to you**

**_______________________________________________________________________________**

**F293**

**STATEMENT:**

**I am most willing to take risks.**

**READ OUT OPTIONS WHEN NEEDED!**

**1 Do not agree at all to you**

**2 Compliant pretty bad to you**

**3 Do either good or bad to you**

**4 Is pretty good to you**

**5 apply to you**

**_______________________________________________________________________________**

**F294**

**STATEMENT:**

**I am often tense and nervous.**

**READ OUT OPTIONS WHEN NEEDED!**

**1 Do not agree at all to you**

**2 Compliant pretty bad to you**

**3 Do either good or bad to you**

**4 Is pretty good to you**

**5 apply to you**

**_______________________________________________________________________________**

**F295**

**STATEMENT:**

**I feel many times that I have little influence over the things that happen to me.**

**READ OUT OPTIONS WHEN NEEDED!**

**1 Do not agree at all to you**

**2 Compliant pretty bad to you**

**3 Do either good or bad to you**

**4 Is pretty good to you**

**5 apply to you**

**_______________________________________________________________________________**

**F296**

**STATEMENT:**

**I often feel sad and down.**

**READ OUT OPTIONS WHEN NEEDED!**

**1 Do not agree at all to you**

**2 Compliant pretty bad to you**

**3 Do either good or bad to you**

**4 Is pretty good to you**

**5 apply to you**

**_______________________________________________________________________________**

**F297**

**STATEMENT:**

**I can do a lot.**

**READ OUT OPTIONS WHEN NEEDED!**

**1 Do not agree at all to you**

**2 Compliant pretty bad to you**

**3 Do either good or bad to you**

**4 Is pretty good to you**

**5 apply to you**

**_______________________________________________________________________________**

**F298**

**STATEMENT:**

**I can affect how my future will be.**

**READ OUT OPTIONS WHEN NEEDED!**

**1 Do not agree at all to you**

**2 Compliant pretty bad to you**

**3 Do either good or bad to you**

**4 Is pretty good to you**

**5 apply to you**

**_______________________________________________________________________________**

**F299**

**STATEMENT:**

**I think it's important for me to start a family.**

**READ OUT OPTIONS WHEN NEEDED!**

**1 Do not agree at all to you**

**2 Compliant pretty bad to you**

**3 Do either good or bad to you**

**4 Is pretty good to you**

**5 apply to you**

**_______________________________________________________________________________**

**F2910**

**STATEMENT:**

**I think it's important for me to go to university or college.**

**READ OUT OPTIONS WHEN NEEDED!**

**1 Do not agree at all to you**

**2 Compliant pretty bad to you**

**3 Do either good or bad to you**

**4 Is pretty good to you**

**5 apply to you**

**_______________________________________________________________________________**

**F2912**

**STATEMENT:**

**Seen on the whole, I'm happy.**

**READ OUT OPTIONS WHEN NEEDED!**

**1 Do not agree at all to you**

**2 Compliant pretty bad to you**

**3 Do either good or bad to you**

**4 Is pretty good to you**

**5 apply to you**

**_______________________________________________________________________________**

**F2913**

**STATEMENT:**

**I often feel lonely.**

**READ OUT OPTIONS WHEN NEEDED!**

**1 Do not agree at all to you**

**2 Compliant pretty bad to you**

**3 Do either good or bad to you**

**4 Is pretty good to you**

**5 apply to you**

**_______________________________________________________________________________**

**F2914**

**STATEMENT:**

**I try to avoid risks and take the safe way.**

**READ OUT OPTIONS WHEN NEEDED!**

**1 Do not agree at all to you**

**2 Compliant pretty bad to you**

**3 Do either good or bad to you**

**4 Is pretty good to you**

**5 apply to you**

**_______________________________________________________________________________**

**F2915**

**STATEMENT:**

**It is important for me to get a job where I earn a lot of money.**

**READ OUT OPTIONS WHEN NEEDED!**

**1 Do not agree at all to you**

**2 Compliant pretty bad to you**

**3 Do either good or bad to you**

**4 Is pretty good to you**

**5 apply to you**

**_______________________________________________________________________________**

**F2916**

**STATEMENT:**

**I believe that success is a result of hard work - luck has little or nothing to do with it.**

**READ OUT OPTIONS WHEN NEEDED!**

**1 Do not agree at all to you**

**2 Compliant pretty bad to you**

**3 Do either good or bad to you**

**4 Is pretty good to you**

**5 apply to you**

**_______________________________________________________________________________**

**F2917**

**STATEMENT:**

**I am mostly happy with myself.**

**READ OUT OPTIONS WHEN NEEDED!**

**1 Do not agree at all to you**

**2 Compliant pretty bad to you**

**3 Do either good or bad to you**

**4 Is pretty good to you**

**5 apply to you**

**_______________________________________________________________________________**

**F2918**

**STATEMENT:**

**I have great confidence.**

**READ OUT OPTIONS WHEN NEEDED!**

**1 Do not agree at all to you**

**2 Compliant pretty bad to you**

**3 Do either good or bad to you**

**4 Is pretty good to you**

**5 apply to you**

**_______________________________________________________________________________**

**F2919**

**STATEMENT:**

**I am often angry and irritable.**

**READ OUT OPTIONS WHEN NEEDED!**

**1 Do not agree at all to you**

**2 Compliant pretty bad to you**

**3 Do either good or bad to you**

**4 Is pretty good to you**

**5 apply to you**

**_______________________________________________________________________________**

**F2920**

**STATEMENT:**

**Dare I say what I think.**

**READ OUT OPTIONS WHEN NEEDED!**

**1 Do not agree at all to you**

**2 Compliant pretty bad to you**

**3 Do either good or bad to you**

**4 Is pretty good to you**

**5 apply to you**

**_______________________________________________________________________________**

**F2921**

**STATEMENT:**

**I'm influential.**

**READ OUT OPTIONS WHEN NEEDED!**

**1 Do not agree at all to you**

**2 Compliant pretty bad to you**

**3 Do either good or bad to you**

**4 Is pretty good to you**

**5 apply to you**

**_______________________________________________________________________________**

**F2922**

**STATEMENT:**

**I can control my life.**

**READ OUT OPTIONS WHEN NEEDED!**

**1 Do not agree at all to you**

**2 Compliant pretty bad to you**

**3 Do either good or bad to you**

**4 Is pretty good to you**

**5 apply to you**

**_______________________________________________________________________________**

**F2923**

**STATEMENT:**

**I'm happy with my appearance.**

**READ OUT OPTIONS WHEN NEEDED!**

**1 Do not agree at all to you**

**2 Compliant pretty bad to you**

**3 Do either good or bad to you**

**4 Is pretty good to you**

**5 apply to you**

**_______________________________________________________________________________**

**F2924**

**STATEMENT:**

**I like to decide.**

**READ OUT OPTIONS WHEN NEEDED!**

**1 Do not agree at all to you**

**2 Compliant pretty bad to you**

**3 Do either good or bad to you**

**4 Is pretty good to you**

**5 apply to you**

**_______________________________________________________________________________**

**F2925**

**STATEMENT:**

**I think I will get it well in the future.**

**READ OUT OPTIONS WHEN NEEDED!**

**1 Do not agree at all to you**

**2 Compliant pretty bad to you**

**3 Do either good or bad to you**

**4 Is pretty good to you**

**5 apply to you**

**_______________________________________________________________________________**

**F2926**

**STATEMENT:**

**I have no worries.**

**READ OUT OPTIONS WHEN NEEDED!**

**1 Do not agree at all to you**

**2 Compliant pretty bad to you**

**3 Do either good or bad to you**

**4 Is pretty good to you**

**5 apply to you**

**_______________________________________________________________________________**

**F2927**

**STATEMENT:**

**When I plan, I am sure that I can realize my plans.**

**READ OUT OPTIONS WHEN NEEDED!**

**1 Do not agree at all to you**

**2 Compliant pretty bad to you**

**3 Do either good or bad to you**

**4 Is pretty good to you**

**5 apply to you**

**_______________________________________________________________________________**

**F2928**

**STATEMENT:**

**I get very angry easily.**

**READ OUT OPTIONS WHEN NEEDED!**

**1 Do not agree at all to you**

**2 Compliant pretty bad to you**

**3 Do either good or bad to you**

**4 Is pretty good to you**

**5 apply to you**

**_______________________________________________________________________________**

**F2929**

**STATEMENT:**

**I think that what happens to me because of my own actions.**

**READ OUT OPTIONS WHEN NEEDED!**

**1 Do not agree at all to you**

**2 Compliant pretty bad to you**

**3 Do either good or bad to you**

**4 Is pretty good to you**

**5 apply to you**

**_______________________________________________________________________________**

**F2930**

**STATEMENT:**

**I would rather choose to be SEK 1,000 today than to get 2000 SEK for one year.**

**READ OUT OPTIONS WHEN NEEDED!**

**1 Do not agree at all to you**

**2 Compliant pretty bad to you**

**3 Do either good or bad to you**

**4 Is pretty good to you**

**5 apply to you**

**_______________________________________________________________________________**

**F2931**

**STATEMENT:**

**I wish I had more self-control.**

**READ OUT OPTIONS WHEN NEEDED!**

**1 Do not agree at all to you**

**2 Compliant pretty bad to you**

**3 Do either good or bad to you**

**4 Is pretty good to you**

**5 apply to you**

**_______________________________________________________________________________**

**F2932**

**STATEMENT:**

**I sometimes do things without thinking through the options.**

**READ OUT OPTIONS WHEN NEEDED!**

**1 Do not agree at all to you**

**2 Compliant pretty bad to you**

**3 Do either good or bad to you**

**4 Is pretty good to you**

**5 apply to you**

**_______________________________________________________________________________**

**F30B**

**There were all the questions I had.**

**The research team at Stockholm University has plans to continue**

**this study of young people's lives.**

**Therefore, once the researchers will be happy to those who participated in the survey**

**in a few years to see if and how the situation has changed.**

**Can you imagine that in a few years, take the time for a similar interview and**

**thereby contributing valuable knowledge to research on how young**

**people can more easily enter the labor market?**

**Even though you now are willing to participate and accept it - it means**

**not that you agree to participate.**

**If the research study is repeated, and SCB will call in a few years, you take**

**only then decide whether you want to participate in an interview or not.**

**_____________________________________________________________**

**TO IVE: For more information, see F4 (Show help).**

**_____________________________________________________________**

**1 YES, would consider DELTA**

**2 NO, NOT WANT DELTA**

**_______________________________________________________________________________**

**F30BB**

**In order to be able to contact you for a possible future study, Statistics Sweden would have to save the contact information that your social security number for later re-contact you - this information will not be disclosed to researchers at Stockholm University, or at all to anyone.**

**The information stored in the SCB only for the purpose that if something is able to contact you - because you then be able to decide whether you want to participate in a follow-up interview.**

**The data are protected according to Chapter 24. Section 8 of the Secrecy Act (2009: 400) and the Personal Data Act (1998: 204).**

**Can we save this contact information in order to re-contact you in a year, for a possible follow-up interview?**

**1 YES, data can be tracked**

**2 NO, DO NOT TO DATA SAVED**

**_______________________________________________________________________________**

**THANKS**

**There were all the questions I had. Warm thanks for your valuable contribution!**

**______________________________________________________________**

**TO IVE: End the call with the UP and then proceed to the IVE queries.**

**______________________________________________________________**

**Press 1 and ENTER.**

**_______________________________________________________________________________**

**F301**

**QUESTION TO THE INTERVIEWER:**

**How do you perceive the DP Swedish?**

**1 Fully fluent and correct Swedish**

**2 Some non-correct word orders**

**3 UP make themselves understood, but is faltering language and / or breaks sharply**

**4 Very poor presentation skills, hard to understand**

**5 Can not speak Swedish**

**_______________________________________________________________________________**

**F302**

**QUESTION TO THE INTERVIEWER:**

**How do you assess the reliability of this interview?**

**1 Satisfactory**

**2 Not satisfactory**

**3 Deficient**

**_______________________________________________________________________________**

**F303**

**QUESTION TO THE INTERVIEWER:**

**What is / are circumstances gave you reason to suspect that the reliability is not**

**fully satisfactory or inadequate?**

**DESCRIBE (PRECIS):**

**_______________________________________________________________________________**

**F304**

**QUESTION TO THE INTERVIEWER:**

**Are there any particular areas of the form where reliability seems**

**particularly low in this interview?**

**____________________________________________________________**

**TO IVE: If no specific fields, write NO in the clear.**

**____________________________________________________________**

**DESCRIBE (PRECIS):**

**_______________________________________________________________________________**

**FINAL**

**QUESTION TO THE INTERVIEWER:**

**Is UP residing or working abroad?**

**______________________________________________**

**TO IVE: If yes, press F2, and then note the country.**

**______________________________________________**

**1 Yes**

**No. 2**

**F8 = DK**

**_______________________________________________________________________________**
